# Hydrosulfide (HS^−^) Recognition and Sensing in Water by Halogen Bonding Hosts

**DOI:** 10.1002/anie.202110442

**Published:** 2021-10-05

**Authors:** Edward J. Mitchell, Adam J. Beecroft, Jonathan Martin, Sally Thompson, Igor Marques, Vítor Félix, Paul D. Beer

**Affiliations:** ^1^ Department of Chemistry University of Oxford Chemistry Research Laboratory Mansfield Road Oxford OX1 3TA UK; ^2^ CICECO—Aveiro Institute of Materials Department of Chemistry University of Aveiro 3810-193 Aveiro Portugal; ^3^ Radioactive Waste Management Building 329, Thomson Avenue, Harwell Campus Didcot OX11 0GD UK

**Keywords:** coumarin, fluorescent sensing, halogen bonding, hydrosulfide, anion recognition

## Abstract

Hydrogen sulfide (H_2_S) plays a crucial signalling role in a variety of physiological systems, existing as the hydrosulfide anion (HS^−^) at physiological pH. Combining the potency of halogen bonding (XB) for anion recognition in water with coumarin fluorophore incorporation in acyclic host structural design, the first XB receptors to bind and, more importantly, sense the hydrosulfide anion in pure water in a reversible chemosensing fashion are demonstrated. The XB receptors exhibit characteristic selective quenching of fluorescence upon binding to HS^−^. Computational DFT and molecular dynamics simulations in water corroborate the experimental anion binding observations, revealing the mode and nature of HS^−^ recognition by the XB receptors.

## Graphical abstract

Reversible chemosensing of hydrosulfide (HS^−^) in pure water is demonstrated using halogen bonding (XB) fluorescent hosts. The XB receptors exhibit characteristic selective quenching of fluorescence upon binding HS^−^ whereas the hydrogen bonding receptor analogues proved incapable of functioning as chemosensors under aqueous conditions.

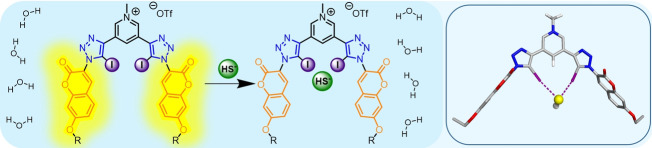

Anions perform critical roles in a plethora of biological, medicinal and anthropogenic environmental processes, inspiring the burgeoning field of anion supramolecular chemistry.^[1]^ Overcoming competitive anion hydration is a key challenge in the design of host systems capable of the molecular recognition of anion guest species in aqueous media. Highly charged polycationic receptors, Lewis acidic main group and metal based hosts, together with cyclic peptides and hydrophobic cage‐like hosts have been the leading effective strategies to date.[^1^, ^2^, ^3^]

The toxicity of hydrogen sulfide (H_2_S) in the environment and towards human health is well known. Furthermore, this simple molecule has recently been recognised as a third gasotransmitter, along with carbon monoxide and nitric oxide, playing crucial signalling physiological functions in the regulation of cardiovascular, immune, endocrine and nervous systems.[^4^, ^5^, ^6^, ^7^] In addition, abnormal H_2_S concentrations have been implicated in a number of medical conditions.[^8^, ^9^] With a p*K*_a_ of 7.0, at physiological pH, the hydrosulfide anion (HS^−^) is the dominant species of H_2_S in aqueous solution. Methods for the detection of these analytes have been mainly confined to irreversible chemodosimeter approaches.[^10^, ^11^] Surprisingly, it is only very recently that a reversible supramolecular host–guest approach to binding HS^−^ has been reported in organic solvent media using acyclic bis‐urea^[12]^ and tripodal amide receptors,^[13]^ and with bambusuril macrocycles in water.[^14^, ^15^] A recent addition to the anion‐binding supramolecular toolbox is halogen bonding (XB), a highly directional, attractive interaction between an electron‐deficient halogen atom, and a Lewis base.[^16^, ^17^] The few acyclic and macrocyclic XB receptors reported thus far commonly exhibit superior anion binding affinities, as well as enhanced sensory performance, compared to HB analogues,[^18^, ^19^, ^20^, ^21^] with examples of XB host systems that function in aqueous media and especially pure water being notably rare.[^20^, ^22^]

Herein, we report the first XB receptors to recognise and most importantly sense HS^−^ in pure water using an optical fluorescent chemosensing method. Three target XB host designs (Figure 1) were developed, each containing two coumarin fluorophores incorporated into a XB bis‐iodotriazole pyridine, pyridinium or secondary amine motif, where water solubility is achieved by the functionalisation of the coumarin groups with multiple triethylene glycol (TEG) substituents.

**Figure 1:**
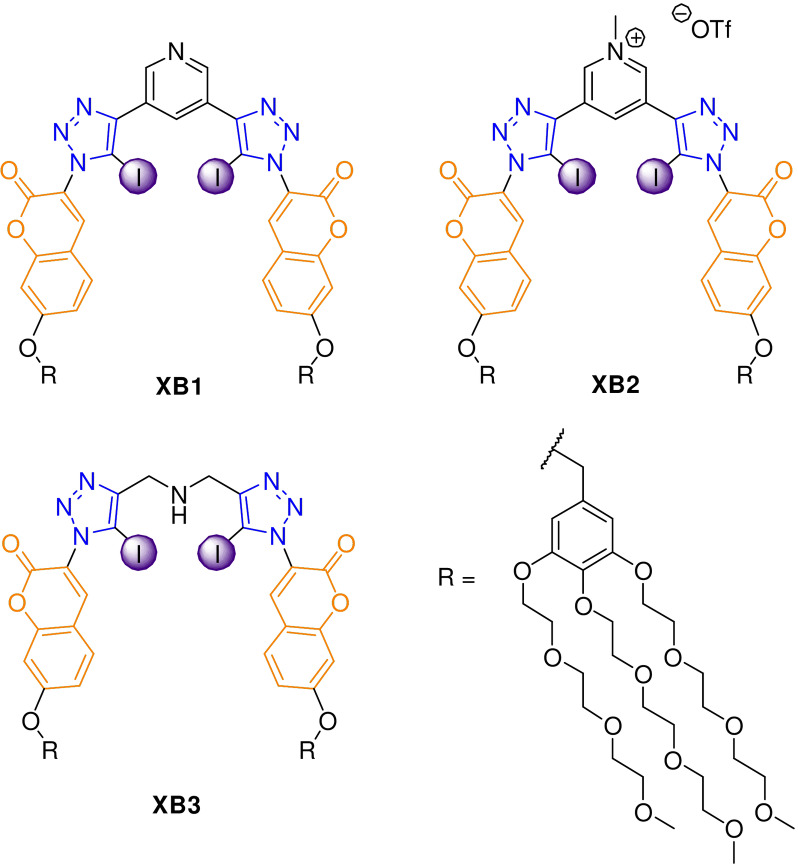
Target water soluble halogen bonding host designs.

The synthesis of the novel hosts is outlined in Scheme 1. 7‐Hydroxycoumarin **1** was reacted with 1.2 equivalents of the appropriate chloro‐benzyl PEG‐functionalised derivative under basic conditions in the presence of NaI to afford **2** in 75 % yield. Boc deprotection of **2** with TFA produced amine **3** which, upon sequential diazotisation using NaNO_2_ and addition of NaN_3_ in acidic aqueous solution, afforded coumarin‐azide **4** in yields of 94 %. The copper(I)‐catalysed azide–iodoalkyne cycloaddition (CuAAC) reaction of two equivalents of coumarin‐azide **4** with one equivalent of 3,5‐bis‐iodoalkyne pyridine **5** or *N*‐Boc‐protected bis‐iodoalkyne **6** in the presence of a [Cu(CH_3_CN)_4_]PF_6_ catalyst and TBTA afforded **XB1** and Boc‐protected bis‐triazole derivative **7** in 75 % and 59 % yields, respectively. Methylation of **XB1** to produce the pyridinium receptor **XB2** was achieved via reaction with excess methyl iodide, followed by anion exchange with sodium trifluoromethanesulfonate. Boc deprotection of **7** was achieved using TFA to afford **XB3**.

**Scheme 1:**
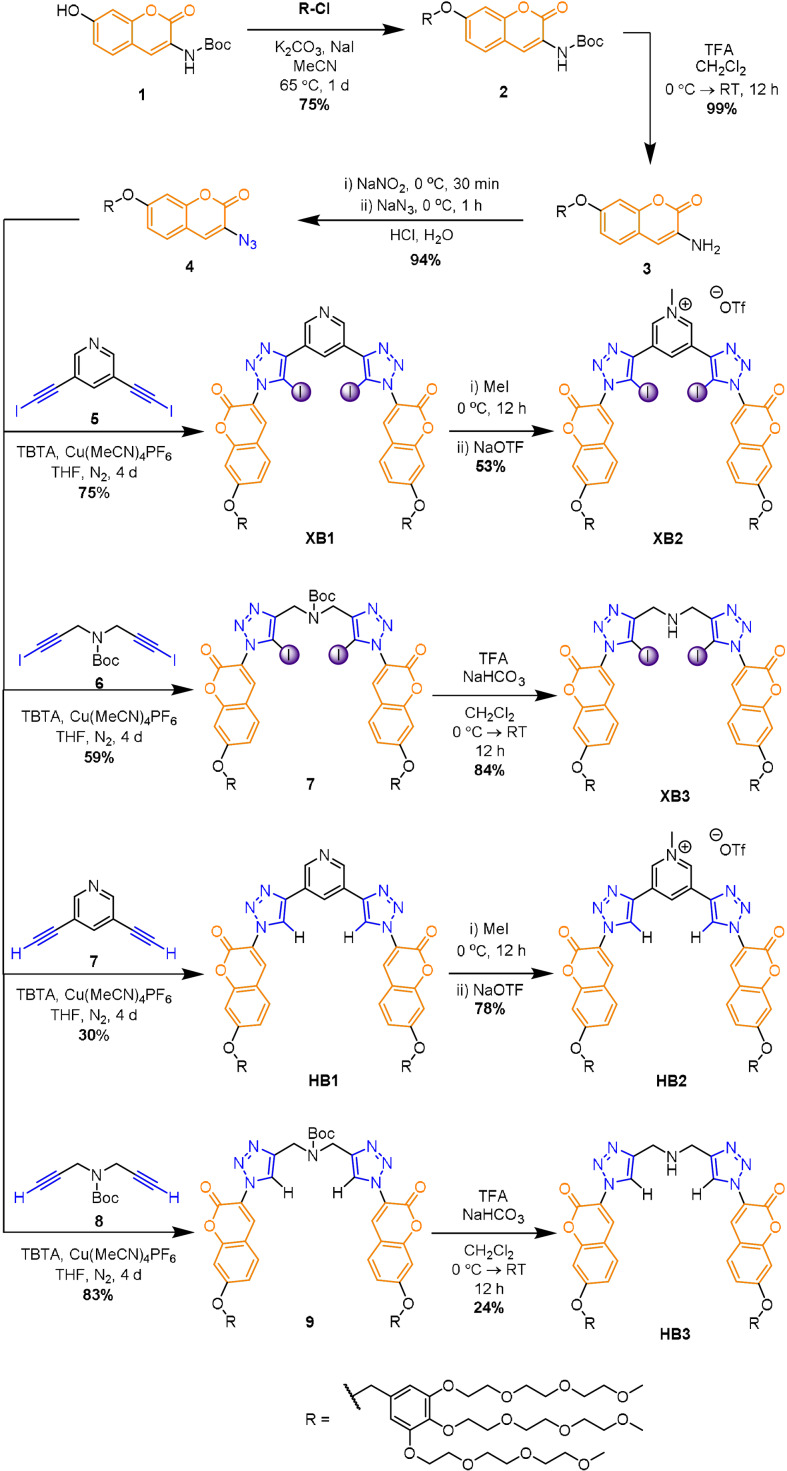
Synthesis of receptors **XB1**–**3** and **HB1**–**3**.

Analogous synthetic methods were used to prepare the corresponding hydrogen bonding hosts **HB1**–**3** (Scheme 1). All receptors were characterised by ^1^H and ^13^C NMR spectroscopy, high resolution mass spectrometry, UV/Vis and fluorescence spectroscopy (see SI).

The anion sensing properties of all receptors were investigated in pH 7.4 aqueous HEPES buffer solution using fluorescence spectroscopy.^[23]^ At this pH value the protonated forms **XB3H^+^** and **HB3H^+^** are the dominant receptor species present in the aqueous solution.^[24]^

The receptors’ fluorescence emission, following excitation at 342 nm, was monitored upon the addition of sodium hydrosulfide and halide salts. Remarkably, the fluorescence intensity of **XB1** was significantly quenched by 60 % in the presence of 10 equivalents of HS^−^, while I^−^, Br^−^ and Cl^−^ induced no intensity diminutions (Figure 2 a,b and Figure S3‐1). In stark contrast, **HB1** exhibited only minor fluorescence perturbations (Figure S3‐2) suggesting very weak binding which notably highlights the superior capability of XB donor motifs for anion recognition and sensing in water in comparison to HB donor analogues.

**Figure 2:**
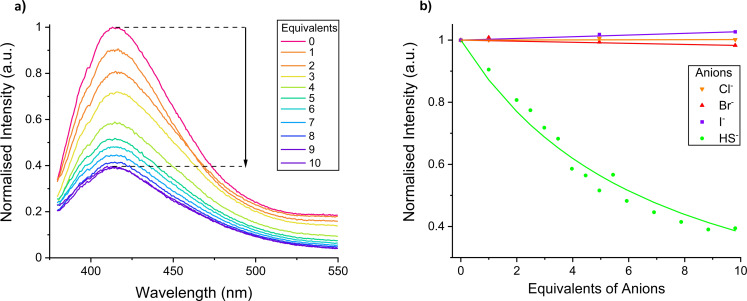
a) Fluorescence emission change of 10 μm **XB1** on addition of NaHS in pH 7.4 10 mm aqueous HEPES buffer solution. b) Binding isotherms and Bindfit analysis[^25^, ^26^] fit of fluorescence titration data monitoring *λ*_max_ (400–420 nm) of receptor **XB1** upon addition of NaCl, NaBr, NaI and NaHS in pH 7.4 10 mm aqueous HEPES buffer solution.

Similarly, addition of HS^−^ to **XB2** and **XB3H^+^** caused notable quenching, 45 % and 38 %, respectively, whereas no change was observed with Cl^−^. With **XB2**, both Br^−^ and I^−^ were sensed, with the heavier halide also being detected by **XB3H^+^** (Figure S3‐5). In contrast, the hydrogen bonding analogue **HB3H^+^** was found to display no fluorescent response to any anion (Figure S3‐6). However, **HB2** exhibited a selective fluorescence intensity decrease of 22 % with addition of 100 equivalents of I^−^ (Figure S3‐4). The quenching observed may be attributed to photoinduced electron transfer (PET) between the coumarin fluorophore and the bound anion.

Bindfit analysis[^25^, ^26^] of the titration data for XB and HB receptors was used to determine 1:1 host/guest stoichiometric anion association constants (Table 1).

**Table 1:** Anion association constants and limits of detection of HS^−^ for receptors.

Host	Association constant *K*_a_ [m ^−1^]^[a,b]^				LoD [μm]
	Cl^−^	Br^−^	I^−^	HS^−^	
**XB1**	NB	NB	NB	16 500 (1600)	14.3
**XB2**	NB	60 800 (7800)	103 000 (24 000)	158 000 (31 000)	6.7
**XB3H^+^**	NB	NB	46 000 (14 000)	5600 (530)	24.6
**HB1**	NB	NB	NB	NB	NB
**HB2**	NB	NB	6080 (550)	NB	NB
**HB3H^+^**	NB	NB	NB	NB	NB

NB—No binding observed. LoD—Limit of Detection for HS^−^. [a] Anion association constants (with errors in brackets), determined using Bindfit analysis[^23^, ^24^] of the fluorescence titration data monitoring averaged *λ*_max_ (400–420 nm). [b] 10 μm receptors in 10 mm aqueous HEPES buffer solution, pH 7.4.

Notably, neutral **XB1** behaves as an exclusive chemosensor for HS^−^, exhibiting strong and selective recognition for HS^−^ over the halides, which are not bound. As expected, the magnitude of HS^−^ association by the positively charged pyridinium receptor **XB2** is significantly greater compared to neutral **XB1** due to additional favourable electrostatic interactions. However, the increase in binding strength comes at the expense of a diminished degree of selectivity for HS^−^ as **XB2** also binds Br^−^ and, in particular, I^−^ strongly. **XB3H^+^** exhibits impressive HS^−^ selectivity over the lighter halides but forms the strongest association with I^−^.[^27^, ^28^]

Importantly, the limits of detection (LoD) for HS^−^ for **XB1**–**3** were calculated to be in the low micromolar range (Table 1, Figure S7–1), comparable to chemodosimeter probes used for detecting H_2_S in aqueous environments and in vitro studies.^[29]^

However, in comparison to chemodosimeters, **XB1**–**XB3** are capable of acting as reversible chemosensors; reversibility studies were carried out by removal of HS^−^ using Zn(OTf)_2_. Upon addition of the zinc salt to fluorescence HS^−^‐quenched solutions of **XB1**–**XB3**, the respective receptor's fluorescence returned to near non‐quenched emissions as ZnS precipitated, proving the receptors’ chemosensing reversible capability (Figure S4‐1).

The geometric and energetic properties of XB interactions between HS^−^ and I^−^ with **XB1** and **XB2** were evaluated using DFT calculations performed at M06‐2X/Def2‐TZVP/C‐PCM (water) on model complexes in which the large TEG‐substituted aryl moieties were replaced with smaller ethyl groups, affording the **XB1_Et_** and **XB2_Et_** prototypes (see SI for further computational details). The optimised structures of the HS^−^ and I^−^ associations with **XB1_Et_** and **XB2_Et_** are presented in Figure 3.

**Figure 3:**
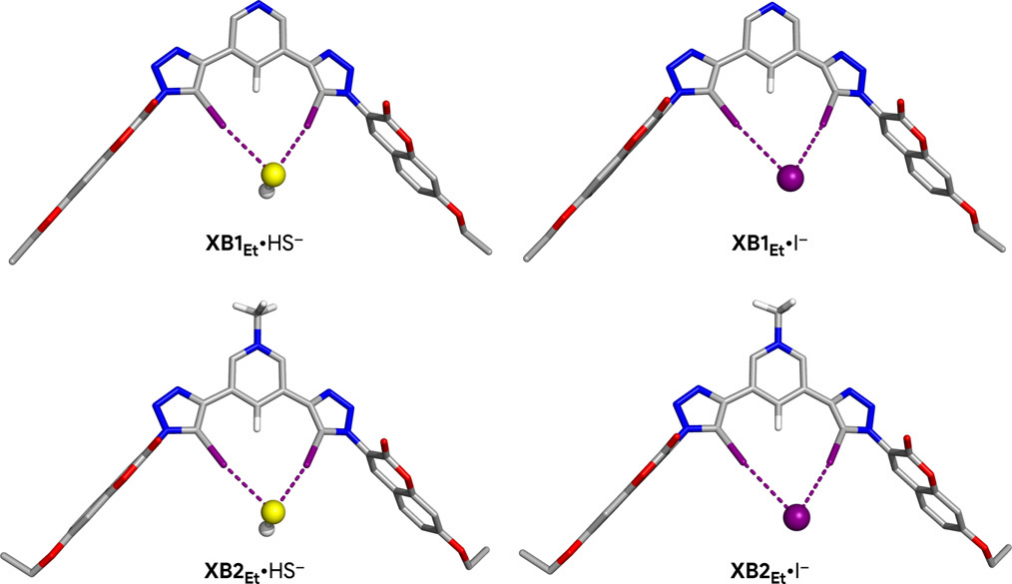
DFT‐optimised structures of **XB1_Et_** and **XB2_Et_** model receptors halogen‐bonded to the HS^−^ and I^−^ anions. The XB interactions are sketched as purple dashed lines.

The model receptors adopt a putative binding conformation with the iodotriazole binding units establishing two convergent, almost linear, XB interactions with C−I⋅⋅⋅I^−^ and C−I⋅⋅⋅HS^−^ angles around 171° (see Table S9‐1). The I⋅⋅⋅SH^−^ distances are markedly shorter than the I⋅⋅⋅I^−^ ones with average values of 3.173±0.032 and 3.580±0.020 Å, respectively, mainly mirroring the ion volumes, estimated as 58.78 Å^3^ for HS^−^ and 70.48 Å^3^ for I^−^.^[30]^ The XB interactions are slightly shorter by ca. 0.05 Å in charged **XB2_Et_** than in the associations with neutral **XB1_Et_**, regardless of the anion. Noteworthy, the HS^−^ anion is nearly perpendicular to the iodotriazole binding units, intercepting the plane defined by the two C−I bonds at a tilt angle of 90.7 and 93.0° for **XB1_Et_**_,_ and **XB2_Et_**_,_ respectively, in agreement with the anisotropic distribution of the electrostatic potential of HS^−^ (see Figure S9‐1). Overall, the *V*_S_ of the free XB‐based model receptors yields well‐defined regions of high positive electrostatic potential in front of both iodine binding sites (see Figure S9‐2), enabling anion recognition through nearly linear XB interactions.

The binding free energies (Δ*G* ^SS^) between **XB1_Et_** or **XB2_Et_** and I^−^ or HS^−^ were estimated as the energy differences between the associations and the free model receptors and anions, as detailed in the Supporting Information. The binding enthalpies (Δ*H*, given in Table S9‐2), of −13.91 and −16.30 kcal mol^−1^ for the HS^−^ associations with **XB1_Et_**, and **XB2_Et_**, are in line with the shorter intermolecular distances computed for the C−I⋅⋅⋅SH^−^ halogen bonds, while the I^−^ associations with the neutral and charged model receptors have Δ*H* values of −8.56 and −10.00 kcal mol^−1^, respectively, mirroring the longer C−I⋅⋅⋅I^−^ distances. The estimation of the binding entropy penalties leads to the Δ*G* ^SS^ values listed in Table S9‐2, which indicate an indubitable binding preference of both model receptors for HS^−^, with a *V*_S,min_ of −142.84 kcal mol^−1^. Moreover, the Δ*G* ^SS^ value of −0.77 kcal mol^−1^ for the binding interaction between **XB1_Et_** and I^−^ (with a *V*_S,min_ of −125.35 kcal mol^−1^) is almost negligible, in agreement with the inability of neutral **XB1** to bind this halide in water (see Table 1).

The strength of the XB interactions on the optimised model complexes in water medium was further comprehensively evaluated using different bond analysis methods. With the Natural Bond Orbital (NBO) analysis,^[31]^ the Second Order perturbation theory interaction energies (*E* ^2^) were estimated between the C−I antibonding orbitals of the model receptors and the anions’ lone pair orbitals (*n*_A_→σ*_C−I_), together with the variations in the occupancies of these orbitals. In agreement with interaction energies, the *E* ^2^ values listed in Table S9‐3 show that the XB interactions are stronger in the HS^−^ associations with **XB1_Et_**, and **XB2_Et_**, and naturally higher for the positively charged pyridinium‐based model receptor. Indeed, the *E* ^2^ values for the individual interactions with I^−^ of 9.19 and 10.48 kcal mol^−1^ for **XB1_Et_**, and **XB2_Et_**, respectively, are significantly smaller than the values for the interactions with HS^−^, 17.45 and 21.16 kcal mol^−1^, in the same order. These stronger interactions also lead to a change in the σ*_C−I_ orbital occupancies upon anion binding (see Table S9‐3), being more pronounced in the associations with HS^−^. Within the scope of the Quantum Theory of Atoms in Molecules^[32]^ (QTAIM), the potential energy density (*V*(r)) and Lagrangian kinetic energy density (*G*(r)) of the XB interactions’ bond critical points were used to estimate the energies of these halogen bonds (see Table S9‐4).^[33]^ The values of these two binding descriptors for a given model receptor with HS^−^ are ca. two times larger than with I^−^, following the same trend of the *E* ^2^ energies (*R* ^2^≥0.99, see Figure S9‐3), showing the equivalency between the QTAIM and NBO analyses. Furthermore, the |*V*(r)|/*G*(r) ratio gives insights into the nature of the XB interactions.^[34]^ Thus, the XB interactions of **XB1_Et_** and **XB2_Et_** with I^−^ are highly electrostatic, with ratios of ca. 0.93, while the interactions with HS^−^, with ratios around 1.04, indicate a slight increase of the covalency degree on the associations with this more nucleophilic anion (see Table S9‐4).

The nature of the halogen bonds was also ascertained in gas phase through Symmetry‐Adapted Perturbation Theory (SAPT) analysis (see SI),^[35]^ with the assessment of the SAPT interaction energy for each XB association. The values of the electrostatic, exchange, induction, and dispersion contributions are listed in Table S9‐5. Regardless of the anion, the electrostatic term is the largest component in the associations of charged **XB2_Et_**, whilst for the complexes with neutral **XB1_Et_** it is the repulsive exchange term. Moreover, within the attractive components, the greater contribution to the stabilisation of the binding interaction comes from the electrostatic interactions (ca. 46 % in **XB1_Et_**; ca. 60 % in **XB2_Et_**), followed by the induction forces (ca. 36 % in **XB1_Et_**; ca. 28 % in **XB2_Et_**), and by the dispersive contributions (ca. 19 % in **XB1_Et_**; ca. 12 % in **XB2_Et_**).

Overall, the recognition of HS^−^ and I^−^ by the model receptors is accompanied by a loss of electron density around the guest anion and a gain of electron density along the iodotriazole binding units. This is illustrated in Figures 4 and S9‐4, where the changes in electron density between the associations with HS^−^ or I^−^ and their individual components are plotted (loss of electron density in blue and gains in pink), being more prominent in the charged **XB2_Et_**.

**Figure 4:**
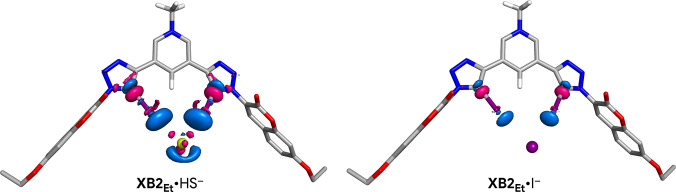
Electron density difference maps for the **XB2_Et_** associations with HS^−^ and I^−^ highlighting the superior electron transfer in the HS^−^ host–guest association.

The XB‐based associations between HS^−^ and I^−^ and the complete receptors were also investigated by molecular dynamics (MD) simulations in water, carried out under periodic boundary conditions, using classical force field parameters and an extra‐point of charge to represent the iodine binding units’ σ‐holes (see SI for remaining simulation details). Regardless of the anion–receptor association, the XB interactions are maintained nearly throughout the whole simulation time (see Table S9‐6 for their average dimensions). In addition, the halogen‐bonded HS^−^ and I^−^ anions are surrounded by water molecules as shown for their associations with **XB2** in Figure 5 and in Figure S9‐5 for their associations with neutral **XB1**. Furthermore, the TEG chains exhibited a large conformational flexibility along the MD runs, indicating that they have no role in the shielding of the guest anions from the solvating water molecules. A small decrease on the average number of the anions’ surrounding water molecules is observed when compared with the freely solvated anions, from 10.6 to 7.9 for HS^−^ and from 7.7 to 6.0 for I^−^ for an anion–water distance cut‐off of 3.5 Å (see Table S9‐7 and Figure S9‐6). This result suggests that the water molecules play an important role in the recognition of both anions. A deeper understanding of this matter might be obtained from QM/MM MD simulations in water with the XB associations, along with fewer water molecules surrounding the anions (typically the first solvation sphere), treated by QM methods. However, this robust and computationally demanding approach falls beyond of the scope of this communication.

**Figure 5:**
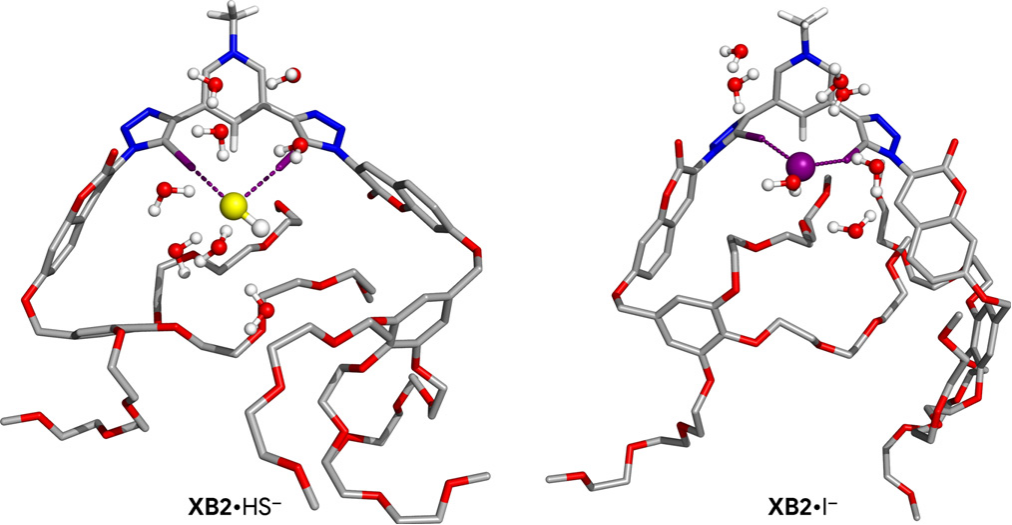
A snapshot of a MD simulation of HS^−^ and I^−^ associated with **XB2**, showing the anions enclosed by water molecules and the random orientation of the TEG flexible chains. Most hydrogen atoms are hidden for clarity.

In conclusion, XB anion recognition and reversible chemosensing of HS^−^ in pure water has been demonstrated for the first time. This was achieved through the integration of coumarin fluorophores into XB bis‐iodotriazole pyridine, pyridinium and secondary amine acyclic host structural frameworks, functionalised with hydrophilic triethylene glycol (TEG) substituents. Importantly, **XB1** exhibits exclusive chemosensing behaviour for HS^−^, exhibiting strong and selective recognition of HS^−^ over halide anions, which are not bound under aqueous buffered conditions. Selective binding of HS^−^ was also displayed by **XB2**. In stark contrast, the HB receptor analogues were incapable of functioning as chemosensors for HS^−^ under these competitive aqueous conditions. These results, corroborated by computational methods, further advance and highlight the potential of halogen bonding‐based sensor systems for detecting anions in aqueous media.

## Conflict of interest

The authors declare no conflict of interest.

## Supporting information

As a service to our authors and readers, this journal provides supporting information supplied by the authors. Such materials are peer reviewed and may be re‐organized for online delivery, but are not copy‐edited or typeset. Technical support issues arising from supporting information (other than missing files) should be addressed to the authors.

Supporting Information
