# Supplementary material for: Hydrosulfide (HS−) Recognition and Sensing in Water by Halogen Bonding Hosts
Source: Angew Chem Int Ed Engl. 2021 Oct 5;60(45):24048–53. doi: 10.1002/anie.202110442 (PMC8596634; doi:10.1002/anie.202110442)
Supplement: Supplementary file 1 — Supporting Information [file ANIE-60-24048-s001.pdf]

Angewandte Chemie
Eine Zeitschrift der Gesellschaft Deutscher Chemiker

Supporting Information

Hydrosulfide (HS^{-}) Recognition and Sensing in Water by Halogen Bonding Hosts

*Edward J. Mitchell, Adam J. Beecroft, Jonathan Martin, Sally Thompson, Igor Marques, Vítor Félix, and Paul D. Beer**

anie_202110442_sm_miscellaneous_information.pdf

Table of Contents

S1. Synthesis of Compounds	S3
S2. Spectral Characterisation of Compounds	S14
S3. Fluorescence Titrations	S27
S4. Reversibility Studies	S34
S5. pKa Experiments	S35
S6. MS Experiments	S36
S7. Limit of Detection	S37
S8. Synthetic and Titration References	S38
S9. Molecular Modelling	S39
S10. Molecular Modelling References	S48

S1. Synthesis of Compounds

S1.1 General Information

All commercially available chemicals and solvents were used as received without further purification. All dry solvents were thoroughly degassed with N_2, dried through a Mbraun MPSP-800 column and used immediately. Water used was deionized and passed through a Milli-Q ® Millipore machine for microfiltration. TBTA (tris(benzyltriazolemethyl)amine) was prepared according to reported procedures.[1] Chromatography was undertaken using silica gel (particle size: 40-63 µm) or preparative TLC plates (20 × 20 cm, 1 cm silica thickness).

NMR spectra were recorded on Bruker AVIII HD Nanobay 400 MHz, Bruker AVIII 500 MHz and Bruker AVIII 500 MHz spectrometers. Low resolution electrospray ionisation mass spectrometry (ESI-MS) was performed using the Waters Micromass LCT for characterisation of compounds previously reported in the literature, and high resolution ESI-MS was recorded using Bruker microTOF spectrometer for novel compounds.

S1.2 Synthesis receptor XB1

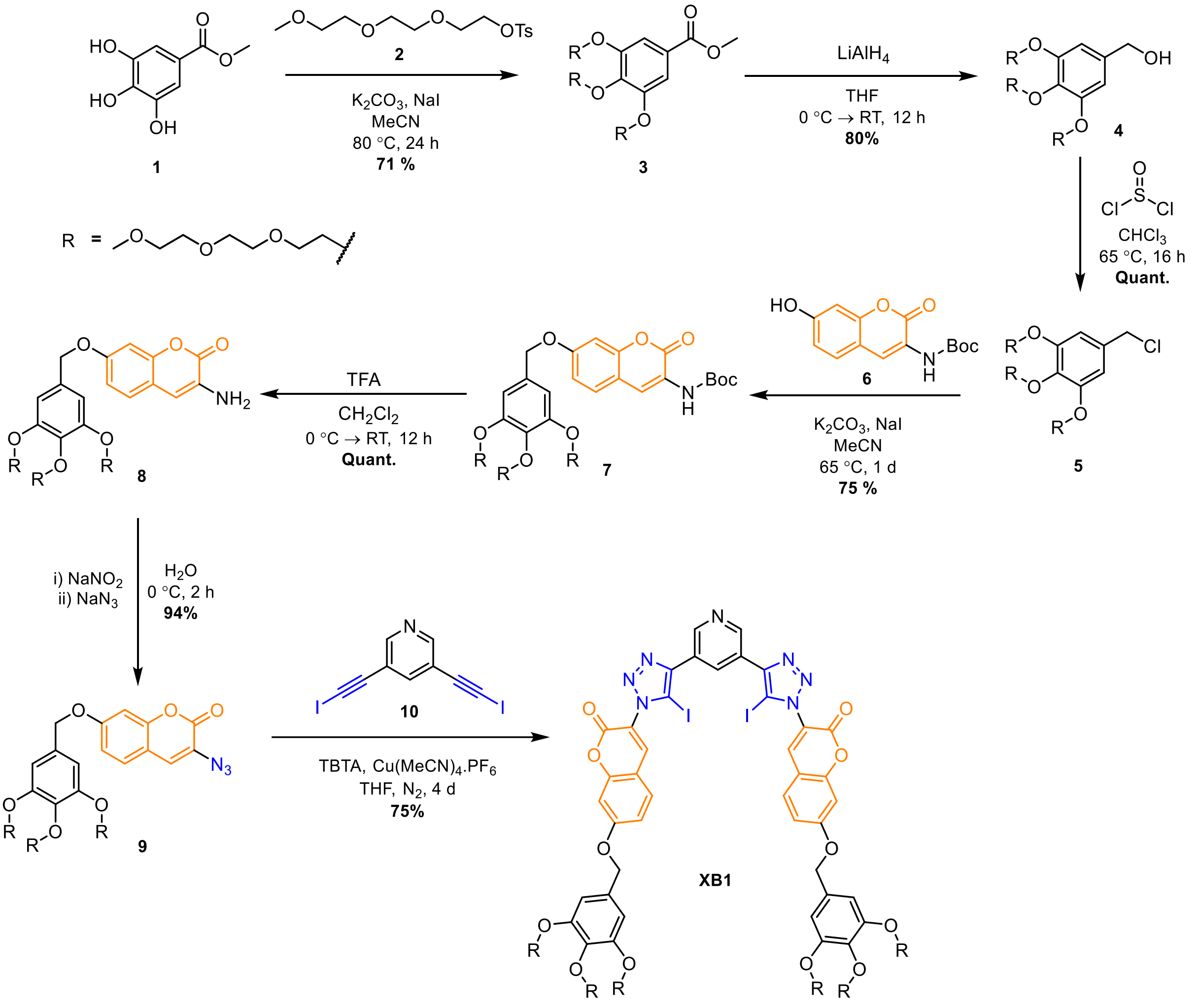

TEG groups **2-5**[2], coumarin **6**[3] and bis-alkyne **10**[4] were prepared as reported.

3-Teg *N*-boc-protected coumarin **7**

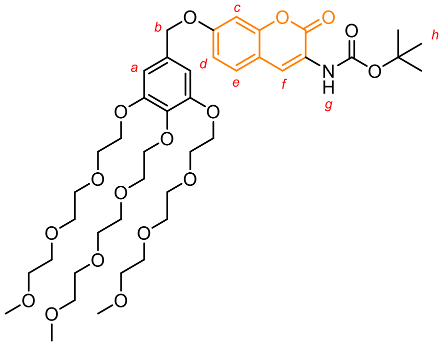

Boc-protected coumarin derivative **6** (369 mg, 1.33 mmol, 1.2 eq), PEG chloride **5** (678 mg, 1.11 mmol, 1 eq) K_2CO_3 (368 mg, 2.66 mmol, 2.4 eq) and NaI (17 mg, 0.11 mmol, 0.1 eq) were dissolved in MeCN (15 mL) and heated to 65 °C for 24 hours. The solvent was then removed *in vacuo*, the crude dissolved in H_2O (40 mL) and the product extracted with CH_2Cl_2 (3 x 40 mL) washes. The organic layers were combined and washed with brine (50 mL), dried over $MgSO_4$, filtered and the solvent removed *in vacuo.* The crude product was purified *via* column chromatography (eluent: EtOAc/CH_2Cl_2 1:1 *v/v* followed by MeOH/CH_2Cl_2 4:96 *v/v*) and the desired product was isolated as a yellow oil (707 mg, 75%).

^{1}H NMR (500 MHz, $CDCl_3$) δ 8.24 (s, 1H, H_g), 7.36 (d, *J* = 8.6 Hz, 1H, H_e), 7.29 (s, 1H, H_f), 6.92 (dd, *J* = 8.6, 2.4 Hz, 1H, H_d), 6.87 (d, *J* = 2.4 Hz, 1H, H_c), 6.66 (s, 2H, H_a), 4.98 (s, 2H, H_b), 4.17 – 4.12 (m, 6H, OCH_2), 3.86 – 3.83 (m, 4H, OCH_2), 3.78 – 3.77 (m, 2H, OCH_2), 3.73 – 3.70 (m, 6H, OCH_2), 3.68 – 3.60 (m, 12H, OCH_2), 3.55 – 3.52 (m, 6H, OCH_2), 3.37 (s, 3H, OCH_3), 3.36 (s, 6H, OCH_3), 1.52 (s, 9H, H_h); **^{13}C NMR** (126 MHz, $CDCl_3$) δ 159.69, 158.86, 152.90, 152.60, 150.84, 138.41, 131.36, 128.15, 122.29, 121.17, 113.67, 113.61, 107.20, 101.75, 72.34, 71.95, 71.93, 70.82, 70.69, 70.56, 70.53, 70.51, 69.72, 68.92, 59.03, 53.43, 28.23; **HRMS** (ESI +ve) m/z=853.416 ($[M + H]^+$, $C_{42}H_{63}NO_{17}$, calc=854.415)

3-Peg coumarin amine **8**

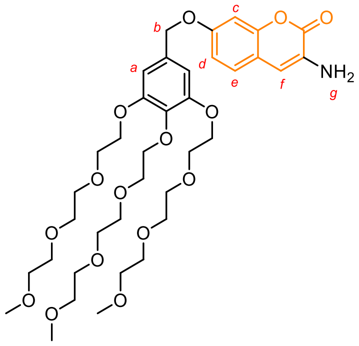

3-Peg *N*-boc-protected coumarin **7** (707 mg, 0.828 mmol, 1 eq) was dissolved in CH_2Cl_2 and cooled to 0 °C in an ice bath. CF_3CO_2H (0.5 mL, excess) was slowly added dropwise and the reaction left to stir and warm up to rt over 2 hours. The solution was quenched with saturated aqueous $NaHCO_3$ (50 mL) solution and extracted with CH_2Cl_2 (5 x 50 mL). The combined organic layers were dried over Mg_2SO_4, filtered and the solvent removed *in vacuo* to obtain the desired product (619 mg, 99%) as a yellow oil with no need for further purification.

^{1}H NMR (400 MHz, $CDCl_3$) δ 7.19 (d, 1H, 9.07 Hz, H_e), 6.88 – 6.84 (m, 2H, $H_{c,d}$), 6.69 (s, 1H, H_f), 6.65 (s, 2H, H_a), 4.96 (s, 2H, H_b), 4.16 – 4.14 (m, 6H, OCH_2), 4.07 (s, 2H, H_g), 3.85 – 3.83 (m, 4H, OCH_2), 3.80 – 3.78 (m, 2H, OCH_2), 3.75 – 3.68 (m, 6H, OCH_2), 3.69 – 3.60 (m, 12H, OCH_2), 3.57 – 3.50 (m, 6H,

OCH_2), 3.37 (s, 4H, OCH_3), 3.36 (s, 6H, OCH_3); **^{13}C NMR** (126 MHz, $CDCl_3$) δ 159.68, 157.96, 152.85, 150.20, 138.31, 131.68, 129.88, 125.85, 114.72, 113.23, 112.04, 107.16, 101.87, 72.33, 71.95, 71.93, 70.81, 70.68, 70.55, 70.53, 70.50, 70.48, 69.72, 68.90, 59.02; **HRMS** (ESI +ve) m/z=776.34639 ([M + Na]$^+$, $C_{37}H_{55}NO_{15}$, calc=776.34583)

3-Peg coumarin azide **9**

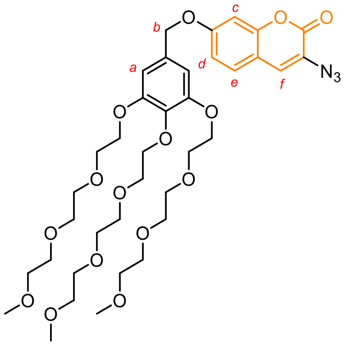

3-Peg coumarin amine **9** (376 mg, 0.50 mmol, 1 eq) was dissolved in 15% $HCl_{(aq)}$ (8 mL) and cooled to 0 °C in an ice bath. $NaNO_2$ (41.4 mg, 0.60 mmol, 1.2 eq) was added slowly and the solution left to stir for 30 minutes. NaN_3 (52 mg, 30.80 mmol, 1.6 eq) in H_2O (2 mL) was slowly added dropwise and the solution left to stir at 0°C for 1 hour. The reaction was neutralised with a saturated aqueous solution of $NaHCO_3$ and the product extracted with EtOAc (5 x 30 mL). The organic layers were combined, dried over $MgSO_4$ and the solvent removed *in vacuo* to yield the desired product (366 mg, 94%) as an orange oil.

^{1}H NMR (400 MHz, $CDCl_3$) δ 7.25 (d, J = 8.6 Hz, 1H, H_e), 7.10 (s, 1H, H_f), 6.85 (dd, J = 8.6, 2.4 Hz, 1H, H_d), 6.82 (d, J = 2.4 Hz, 1H, H_d), 6.58 (s, 2H, H_a), 4.93 (s, 2H, H_b), 4.09 (m, 6H, OCH_2), 3.78 (m, 4H, OCH_2), 3.72 (m, 2H, OCH_2), 3.65 (m, 6H, OCH_2), 3.58 (m, 12H, OCH_2), 3.47 (m, 6H), 3.30 (s, 3H, OCH_3), 3.30 (s, 6H, OCH_3); **^{13}C NMR** (126 MHz, CDCl3) δ 160.71, 157.67, 152.94, 152.91, 138.54, 131.07, 128.16, 126.30, 123.50, 113.90, 112.90, 107.24, 101.79, 72.34, 71.96, 71.94, 70.82, 70.70, 70.61, 70.56, 70.54, 70.52, 69.72, 68.96, 59.03; **HRMS** (ESI +ve) m/z=802.33689 ([M + Na]$^+$, $C_{37}H_{53}N_3O_{15}$, calc=802.33646)

Host receptor **XB1**

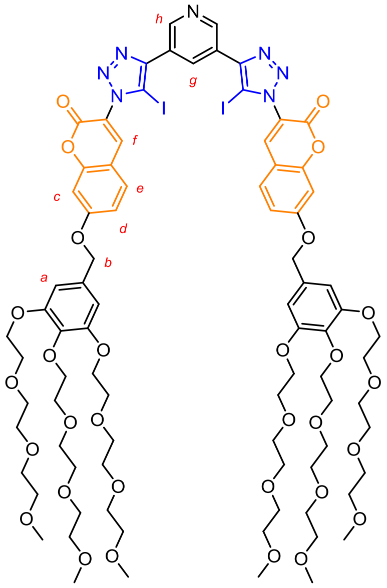

3-Peg coumarin azide **9** (95.0 mg, 0.12 mmol, 2.2 eq), bis-iodo-alkyne **13** (21.0 mg, 0.055 mmol, 1 eq), TBTA (5.8 mg, 0.011 mmol, 0.2 eq), and $Cu(CH_3CN)_4.PF_6$ (4.1 mg, 0.06 mmol, 0.2 eq) were dissolved in dry degassed THF (0.8 mL) and left to stir at room temperature under N_2 for 3 days. The solvent was removed *in vacuo*, the crude dissolved in CH_2Cl_2 (15 mL) and washed with EDTA solution (3 x 15 mL). The combined organic phases were dried over $MgSO_4$, filtered and the solvent removed *in vacuo*. Purification was achieved *via* preparative thin layer chromatography (eluent: MeOH/CH_2Cl_2 1:19 *v/v*) affording the desired product (71 mg, 66%) as a viscous yellow oil.

^{1}H NMR (500 MHz, $CDCl_3$) δ 9.39 (d, *J* = 2.1 Hz, 2H, H_h), 9.02 (d, *J* = 2.1 Hz, 1H, H_g), 8.10 (s, 2H, H_f), 7.60 (d, *J* = 8.7 Hz, 2H,H_e), 7.08 (dd, *J* = 8.7, 2.4 Hz, 2H, H_d), 7.03 (d, *J* = 2.4 Hz, 2H, H_c), 6.71 (s, 4H, H_a), 5.11 (s, 4H, H_b), 4.22 – 4.17 (m, 12H, OCH_2), 3.90 – 3.87 (m, 8H, OCH_2), 3.83 – 3.81 (m, 4H, OCH_2), 3.77- 3.74 (m, 12H, OCH_2), 3.71 – 3.66 (m, 24H, OCH_2), 3.58 – 3.56 (m, 12H, OCH_2), 3.40 – 3.39 (m, 18H, OCH_3); **^{13}C NMR** (126 MHz, $CDCl_3$) δ 163.75, 156.38, 156.24, 153.01, 148.36, 147.45, 142.79, 138.63, 132.87, 130.59, 130.49, 125.90, 120.63, 114.70, 111.31, 107.26, 102.17, 72.36, 71.95, 71.93, 70.94, 70.82, 70.69, 70.56, 70.54, 70.52, 69.71, 68.96, 59.05; **Fluorescence** (H_2O, 10 mM HEPES): λ_{ex}= 344 nm; λ_{em}= 414 nm; **HRMS** (ESI +ve) m/z= 1938.53697 ($[M + H]^+$, $C_{83}H_{110}N_7O_{30}I_2$, calc= 1938.53810)

S1.3 Synthesis receptor HB1

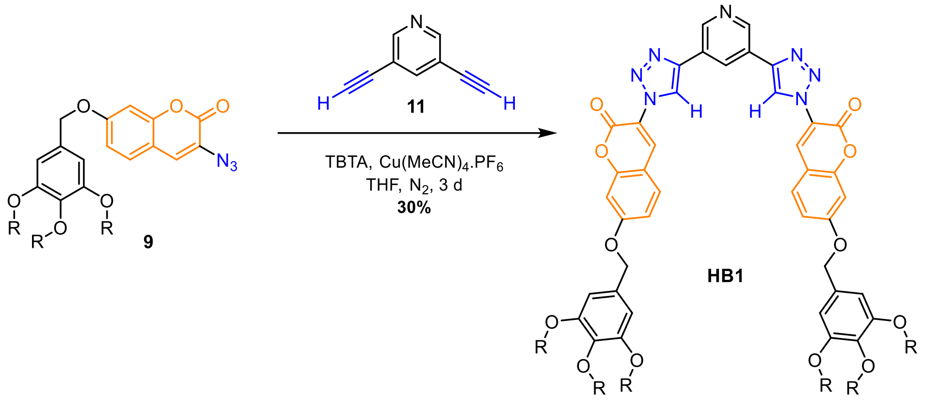

Bis-alkyne **11**[5] was prepared as reported.

<u>Host receptor **HB1**</u>

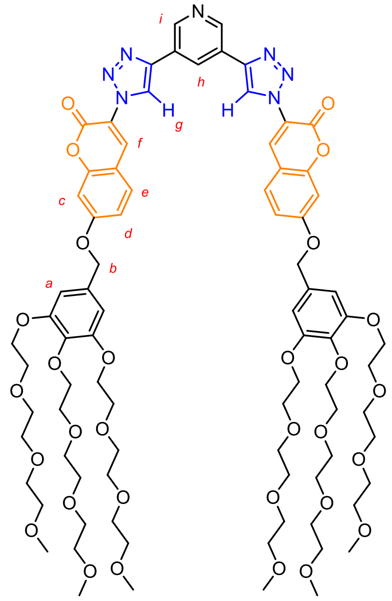

3-Peg coumarin azide **9** (348 mg, 0.47 mmol, 2.2 eq), bis-alkyne **11** (25.8 mg, 0.203 mmol, 1 eq), TBTA (21.5 mg, 0.04 mmol, 0.2 eq), and $Cu(CH_3CN)_4.PF_6$ (15.1 mg, 0.04 mmol, 0.2 eq) were dissolved in dry degassed THF (3 mL) and left to stir at room temperature under N_2 for 3 days. The solvent was removed *in vacuo*, the crude dissolved in CH_2Cl_2 (50 mL) and washed with EDTA solution (3 x 40 mL) and brine (50 mL). The combined organic phases were dried over $MgSO_4$, filtered and the solvent removed *in vacuo*. Purification was achieved *via* column chromatography (eluent: MeOH/CH_2Cl_2 1:19 *v/v*) affording the desired product (104 mg, 30%) as a viscous yellow oil.

^{1}**H NMR** (500 MHz, $CDCl_3$) δ 9.17 (d, J = 2.1 Hz, 2H, H_i), 9.06 (s, 2H, H_g), 8.76 (t, J = 2.2 Hz, 1H, H_h), 8.64 (s, 2H, H_f), 7.63 (d, J = 8.8 Hz, 2H, H_e), 7.06 (dd, J = 8.8, 2.4 Hz, 2H, H_d), 6.99 (d, J = 2.4 Hz, 2H, H_c), 6.68 (s, 4H, H_a), 5.07 (s, 2H, H_b), 4.19 – 4.16 (m, 12H, OCH_2), 3.89 – 3.85 (m, 8H, OCH_2), 3.81 – 3.79 (m, 4H, OCH_2), 3.76 – 3.70 (m, 12H, OCH_2), 3.70 – 3.62 (m, 24H, OCH_2), 3.56 – 3.54 (m, 12H, OCH_2), 3.38 – 3.37 (m, 18H, OCH_3); ^{13}**C NMR** (126 MHz, $CDCl_3$) δ 163.00, 156.27, 154.76, 152.95, 146.44, 144.49, 138.40, 134.27, 130.95, 130.41, 130.29, 126.72, 121.53, 120.16, 114.87, 111.80, 107.18, 101.89, 72.38, 71.94, 71.92, 70.89, 70.80, 70.65, 70.57, 70.51, 70.49, 69.73, 68.89, 59.02, 50.56; **Fluorescence** (H_2O, 10 mM HEPES): λ_{ex}= 342 nm; λ_{em}= 498 nm; **HRMS** (High resolution ESI +ve) m/z= 1686.7401 ($[M + H]^+$, $C_{83}H_{111}N_7O_{30}$, calc= 1686.7448)

S1.4 Synthesis receptor XB2

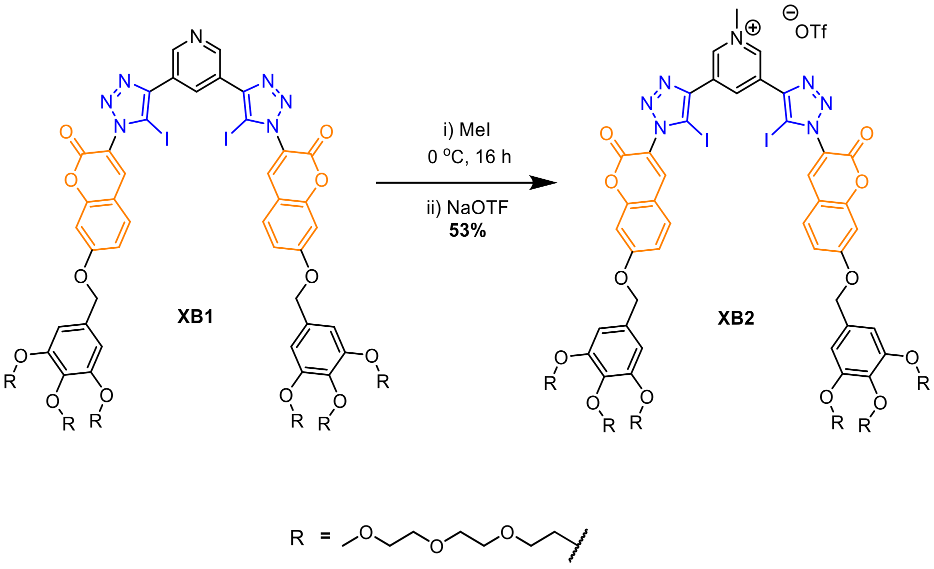

Host receptor **XB2**

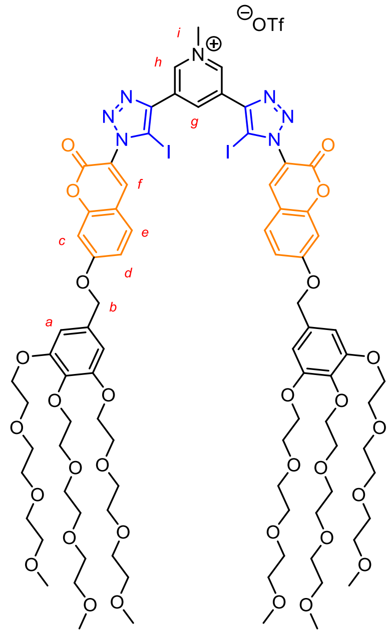

Receptor **XB1** (20.2 mg, 0.01 mmol, 1 eq) was dissolved in dry CH_2Cl_2 (0.1 mL) and cooled to 0 °C. MeI (0.2 mL) was added and the reaction left to stir for 16 h under N_2. The solvent was removed *in vacuo* and anion exchange to the triflate salt was achieved by passing through an Amberlite (triflate) column (11.6 mg, 53%).

^{1}H NMR (400 MHz, $CDCl_3$) δ 9.59 (s, 3H, $H_{h,g}$), 8.11 (s, 2H, H_f), 7.59 (d, J = 8.8 Hz, 2H, H_e), 7.06 (dd, J = 8.8, 2.3 Hz, 2H, H_d), 6.92 (d, J = 2.3 Hz, 2H, H_c), 6.61 (s, 4H, H_a), 5.04 (s, 4H, H_b), 4.76 (s, 3H, H_i), 4.13 – 4.04 (m, 12H, OCH_2), 3.83 – 3.78 (m, 8H, OCH_2), 3.78 – 3.65 (m, 32H, OCH_2), 3.64 – 3.59 (m, 8H, OCH_2), 3.59 – 3.54 (m, 4H, OCH_2), 3.54 – 3.48 (m, 8H), 3.33 (s, 6H, OCH_3), 3.30 (s, 12H, OCH_3); **^{13}C NMR** (126 MHz, $CDCl_3$) δ 164.02, 156.70, 156.25, 152.41, 143.49, 142.97, 142.64, 137.68, 133.17, 130.95, 130.35, 129.30, 128.22, 121.75, 119.87, 119.20, 114.80, 111.19, 106.57, 102.14, 86.11, 72.33, 71.97, 71.92, 70.93, 70.84, 70.71, 70.69, 70.49, 69.75, 68.57, 58.96, 58.92, 53.80, 49.40; **^{19}F NMR** (377 MHz, $CDCl_3$) δ -77.86; **Fluorescence** (H_2O, 10 mM HEPES): λ_{ex}= 344 nm; λ_{em}= 408 nm; **HRMS** (ESI +ve) m/z= 1952.5531 ($[M]^+$, $C_{84}H_{112}I_2N_7O_{30}$+, calc= 1952.5537)

S1.5 Synthesis receptor HB2

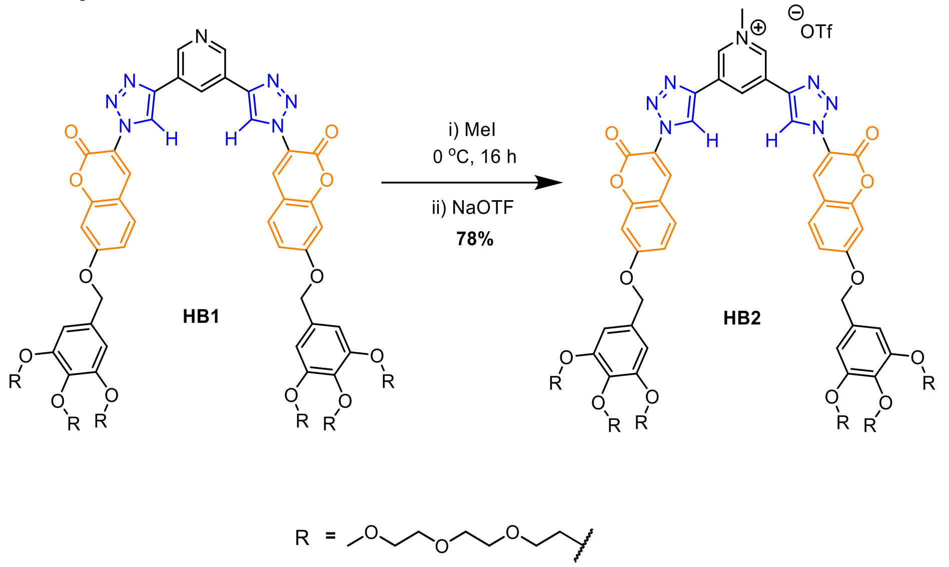

Host receptor **HB2**

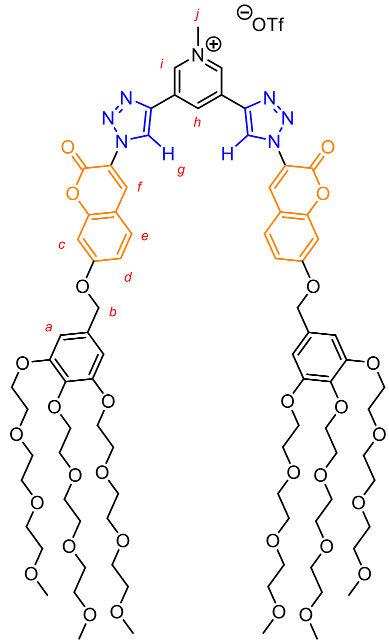

Receptor **HB1** (9 mg, 5.34 µmol, 1 eq) was dissolved in dry CH_2Cl_2 (0.5 mL) and cooled to 0 °C. MeI (0.5 mL) was added and the reaction left to stir for 16 h under N_2. The solvent was removed *in vacuo* and anion exchange to the triflate salt was achieved by passing through an Amberlite (triflate) column (7.7 mg, 78%).

^{1}H NMR (500 MHz, $CDCl_3$) δ 9.43 (s, 2H, H_h), 9.26 (s, 2H, H_g), 9.25 (s, 1H, H_h), 8.44 (s, 2H, H_f), 7.59 (d, J = 8.8 Hz, 2H, H_e), 7.09 (dd, J = 8.8, 2.4 Hz, 2H, H_d), 6.85 (d, J = 2.4 Hz, 2H, H_c), 6.55 (s, 4H, H_a), 4.91 (s, 4H, H_b), 4.68 (s, 3H, H_j), 4.17 – 4.13 (m, 8H, OCH_2), 4.01 – 3.98 (m, 4H, OCH_2), 3.83 – 3.80 (m, 8H, OCH_2), 3.77 – 3.70 (m, 22H, OCH_2), 3.69 – 3.67 (m, 7H, OCH_2), 3.65 – 3.61 (m, 8H, OCH_2), 3.60 – 3.56 (m, 3H, OCH_2), 3.52 – 3.48 (m, 8H, OCH_2), 3.29 (s, 11H, OCH_3), 3.28 (s, 6H, OCH_3); **^{13}C NMR** (126 MHz, $CDCl_3$) δ 162.86, 155.95, 154.75, 152.35, 141.35, 141.05, 137.67, 135.48, 134.74, 130.81, 130.62, 130.57, 124.32, 122.14, 119.91, 119.59, 114.70, 111.49, 105.85, 101.43, 72.14, 71.95, 71.90, 70.91, 70.76, 70.71, 70.68, 70.58, 70.53, 70.50, 70.34, 69.73, 68.43, 58.93, 58.81, 48.72; **Fluorescence** (H_2O, 10 mM HEPES): λ_{ex}= 354 nm; λ_{em}= 426 nm; **HRMS** (ESI +ve) m/z= 1700.7563 ($[M]^+$, $C_{84}H_{114}N_7O_{30}^+$, calc= 1700.7605)

S1.6 Synthesis receptor XB3

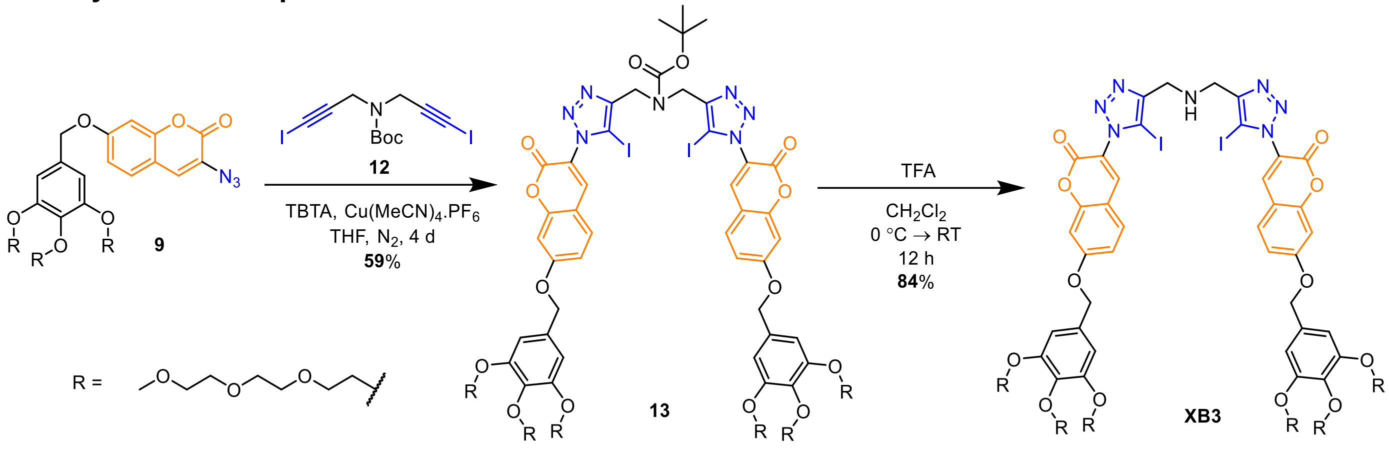

Bis-alkyne **12**[6] was prepared as reported.

<u>*N*-boc-protected host receptor **13**</u>

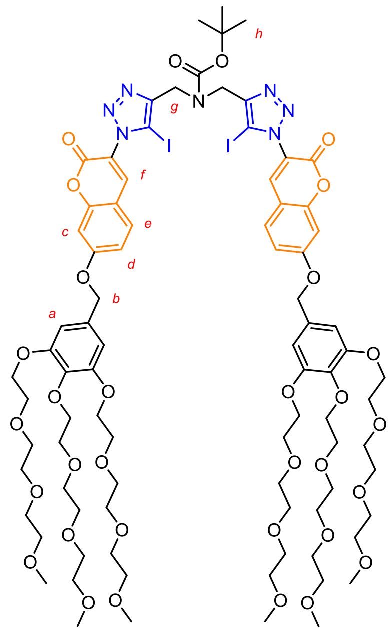

3-Peg coumarin azide **9** (80.0 mg, 0.10 mmol, 2.2 eq), bis-iodo-alkyne **12** (20.8 mg, 0.047 mmol, 1 eq), TBTA (5 mg, 0.0094 mmol, 0.2 eq), and $Cu(CH_3CN)_4.PF_6$ (5.3 mg, 0.014 mmol, 0.3 eq) dissolved in 1.4 ml dry degassed THF, left to stir at room temperature under N_2 for 3 days. Solvent removed on rotary evaporator then product dissolved in CH_2Cl_2. Washed 3 times with EDTA solution, extracting all washes multiple times with $CHCl_3$. Organic phases combined and dried over $MgSO_4$, solvent removed in vacuo. Purification *via* preparative thin layer chromatography using 90% DCM 10% MeOH yielded the product (55 mg, 59%) as a viscous yellow oil.

^{1}H NMR (500 MHz, $CDCl_3$) δ 8.00 (s, 2H, H_f), 7.55 (d, J = 8.7 Hz, 2H, H_e), 7.03 (dd, J = 8.6, 2.4 Hz, 2H, H_d), 6.98 (d, J = 2.4 Hz, 2H, H_c), 6.67 (s, 4H, H_a), 5.07 (s, 4H, H_b), 4.77 (d, J = 40.2 Hz, 4H, H_g), 4.19 – 4.16 (m, 12H, OCH_2), 3.87 – 3.85 (m, 8H, OCH_2), 3.81 – 3.79 (m, 4H, OCH_2), 3.75 – 3.72 (m, 12H, OCH_2), 3.67 – 3.63 (m, 24H, OCH_2), 3.56 – 3.53 (m, 12H, OCH_2), 3.37 (s, 6H, OCH_3), 3.37 (s, 12H, OCH_3), 1.51 (s, 9H, H_h); **^{13}C NMR** (176 MHz, $CDCl_3$) δ 163.55, 156.36, 156.12, 155.17, 153.00, 148.69, 142.47, 138.66, 130.64, 130.42, 120.77, 114.51, 111.39, 107.30, 102.07, 80.87, 72.37, 71.96, 71.94, 70.91, 70.82, 70.69, 70.56, 70.54, 70.52, 69.71, 68.98, 59.04, 28.53; **HRMS** (ESI +ve) m/z=2004.60618 ($[M + H]^+$, $C_{85}H_{120}I_2N_7O_{32}$, calc=2004.60226)

Host receptor **XB3**

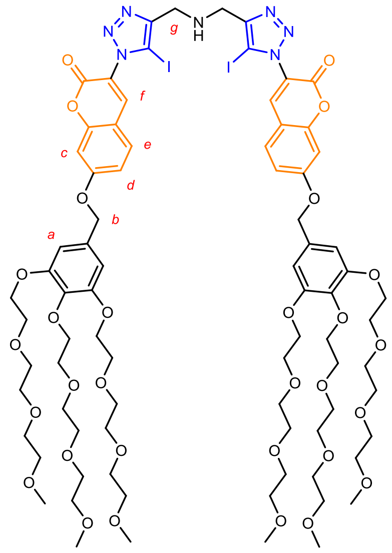

N-boc-protected receptor **13** (50.0 mg, 0.025 mmol, 1 eq) was dissolved in CH_2Cl_2 (0.3 mL) and cooled to 0°C. TFA (0.5 mL, excess) was added and the reaction left to stir to room temperature over 3 h. Saturated aqueous $NaHCO_3$ solution was added till neutral and the product extracted with CH_2Cl_2 (5 x 10 mL). The combined organic layers were dried over Na_2SO_4, filtered and the solvent removed *in vacuo* to afford the desired product (40.0 mg, 84 %) as a viscous yellow oil.

^{1}H NMR (500 MHz, $CDCl_3$) δ 7.98 (s, 2H, H_f), 7.54 (d, J = 8.7 Hz, 2H, H_e), 7.03 (dd, J = 8.7, 2.4 Hz, 2H, H_d), 6.98 (d, J = 2.4 Hz, 2H, H_c), 6.67 (s, 4H, H_a), 5.07 (s, 4H, H_b), 4.20 – 4.14 (m, 12H, OCH_2), 4.04 (s, 4H, H_g), 3.86 – 3.84 (m, 8H, OCH_2), 3.81 – 3.78 (m, 4H, OCH_2), 3.74 – 3.71 (m, 12H, OCH_2), 3.68 – 3.62 (m, 24H, OCH_2), 3.56 – 3.52 (m, 12H, OCH_2), 3.37 (s, 6H, OCH_3), 3.37 (s, 12H, OCH_3); **^{13}C NMR** (126 MHz, $CDCl_3$) δ 163.56, 156.36, 156.11, 153.01, 150.35, 142.31, 138.64, 130.62, 130.38, 120.77, 114.55, 111.37, 107.28, 102.09, 82.24, 72.37, 71.96, 71.94, 70.92, 70.83, 70.70, 70.57, 70.55, 70.53, 69.72, 68.97, 59.05, 44.10, 29.74; **Fluorescence** (H_2O, 10 mM HEPES): λ_{ex}= 342 nm; λ_{em}= 412 nm; **HRMS** (ESI +ve) m/z=1904.55375 ([M + H]$^+$, $C_{80}H_{112}I_2N_7O_{30}$, calc=1904.54083)

S1.7 Synthesis receptor HB3

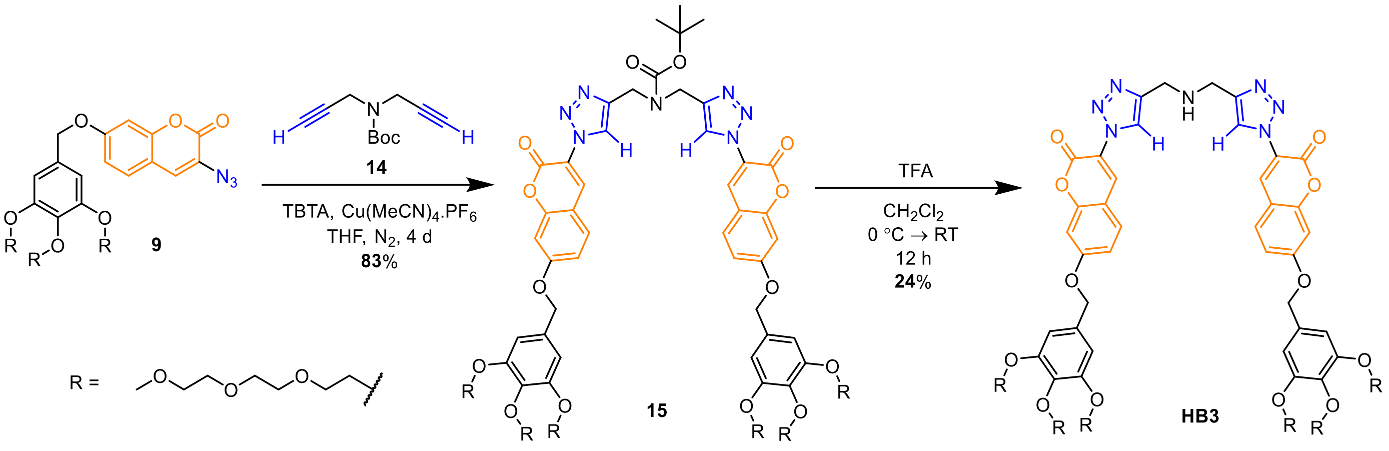

Bis-alkyne **14**[6] was prepared as reported.

<u>*N*-boc-protected host receptor **15**</u>

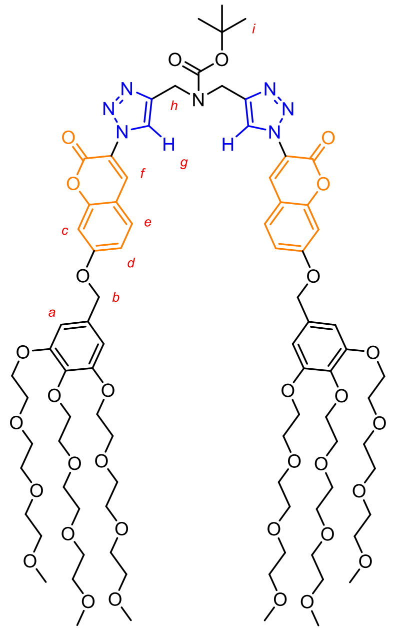

3-Peg coumarin azide **9** (40.0 mg, 0.05 mmol, 2.2 eq), bis-iodo-alkyne **14** (4.5 mg, 0.023 mmol, 1 eq), TBTA (2.5 mg, 0.005 mmol, 0.2 eq), and $Cu(CH_3CN)_4.PF_6$ (2.6 mg, 0.007 mmol, 0.3 eq) were dissolved in dry degassed THF (1.4 mL) and left to stir at room temperature under N_2 for 3 days. The solvent was removed *in vacuo*, the crude dissolved in CH_2Cl_2 (10 mL) and washed with EDTA solution (3 x 10 mL). The combined organic phases were dried over $MgSO_4$, filtered and the solvent removed *in vacuo*. Purification was achieved *via* preparative thin layer chromatography (eluent: MeOH/CH_2Cl_2 1:19 *v/v*) affording the desired product (34 mg, 83%) as a viscous yellow oil.

^{1}H NMR (500 MHz, $CDCl_3$) δ 8.60 – 8.48 (m, 4H, $H_{f,g}$), 7.60 (d, *J* = 8.7 Hz, 2H, H_e), 7.06 (dd, *J* = 8.7, 2.4 Hz, 2H, H_d), 6.99 (d, *J* = 2.4 Hz, 2H, H_c), 6.70 (s, 4H, H_a), 5.08 (s, 4H, H_b), 4.73 (d, *J* = 24.4 Hz, 4H, H_h), 4.19 – 4.13 (m, 12H, OCH_2), 3.87– 3.84 (m, 8H, OCH_2), 3.81 – 3.78 (m, 4H, OCH_2), 3.74 – 3.71 (m, 12H, OCH_2), 3.67 – 3.63 (m, 24H, OCH_2), 3.56 – 3.53 (m, 12H, OCH_2), 3.37 (s, 6H, OCH_3), 3.37 (s, 12H, OCH_3), 1.54 (s, 9H, H_i); **^{13}C NMR** (126 MHz, $CDCl_3$) δ 162.65, 156.10, 155.20, 154.59, 153.00, 138.62, 133.80, 130.77, 130.00, 120.54, 114.56, 111.84, 107.29, 101.78, 80.92, 72.37, 71.97, 71.94, 70.83, 70.71, 70.58, 70.55, 70.53, 69.73, 68.97, 59.05, 53.44, 42.28, 41.82, 28.46; **HRMS** (ESI +ve) m/z=1774.79484 ([M + Na]$^+$, $C_{85}H_{121}N_7O_{32}$, calc=1774.79556)

Host receptor **HB1**

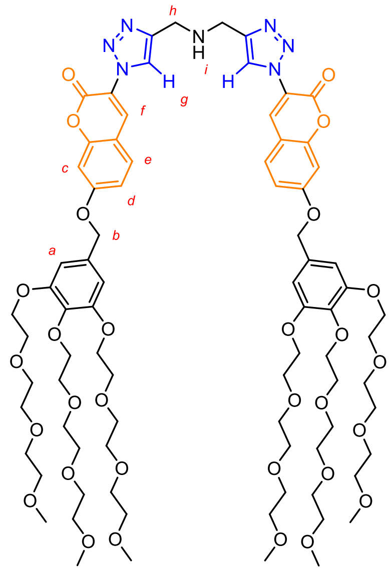

N-boc-protected receptor **12** (17 mg, 0.01 mmol, 1 eq) was dissolved in CH_2Cl_2 (0.2 mL) and cooled to 0°C. TFA (0.4 mL, excess) was added and the reaction left to stir to room temperature over 5 h. Saturated aqueous $NaHCO_3$ solution was added till neutral and the product extracted with CH_2Cl_2 (5 x 10 mL). The combined organic layers were dried over $MgSO_4$, filtered and the solvent removed *in vacuo* to afford the desired product (4 mg, 24 %) as a viscous yellow oil.

^{1}H NMR (500 MHz, $CDCl_3$) δ 8.58 (d, 2H, H_g), 8.51 (d, 2H, H_f), 7.57 (d, *J* = 8.7 Hz, 2H, H_e), 7.03 (dd, *J* = 8.7, 2.4 Hz, 2H, H_d), 6.96 (d, *J* = 2.4 Hz, 2H, H_c), 6.67 (s, 4H, H_a), 5.05 (s, 4H, H_b), 4.19 – 4.11 (m, 12H, OCH_2), 3.87 – 3.82 (m, 8H, OCH_2), 3.81 – 3.77 (m, 4H, OCH_2), 3.74 – 3.70 (m, 12H, OCH_2), 3.67 – 3.62 (m, 24H, OCH_2), 3.55 – 3.52 (m, 12H, OCH_2), 3.49 (s, 4H, H_h), 3.37 – 3.36 (m, 18H, OCH_3); **^{13}C NMR** (126 MHz, $CDCl_3$) δ 162.67, 156.13, 154.60, 152.99, 152.72, 145.88, 138.59, 137.83, 136.65, 133.79, 130.77, 130.00, 123.22, 120.51, 114.59, 111.83, 107.27, 106.68, 101.78, 72.36, 72.27, 71.96, 71.94, 70.83, 70.78, 70.72, 70.70, 70.57, 70.55, 70.52, 69.83, 69.72, 68.96, 68.86, 65.33, 59.05, 43.66, 31.94, 29.71, 22.71, 14.14, 1.03; **Fluorescence** (H_2O, 10 mM HEPES): λ_{ex}= 342 nm; λ_{em}= 484 nm; **HRMS** (High resolution ESI +ve) m/z=1652.76046 ([M + H]$^+$, $C_{80}H_{114}N_7O_{30}$, calc=1652.76113)

S2. Spectral Characterisation of Compounds

S2.1. Host receptor **XB1**

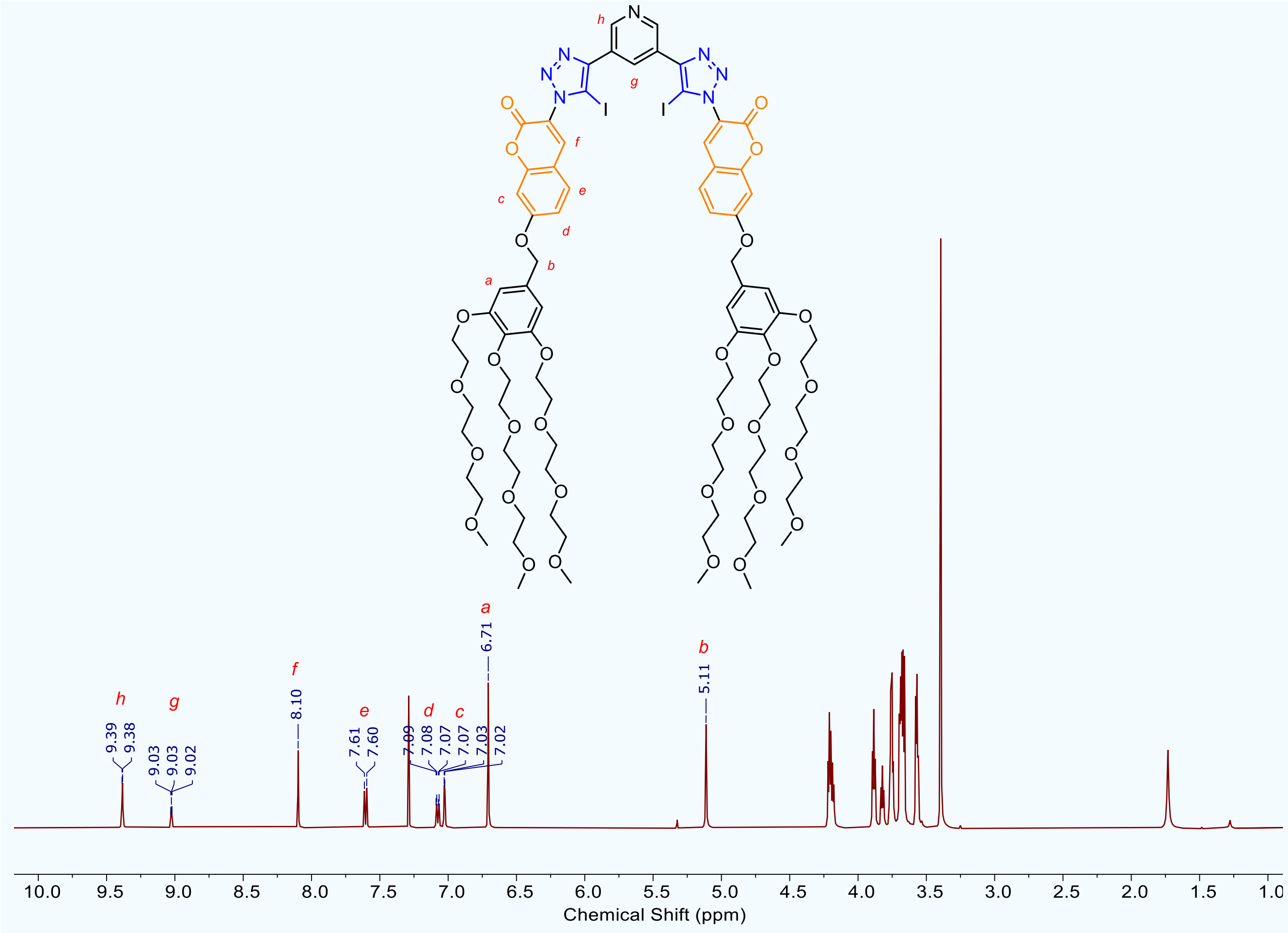

Figure S2-1: Partial ^{1}H NMR spectrum of receptor **XB1** (500 MHz, $CDCl_3$)

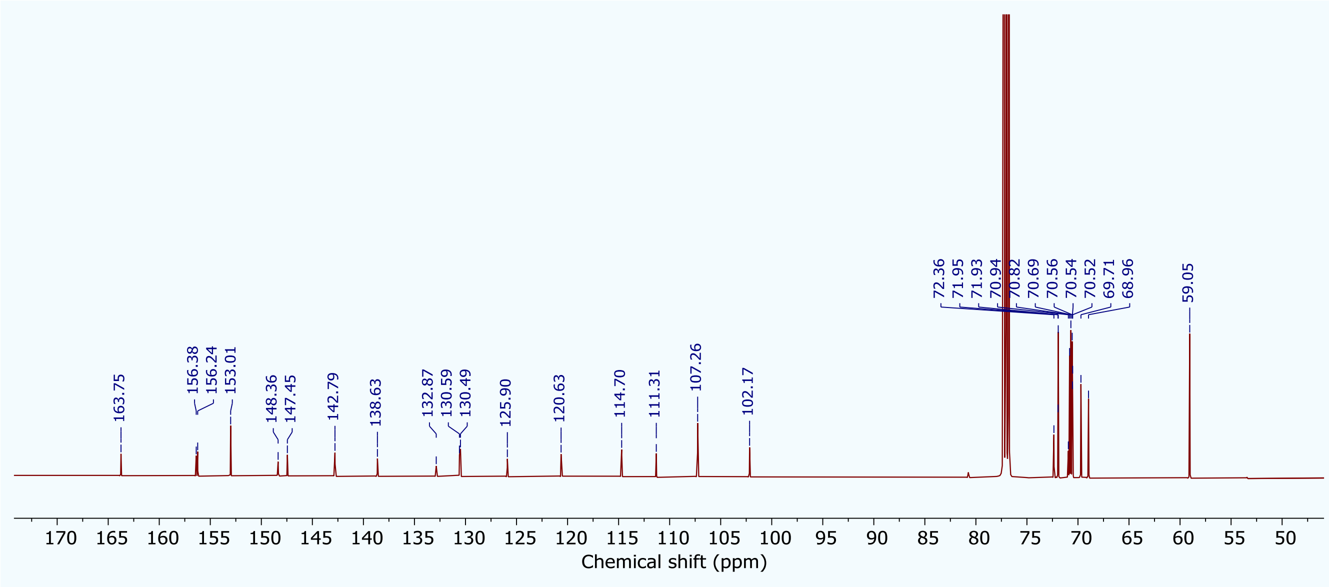

Figure S2-2: Partial ^{13}C NMR spectrum of receptor **XB1** (500 MHz, $CDCl_3$)

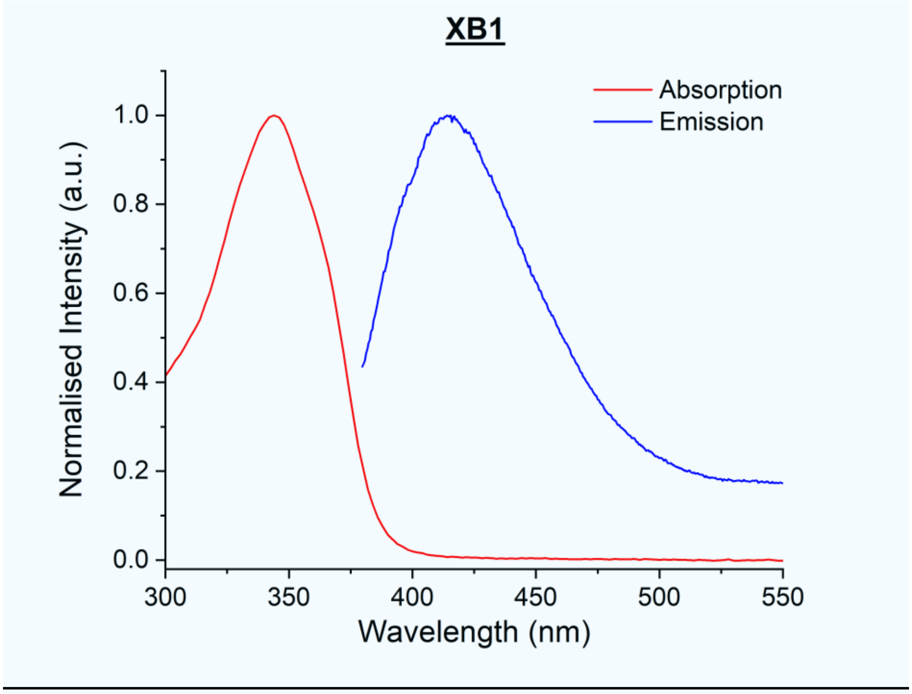

Figure S2-3: Normalised absorption and emission spectra of receptor **XB1**; 298 K, pH = 7.4, 10 mM HEPES aqueous buffer solution.

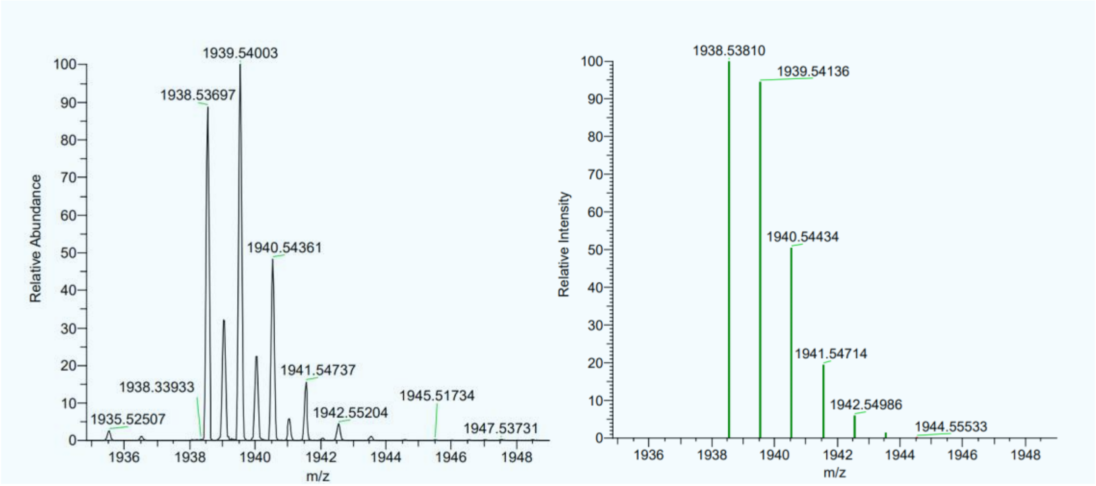

Figure S2-4: High-resolution ESI mass spectrum of **XB1** (Left) and its theoretical calculated spectrum (right).

S2.2. Host receptor **HB1**

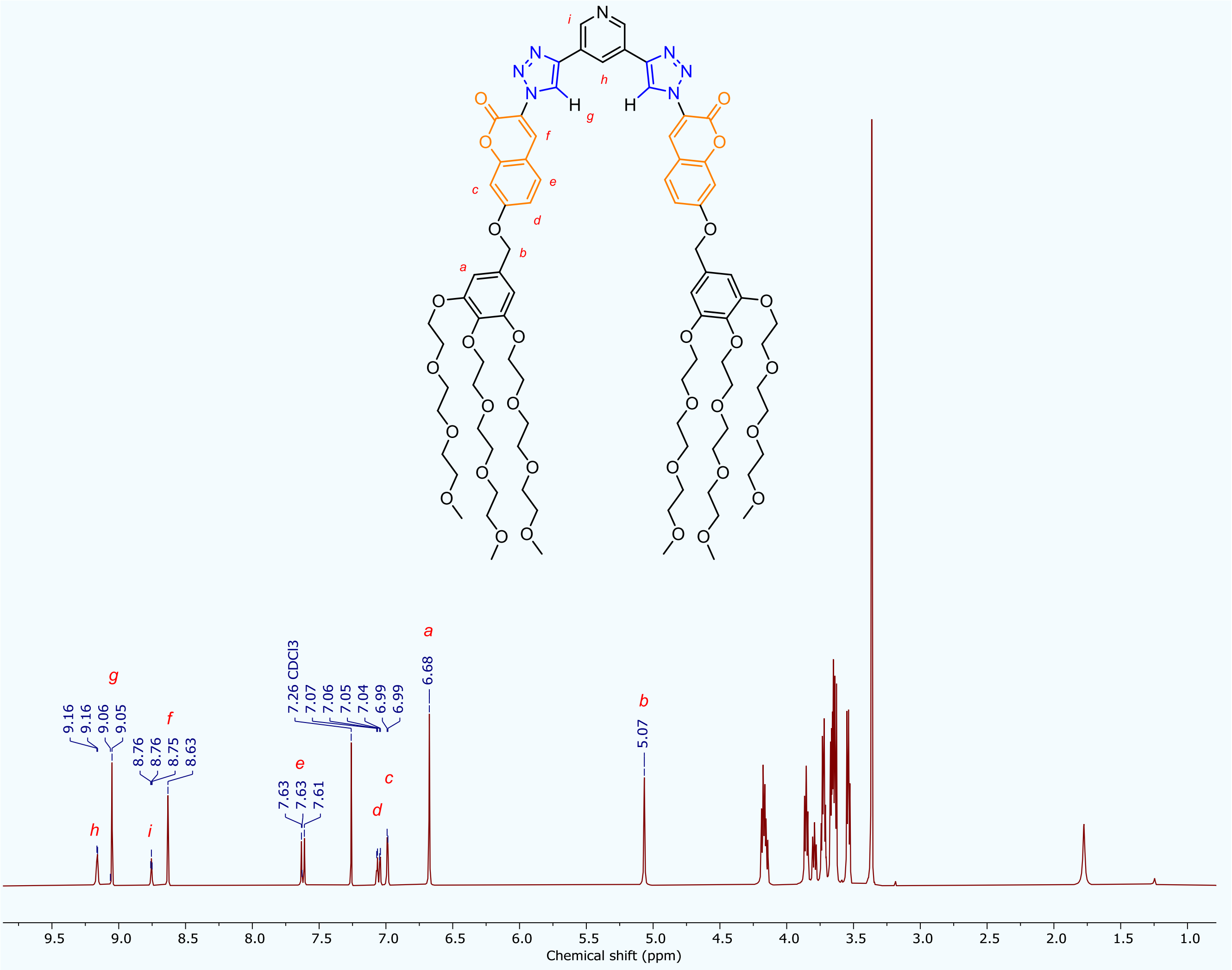

Figure S2-5: Partial ^{1}H NMR spectrum of receptor **HB1** (500 MHz, $CDCl_3$)

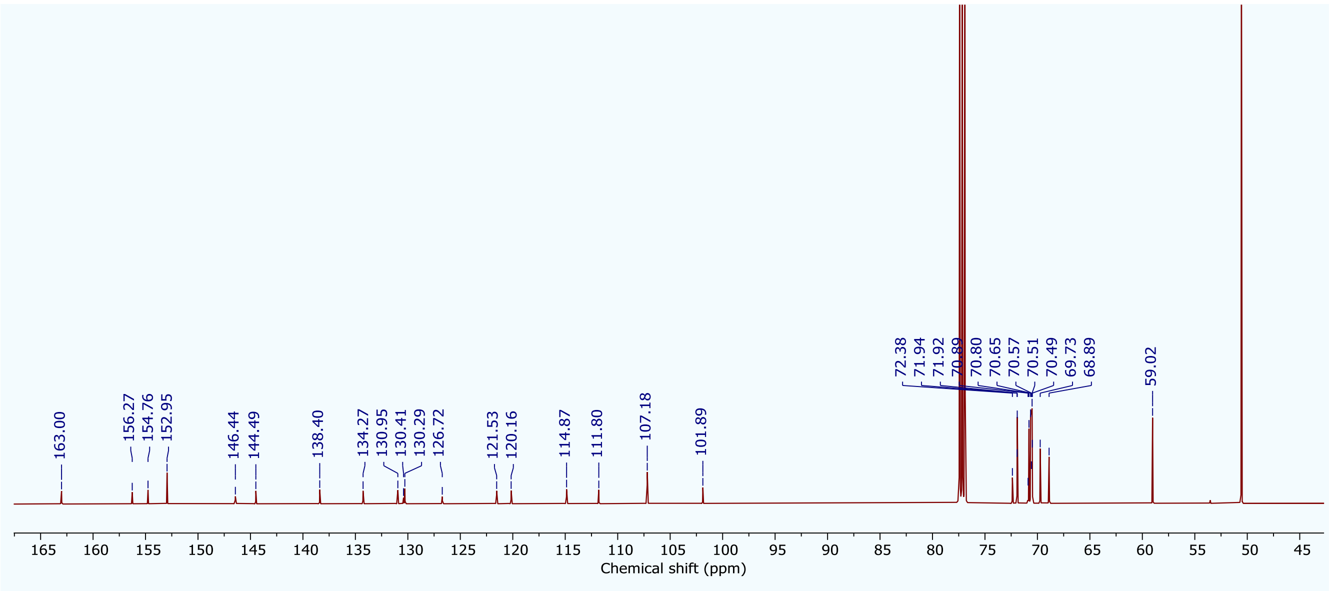

Figure S2-6: Partial ^{13}C NMR spectrum of receptor **HB1** (500 MHz, $CDCl_3$)

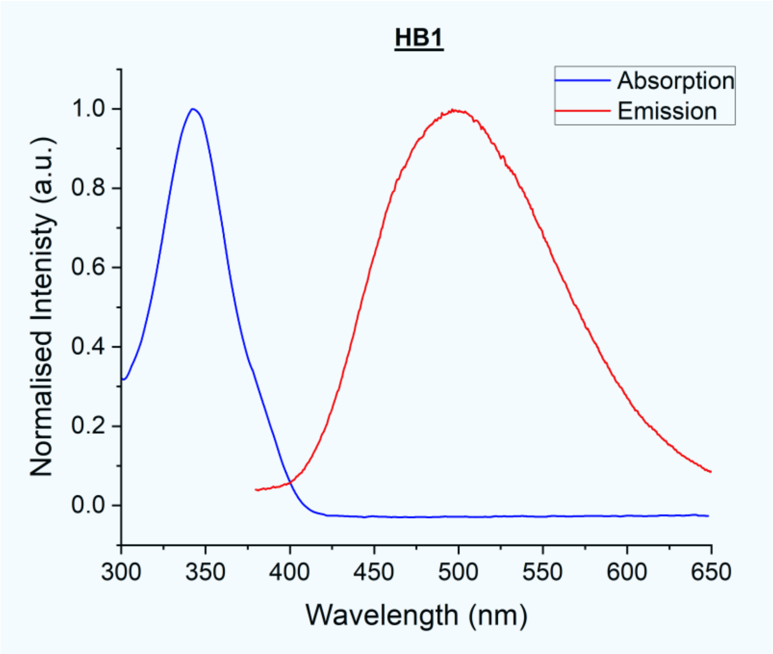

Figure S2-7: Normalised absorption and emission spectra of receptor **HB1**; 298 K, pH = 7.4, 10 mM HEPES aqueous buffer solution

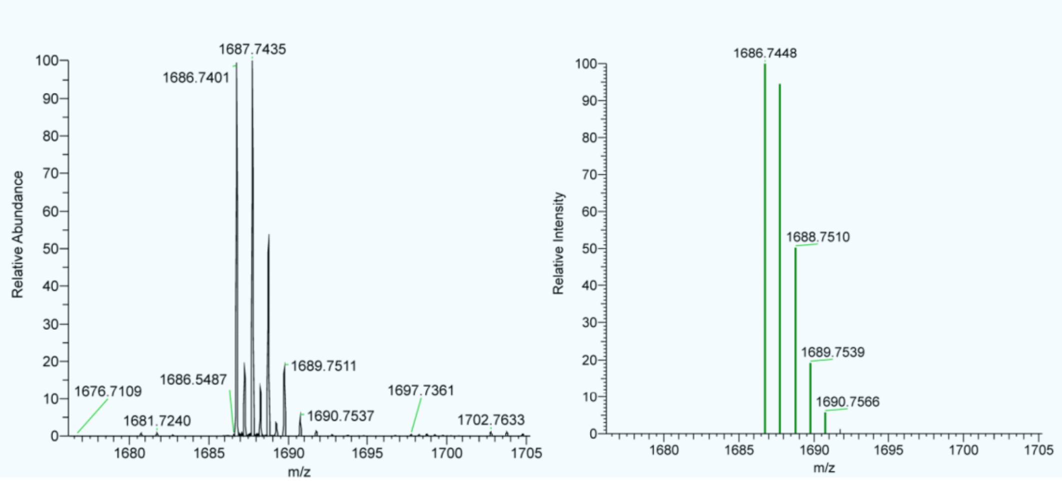

Figure S2-8: High-resolution ESI mass spectrum of **HB1** (Left) and its theoretical calculated spectrum (right).

S2.3. Host receptor **XB2**

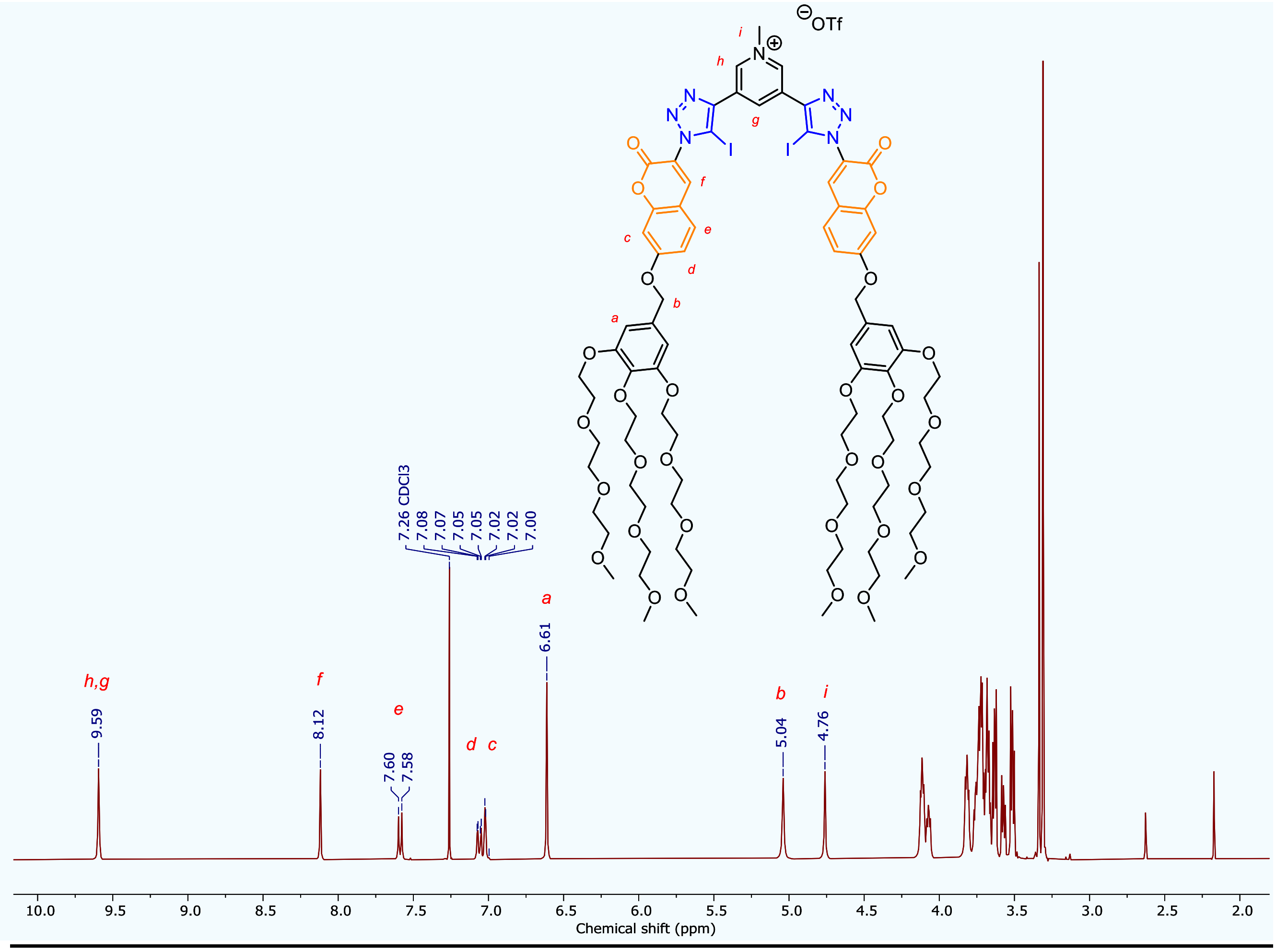

Figure S2-9: Partial ^{1}H NMR spectrum of receptor **XB2** (500 MHz, $CDCl_3$)

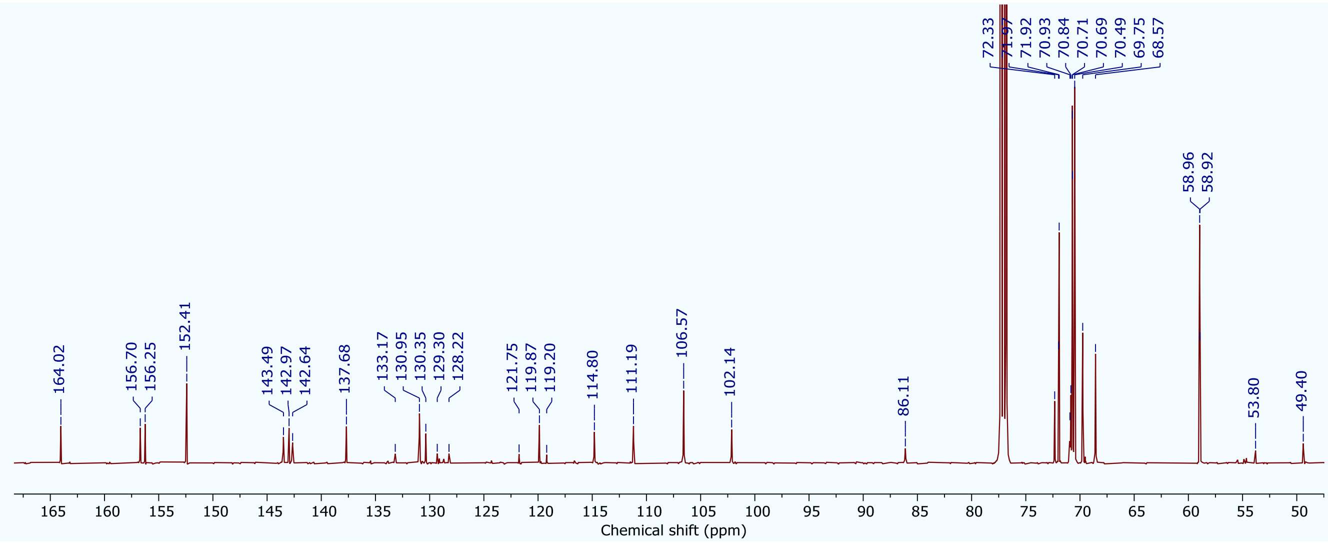

Figure S2-10: Partial ^{13}C NMR spectrum of receptor **XB2** (500 MHz, $CDCl_3$)

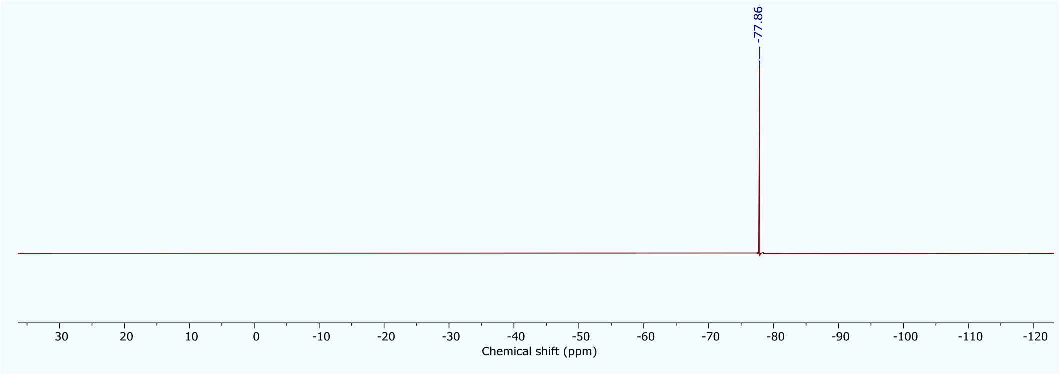

Figure S2-11: Partial ^{19}F NMR spectrum of receptor **XB2** (500 MHz, $CDCl_3$)

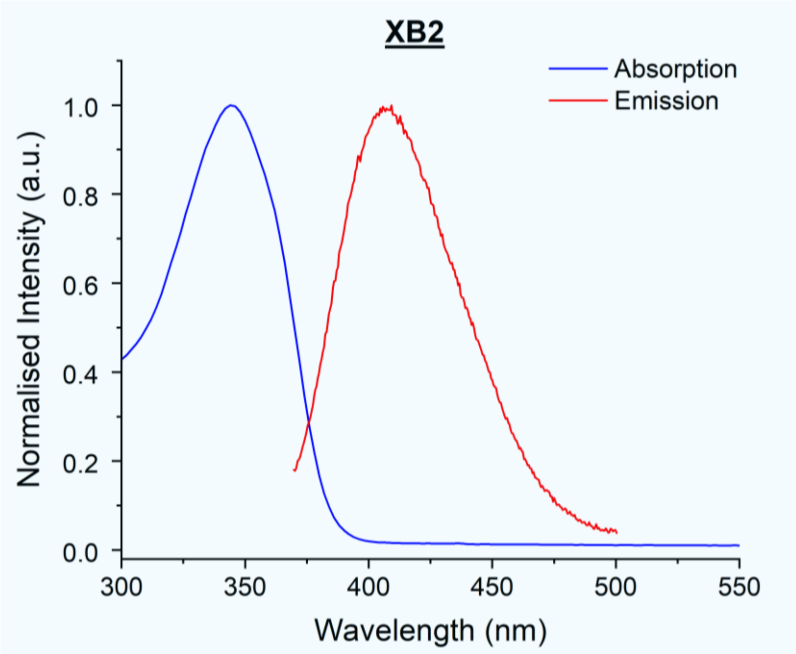

Figure S2-12: Normalised absorption and emission spectra of receptor **XB2**; 298 K, pH = 7.4, 10 mM HEPES aqueous buffer solution.

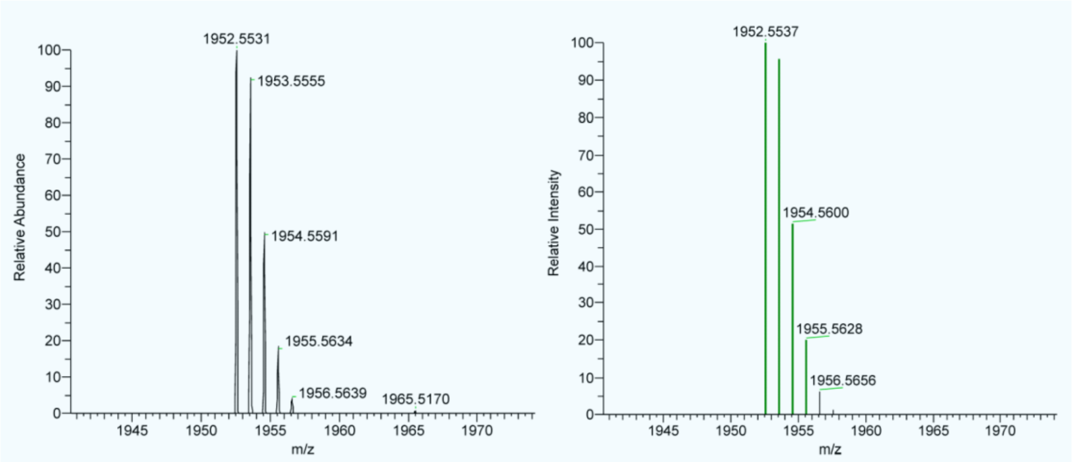

Figure S2-13: High-resolution ESI mass spectrum of **XB2** (Left) and its theoretical calculated spectrum (right).

S2.4. Host receptor **HB2**

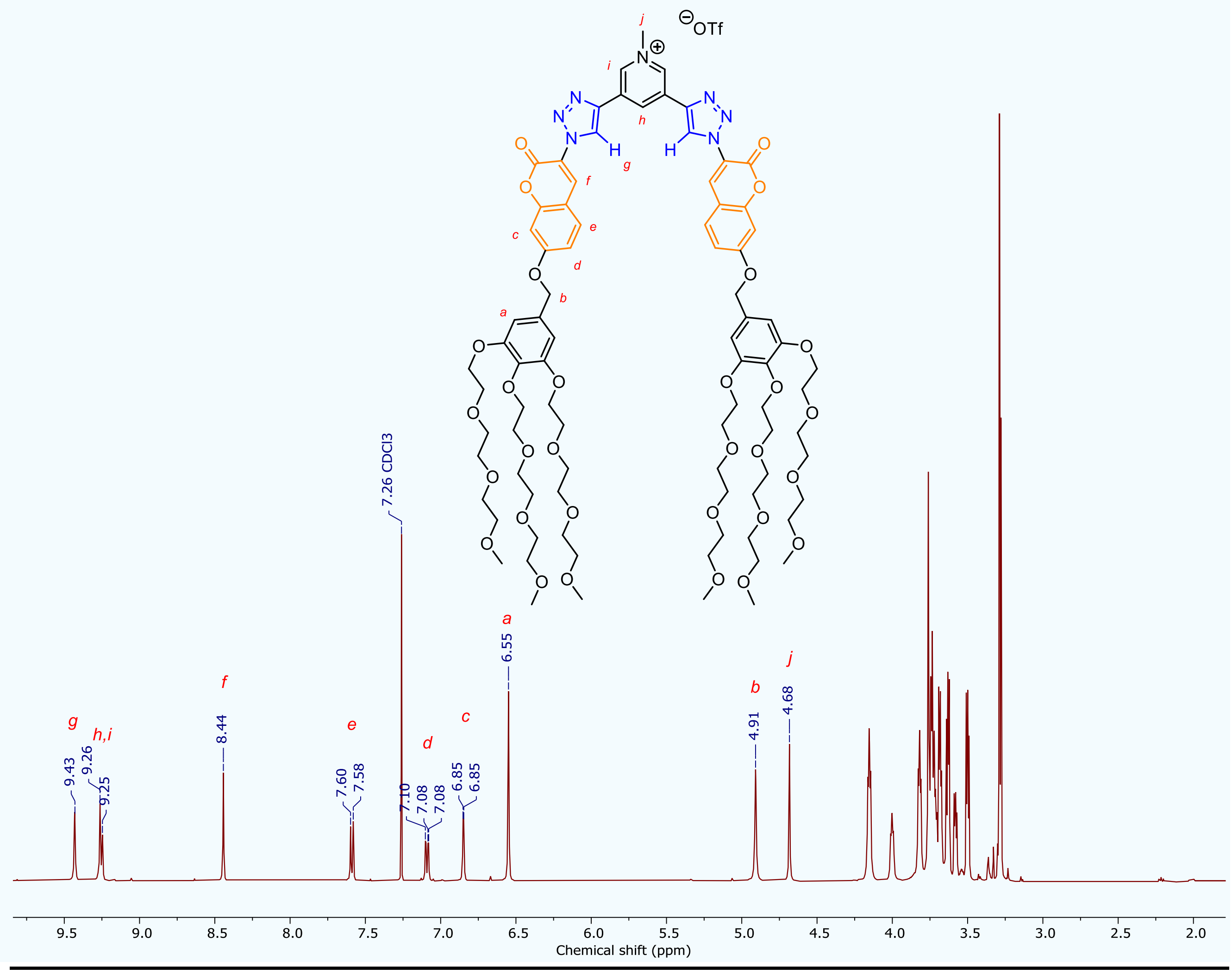

Figure S2-14: Partial ^{1}H NMR spectrum of receptor **HB2** (500 MHz, $CDCl_3$)

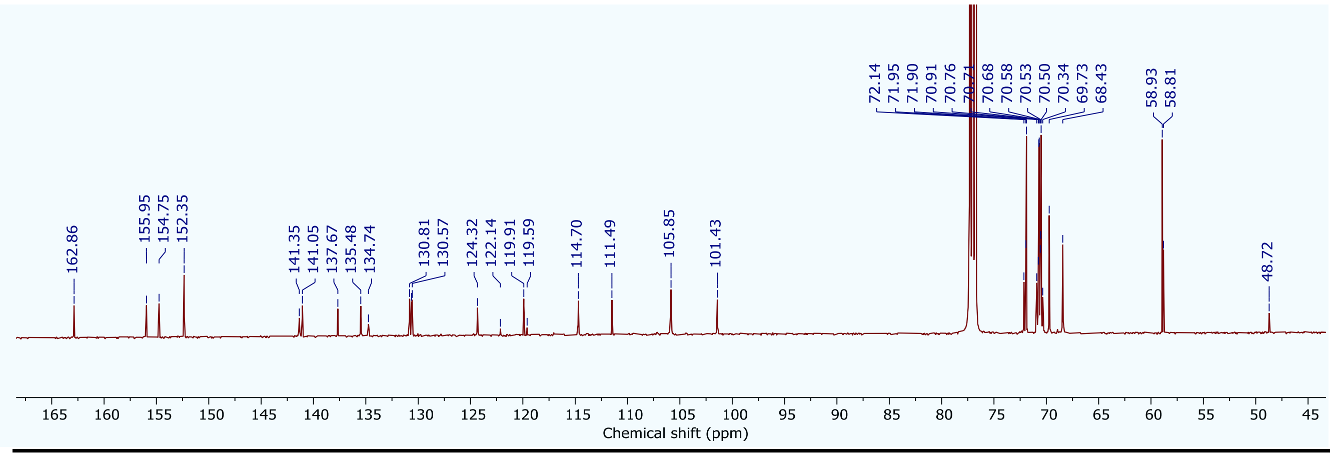

Figure S2-15: Partial ^{13}C NMR spectrum of receptor **HB2** (500 MHz, $CDCl_3$)

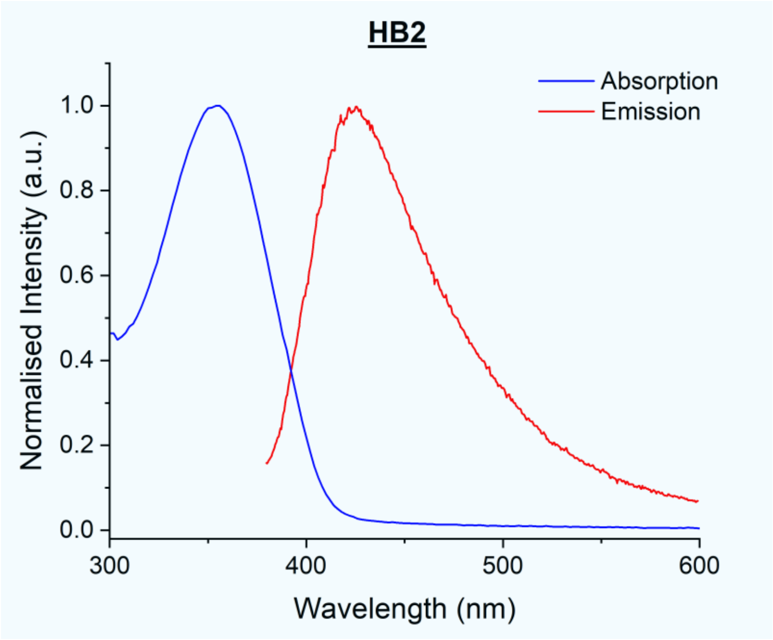

Figure S2-16: Normalised absorption and emission spectra of receptor **HB2**; 298 K, pH = 7.4, 10 mM HEPES aqueous buffer solution

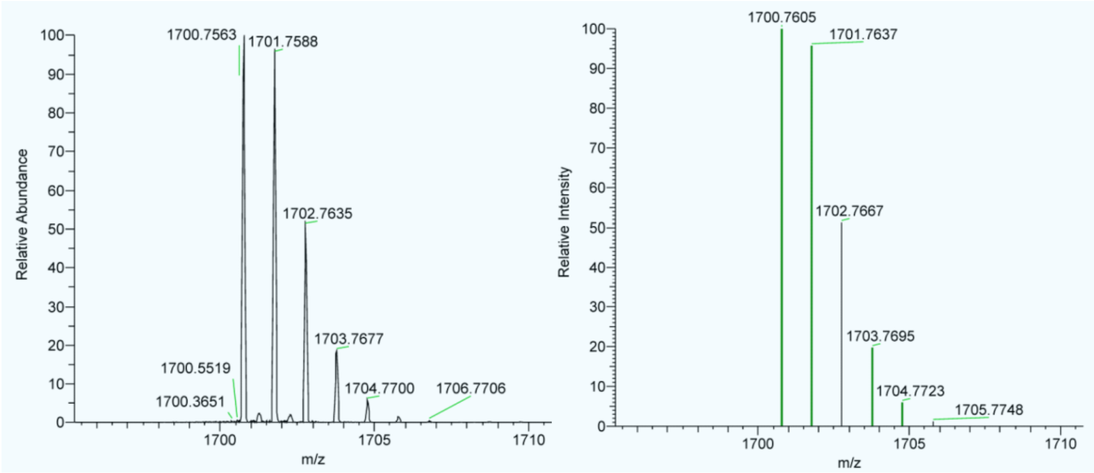

Figure S2-17: High-resolution ESI mass spectrum of **HB2** (Left) and its theoretical calculated spectrum (right).

S2.5. Host receptor **XB3**

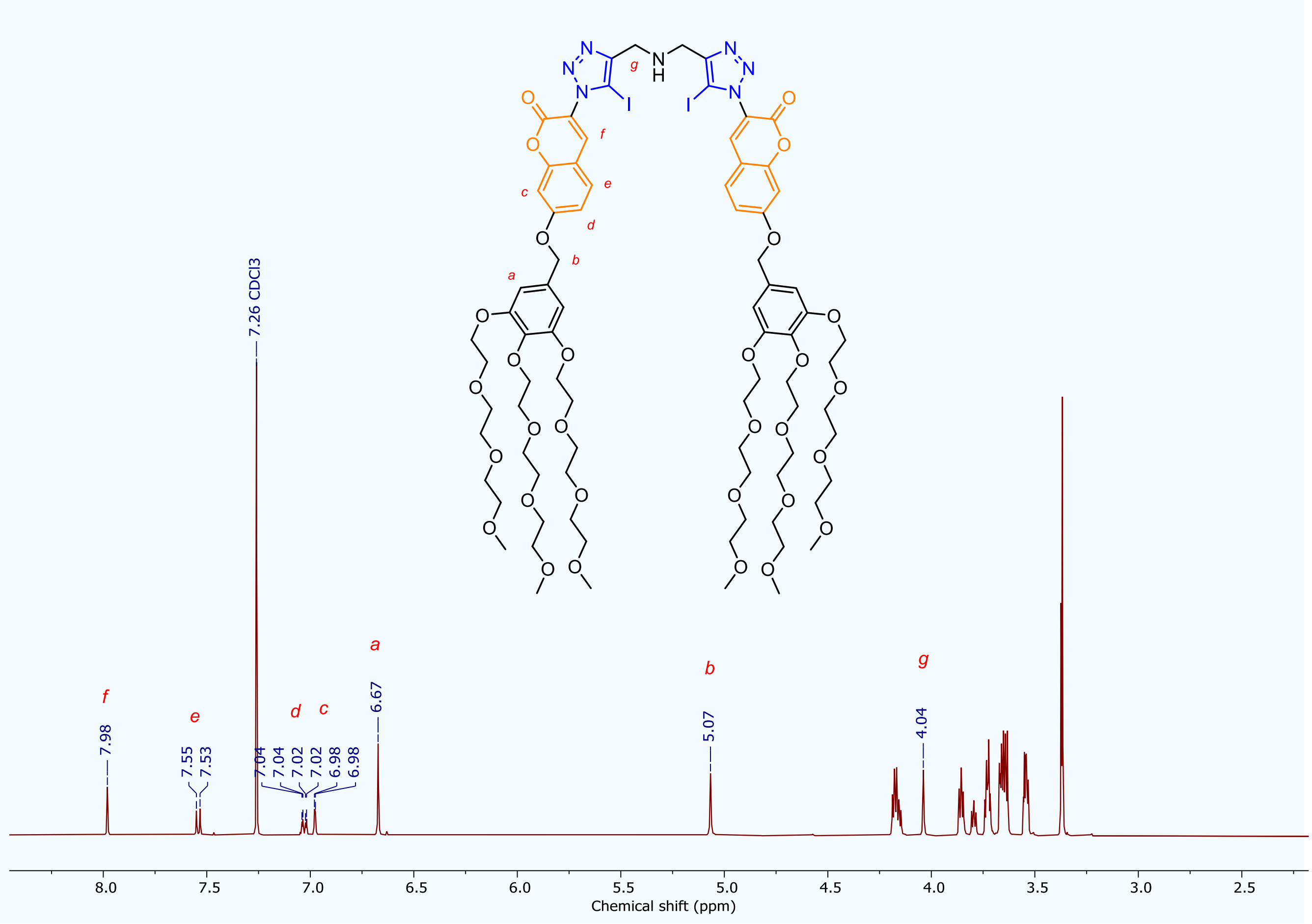

Figure S2-18: Partial ^{1}H NMR spectrum of receptor **XB3** (500 MHz, $CDCl_3$)

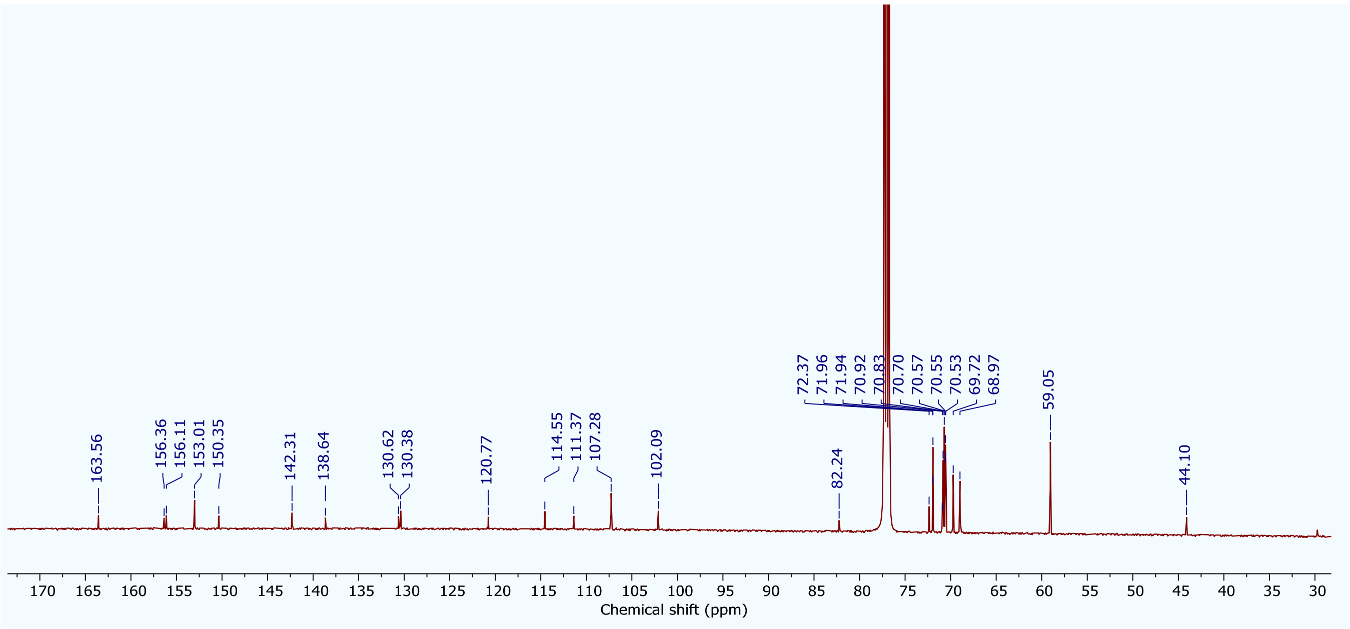

Figure S2-19: Partial ^{13}C NMR spectrum of receptor **HB2** (500 MHz, $CDCl_3$)

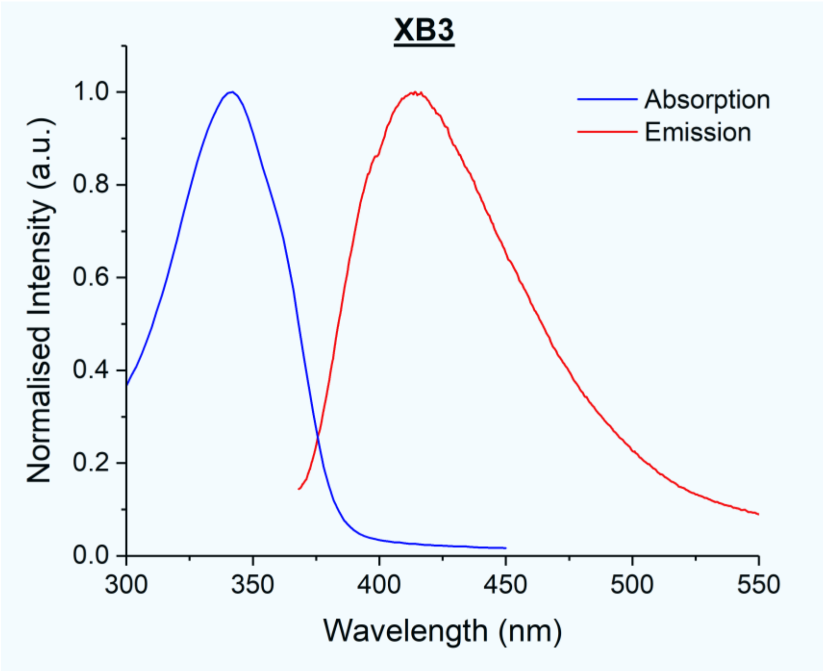

Figure S2-20: Normalised absorption and emission spectra of receptor **XB3**; 298 K, pH = 7.4, 10 mM HEPES aqueous buffer solution.

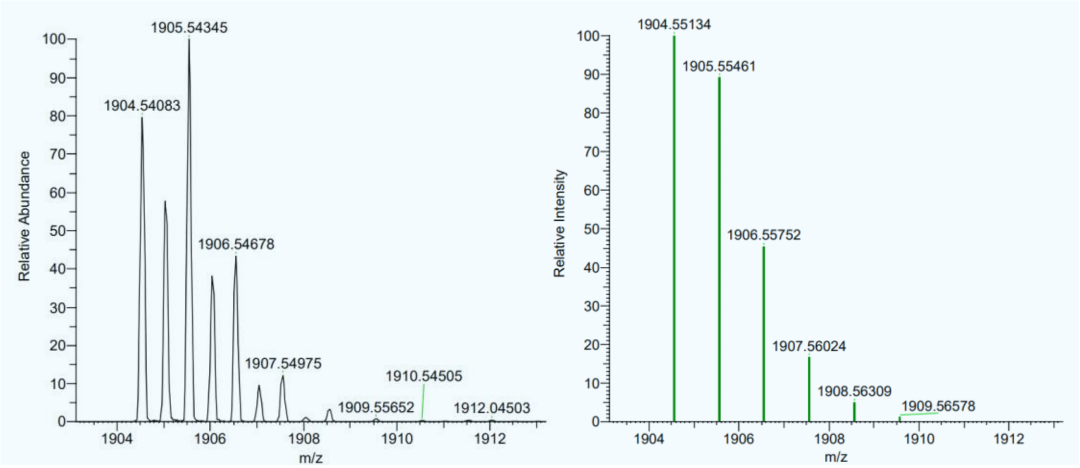

Figure S2-21: High-resolution ESI mass spectrum of **XB3** (Left) and its theoretical calculated spectrum (right).

S2.6. Host receptor **HB3**

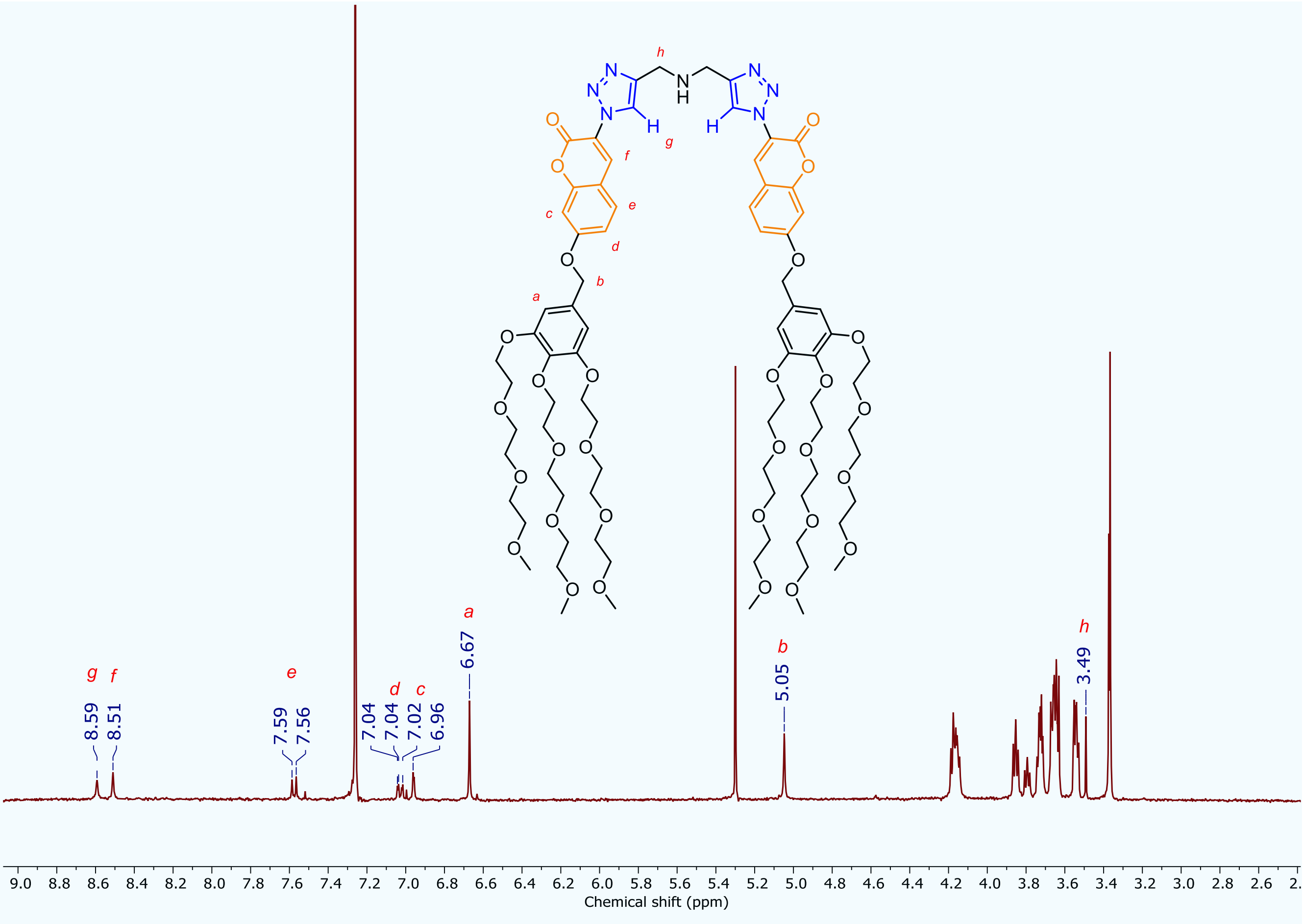

Figure S2-22: Partial ^{1}H NMR spectrum of receptor **HB3** (500 MHz, $CDCl_3$)

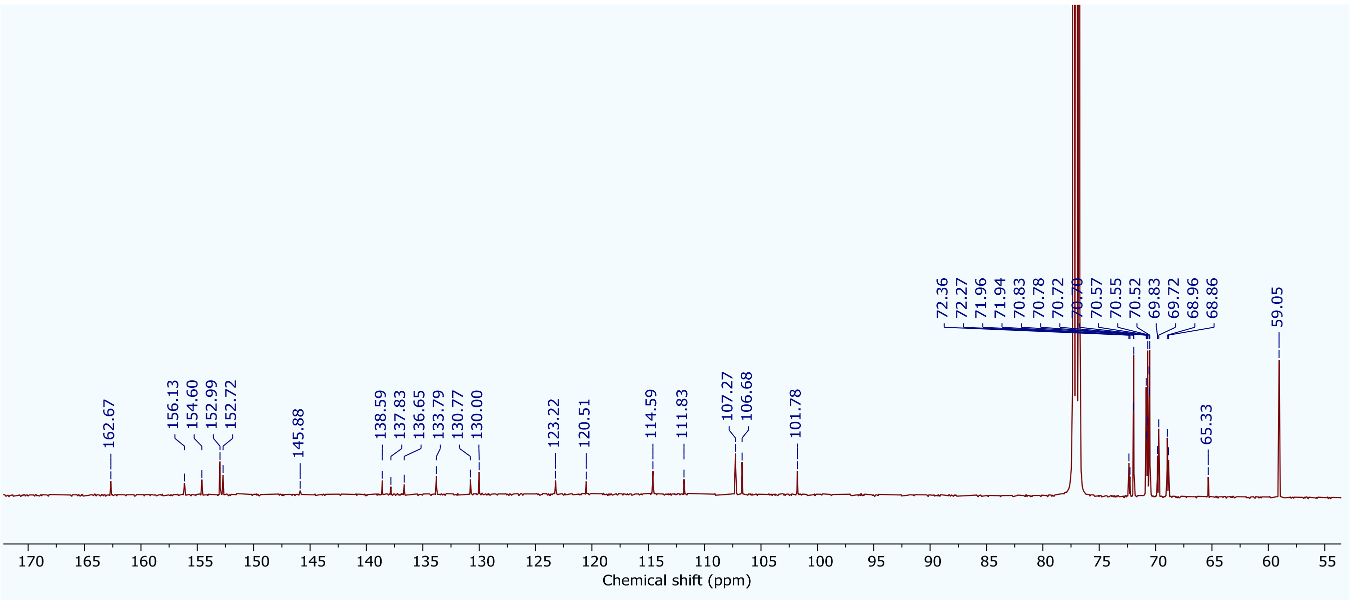

Figure S2-23: Partial ^{13}C NMR spectrum of receptor **HB3** (500 MHz, $CDCl_3$)

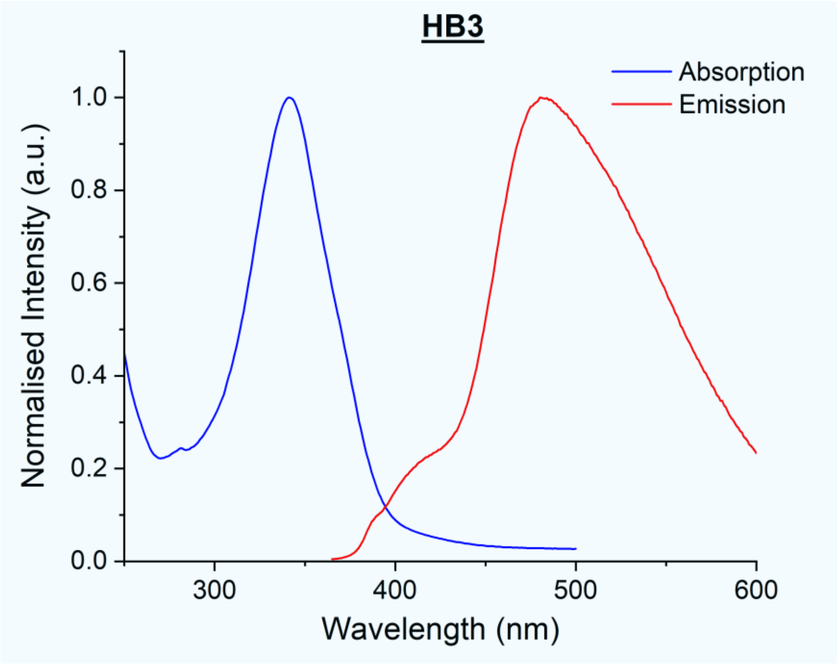

Figure S2-24: Normalised absorption and emission spectra of receptor **HB3**; 298 K, pH = 7.4, 10 mM HEPES aqueous buffer solution.

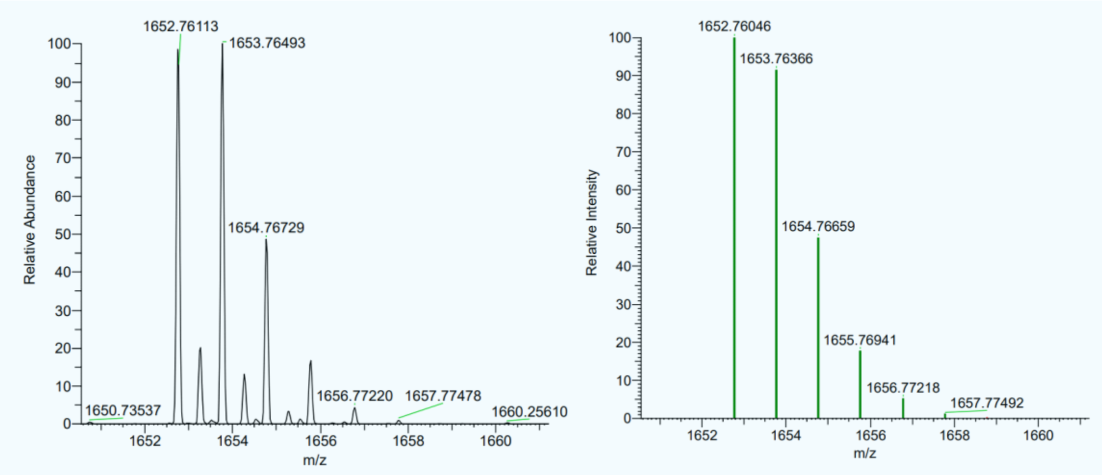

Figure S2-25: High-resolution ESI mass spectrum of **HB3** (Left) and its theoretical calculated spectrum (right).

S2.7. Photo-Physical Summary

Table S2-1: A: Energies (wavelengths λ) of absorption and emission for receptors XB1-3 and HB1-3 (10 µM) in 10 mM HEPES aqueous solution (pH 7.4, 298 K).

Receptor	$\lambda_{max\ abs}$ / nm	$\lambda_{max\ ems}$ / nm	Stokes Shift / nm
**XB1**	344	414	70
**HB1**	342	498	156
**XB2**	344	408	64
**HB2**	354	424	70
**XB3**	342	412	70
**HB3**	342	484	142

The large red shift and resultant Stokes shifts for HB1 and HB3 were attributed to intramolecular bonds between the proto-triazole and coumarin carbonyl. For HB2, due to its enhanced HB characteristics, solvent interactions with H_2O were believed to disrupt the formation of this intramolecular bond.

S3. Fluorescence Titrations

Fluorescence titration experiments were performed using a Horiba Duetta at 293 K. The host molecules (**XB1-3**, and **HB1-3**) were dissolved in 10 mM HEPES aqueous buffer solution at pH 7.4 at a concentration of 10 μM. Na salts of the appropriate anion were dissolved in the solution of the host molecule to obtain a concentration. Aliquots of the anion solution were added to 1.00 mL of the host solution in a quartz cuvette, where the sample was then thoroughly mixed before fluorescence spectra were recorded. Association constants were obtained by global analysis using BindFit[7,8], using a host-guest 1:1 binding model.

For the anion titrations, the recorded spectra during the titration are shown. The arrows on the recorded spectra denote the direction of change in fluorescence intensity (decrease) upon anion addition. The legend indicates the number of equivalents of anions added and their corresponding spectrum. Receptors **HB1-3** showed no significant fluorescence diminutions, suggesting no binding.

S3.1. **XB1**

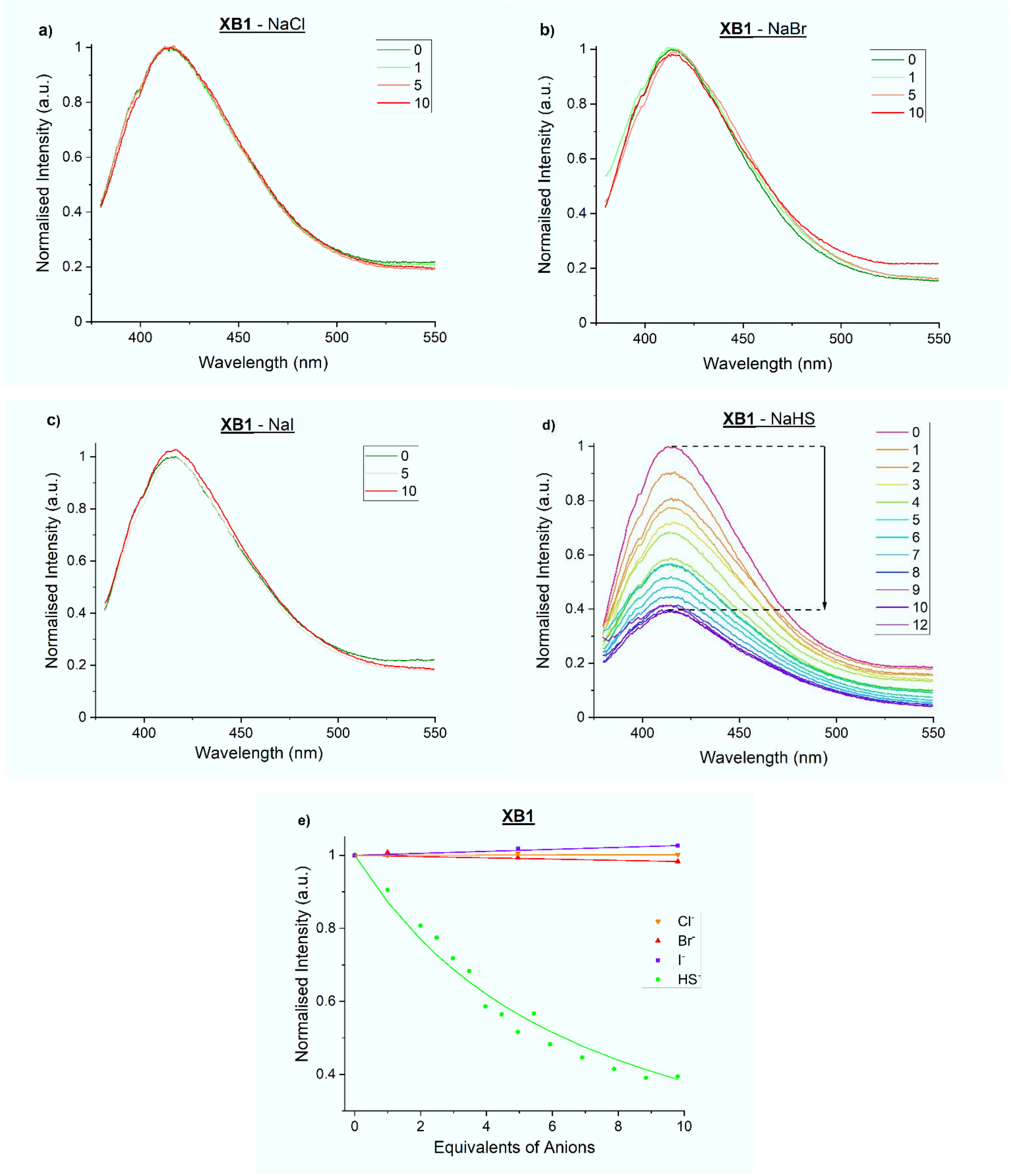

Figure S3-1: Fluorescence titration emission change of 10 µM **XB1** on addition of **a)** NaCl **b)** NaBr, **c)** NaI **d)** NaHS in pH 7.4 10 mM aqueous HEPES buffer solution **e)** BindFit analysis fit of fluorescence titration data monitoring λ_{max} (400 – 420 nm) of receptor **XB1** upon addition of NaCl, NaBr, NaI and NaHS in pH 7.4 10 mM aqueous HEPES buffer solution.

S3.2. **HB1**

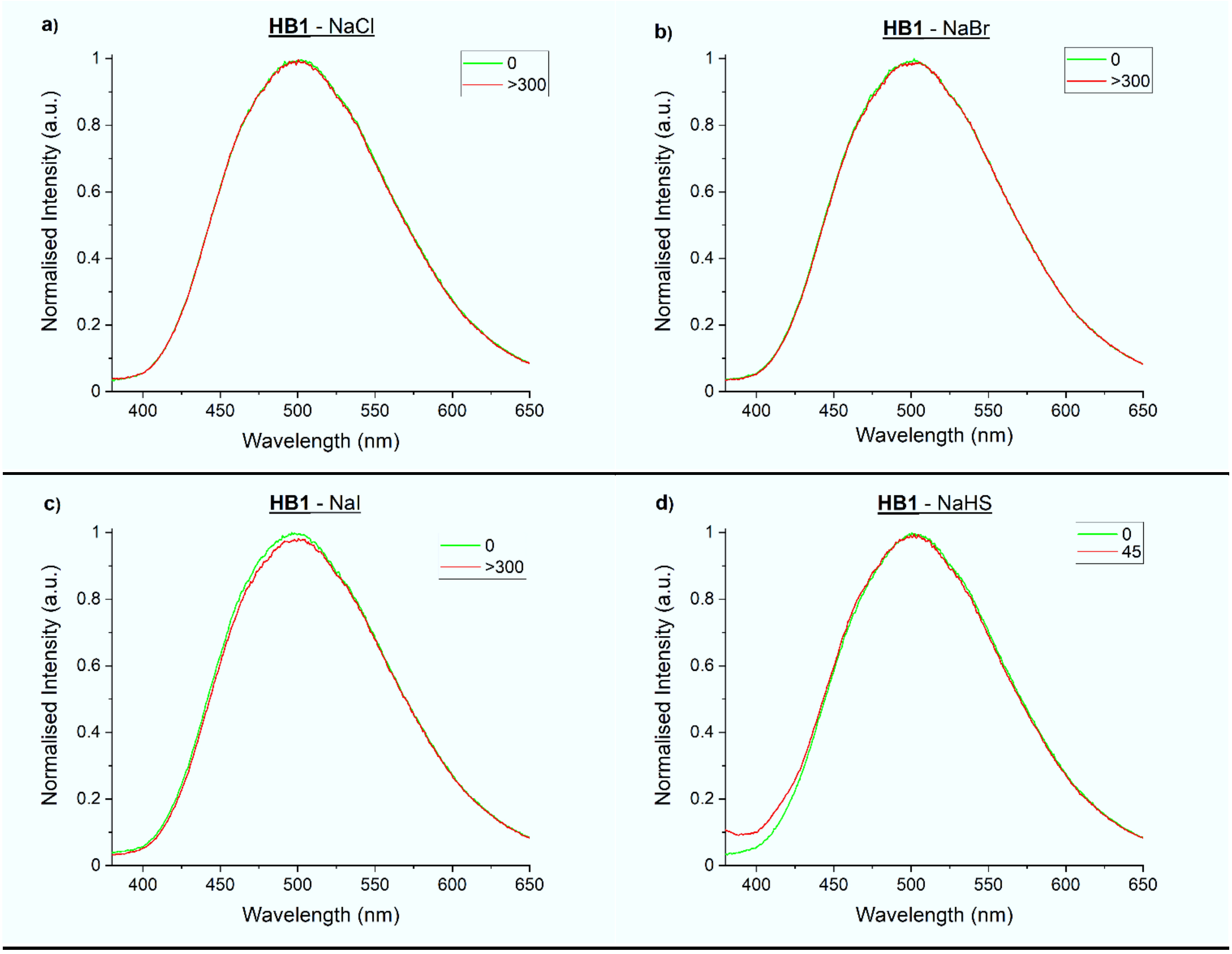

Figure S3-2: Fluorescence titration emission change of 10 µM **HB1** on addition of **a)** NaCl **b)** NaBr, **c)** NaI **d)** NaHS in pH 7.4 10 mM aqueous HEPES buffer solution.

S3.2. **XB2**

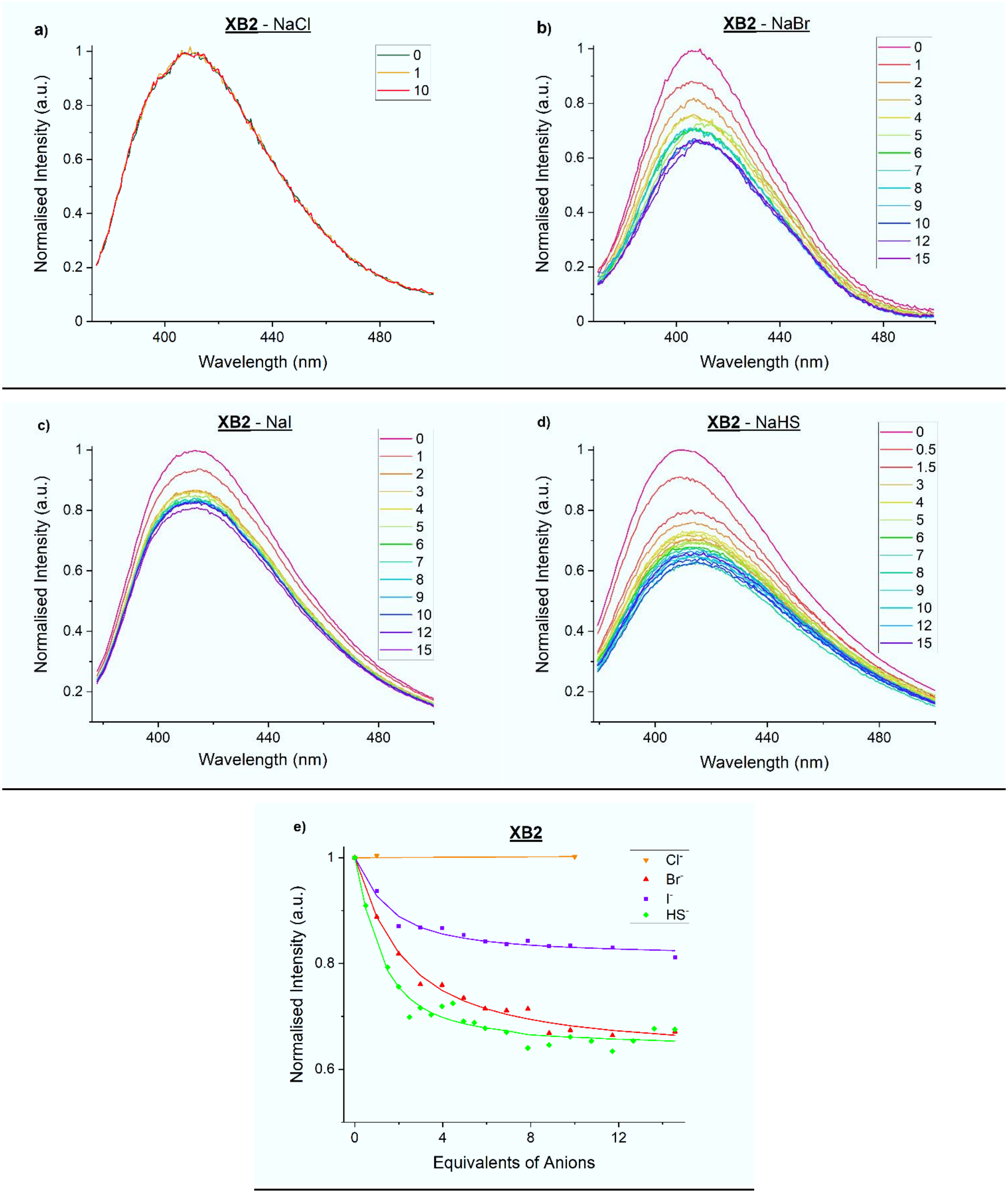

Figure S3-3: Fluorescence titration emission change of 10 µM **XB2** on addition of **a)** NaCl **b)** NaBr, **c)** NaI **d)** NaHS in pH 7.4 10 mM aqueous HEPES buffer solution **e)** BindFit analysis fit of fluorescence titration data monitoring λ_{max} (400 – 420 nm) of receptor **XB2** upon addition of NaCl, NaBr, NaI and NaHS in pH 7.4 10 mM aqueous HEPES buffer solution.

S3.4. **HB2**

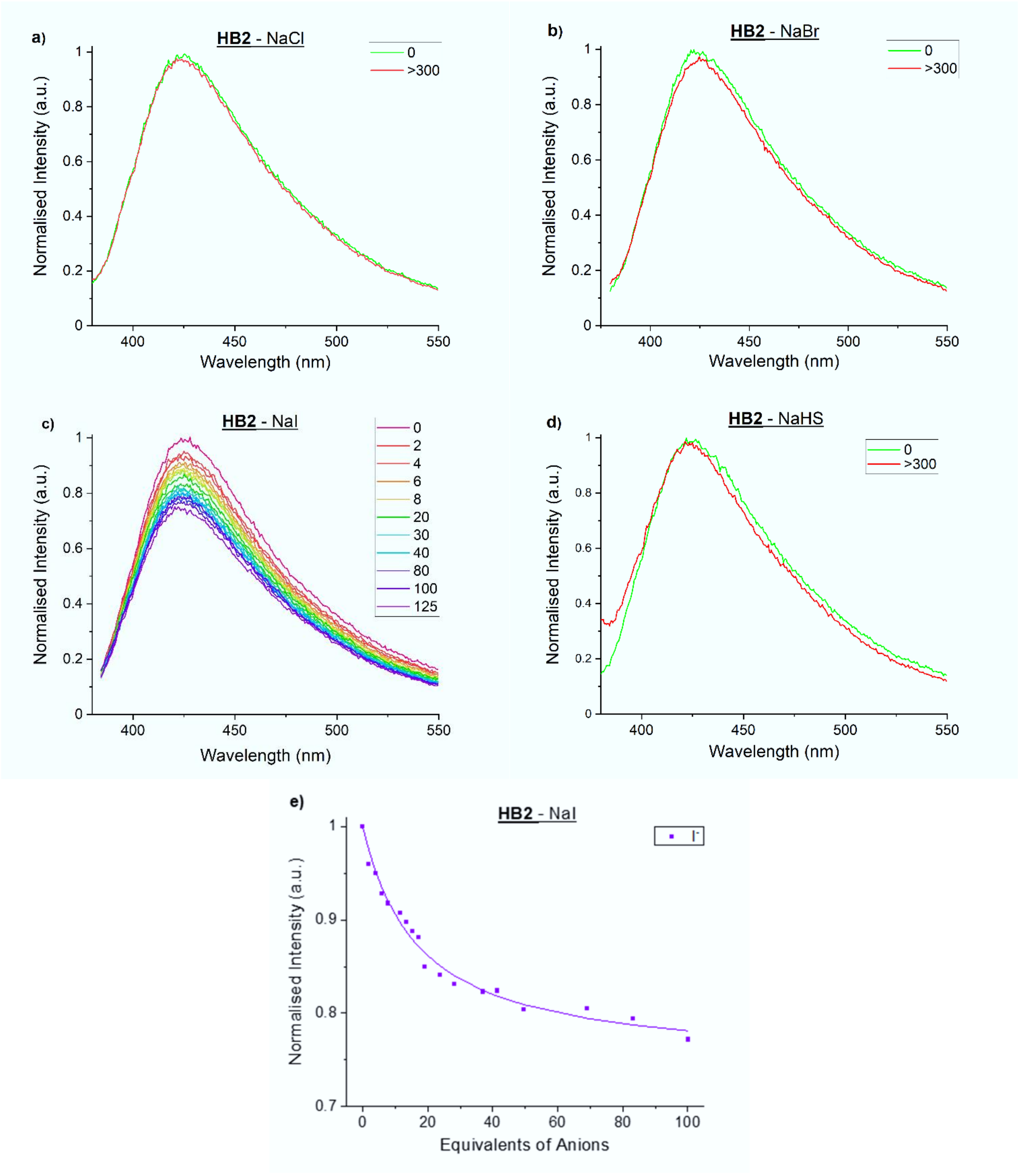

Figure S3-4: Fluorescence titration emission change of 10 µM **HB2** on addition of **a)** NaCl **b)** NaBr, **c)** NaI **d)** NaHS in pH 7.4 10 mM aqueous HEPES buffer solution **e)** BindFit analysis fit of fluorescence titration data monitoring HB2 λ_{max} (426 nm) of receptor **XB2** upon addition of NaI in pH 7.4 10 mM aqueous HEPES buffer solution.

S3.3. **XB3**

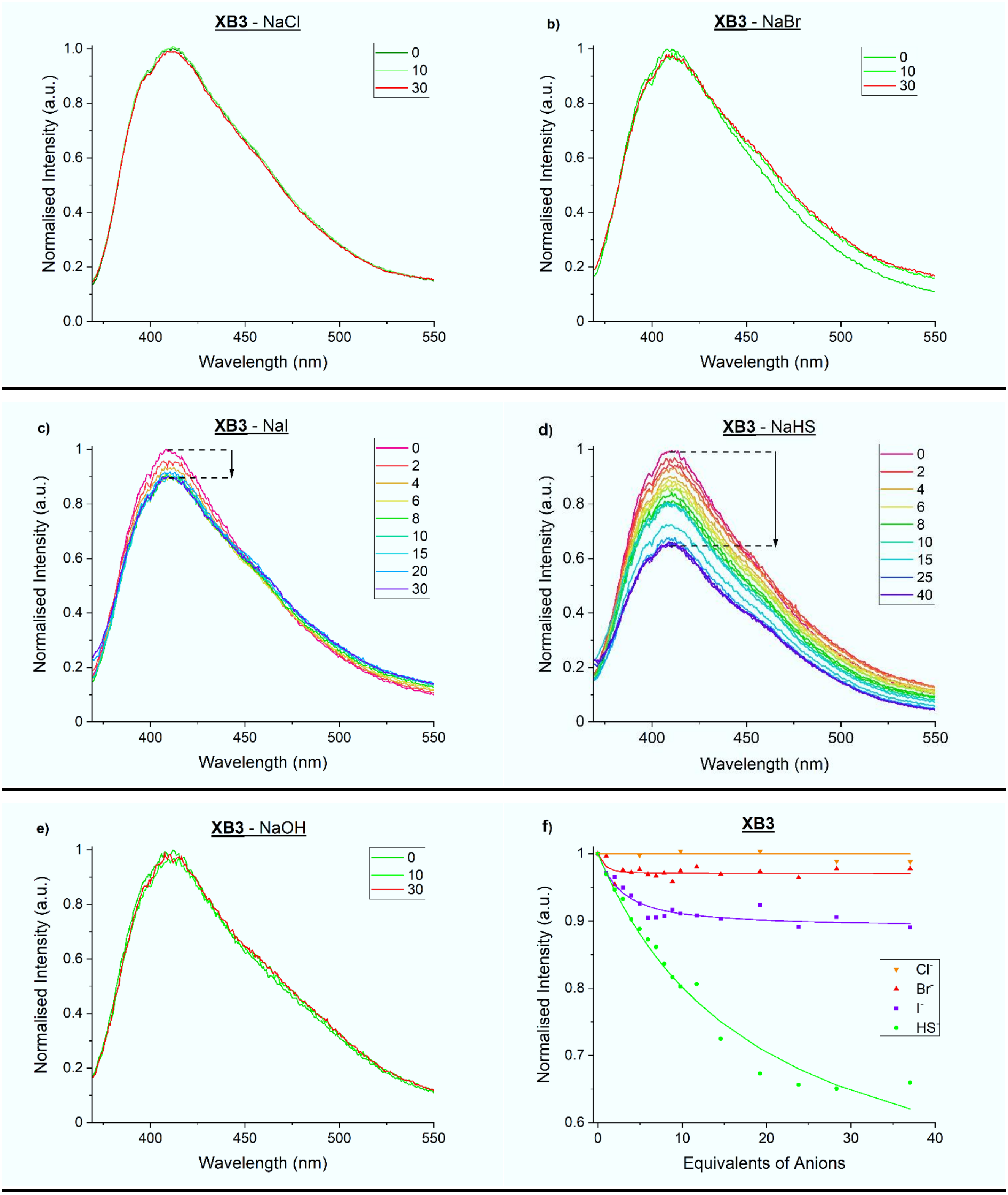

Figure S3-5: Fluorescence titration emission change of 10 µM **XB3** on addition of **a)** NaCl **b)** NaBr, **c)** NaI **d)** NaHS **e)** NaOH in pH 7.4 10 mM aqueous HEPES buffer solution **e)** BindFit analysis fit of fluorescence titration data monitoring λ_{max} (400 – 420 nm) of receptor **XB3** upon addition of NaCl, NaBr, NaI and NaHS in pH 7.4 10 mM aqueous HEPES buffer solution.

HB3

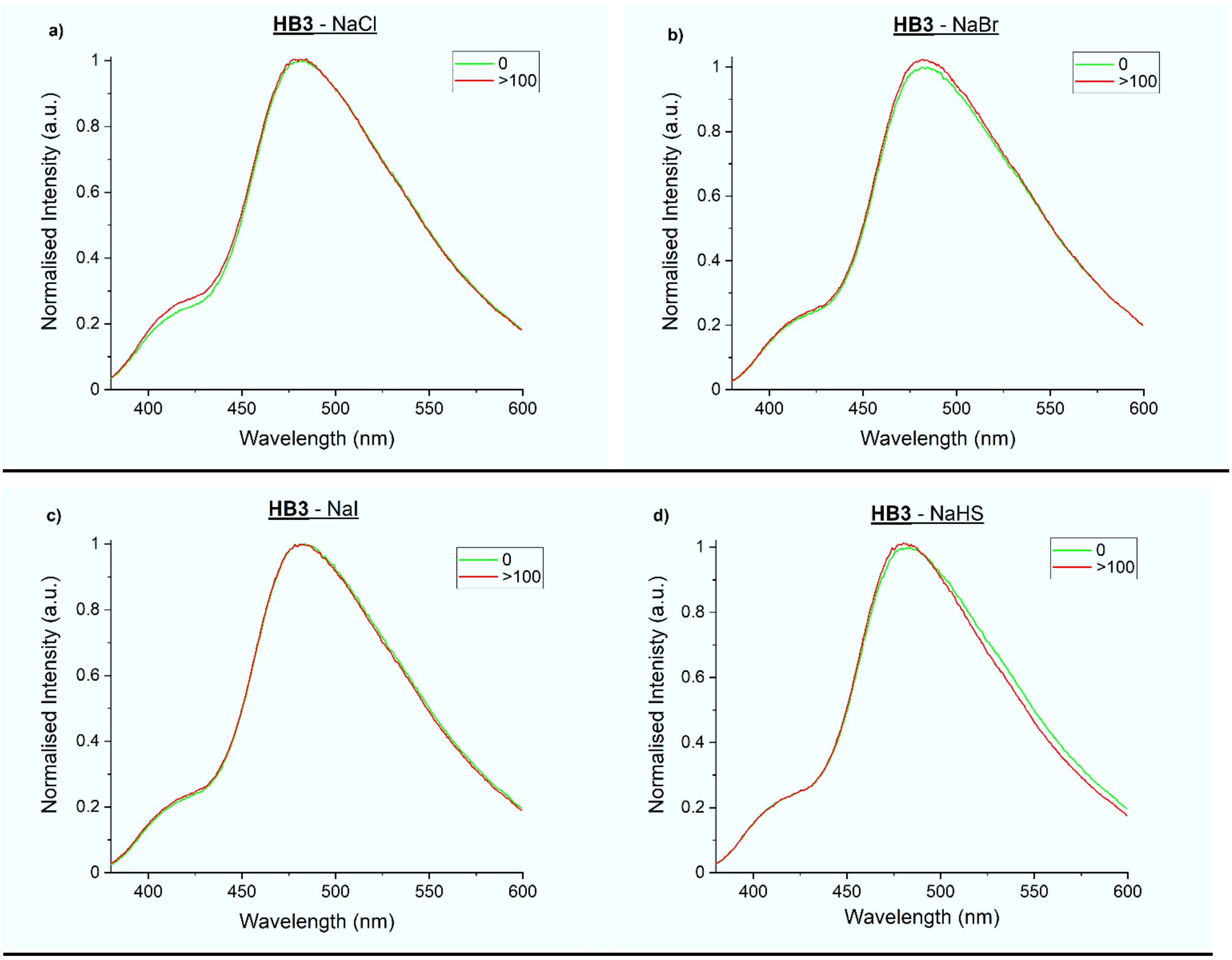

Figure S3-6: Fluorescence titration emission change of 10 µM **HB3** on addition of **a)** NaCl **b)** NaBr, **c)** NaI **d)** NaHS in pH 7.4 10 mM aqueous HEPES buffer solution.

S4. Reversibility Studies

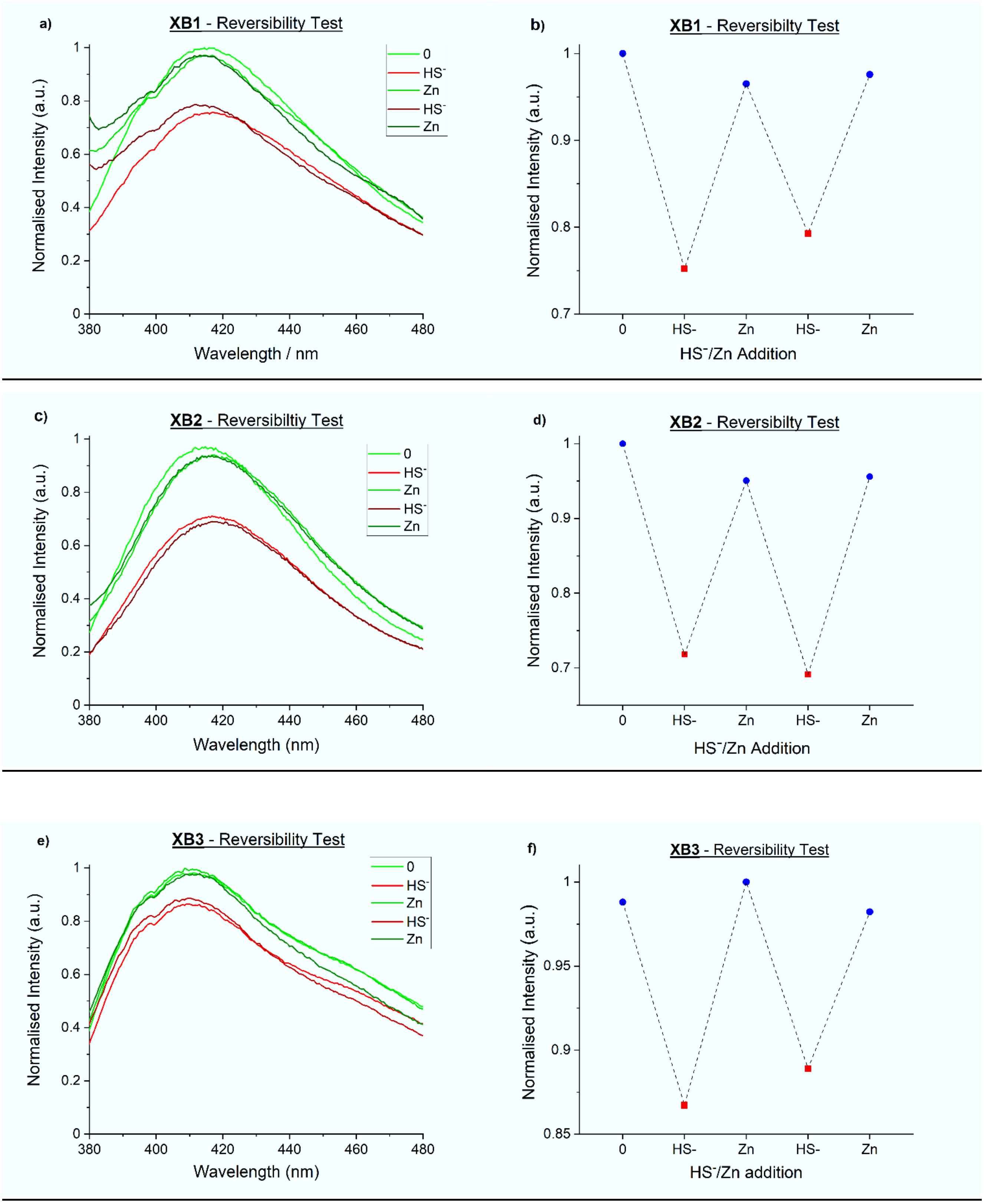

Figure S4-1: Recorded emission spectra of **a) XB1 c) XB2 e) XB3** upon addition of 5 equivalents of NaHS and $Zn(OTf)_2$, repeated for two cycles. Plotted averaged peak normalised intensity (400 – 420 nm) with each cycle for **b) XB1 d) XB2 f) XB3**.

S5. pKa Experiments

The pKa of XB3 and HB3 were experimentally determined by monitoring the change in emission intensity with pH. Two pKa values were found for each receptor, the higher value corresponds with the central secondary amine, whilst the lower is believed to be from the triazole; the lower than expected value is justified by the electron withdrawing nature of the coumarin.

S5.1. XB3

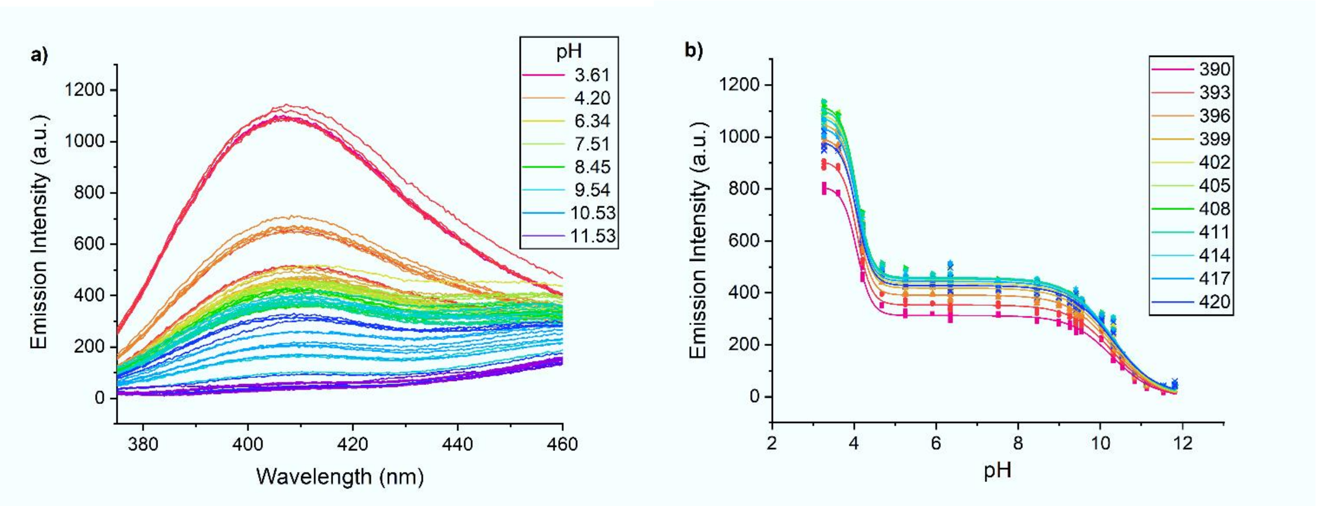

Figure S5-1: a) XB3 emission intensity change with decreasing pH; b) Emission intensity change for select wavelengths with changing pH; pKa_1 = 4.07 (± 0.01), pKa_2 = 10.29 (± 0.01).

S5.2. HB3

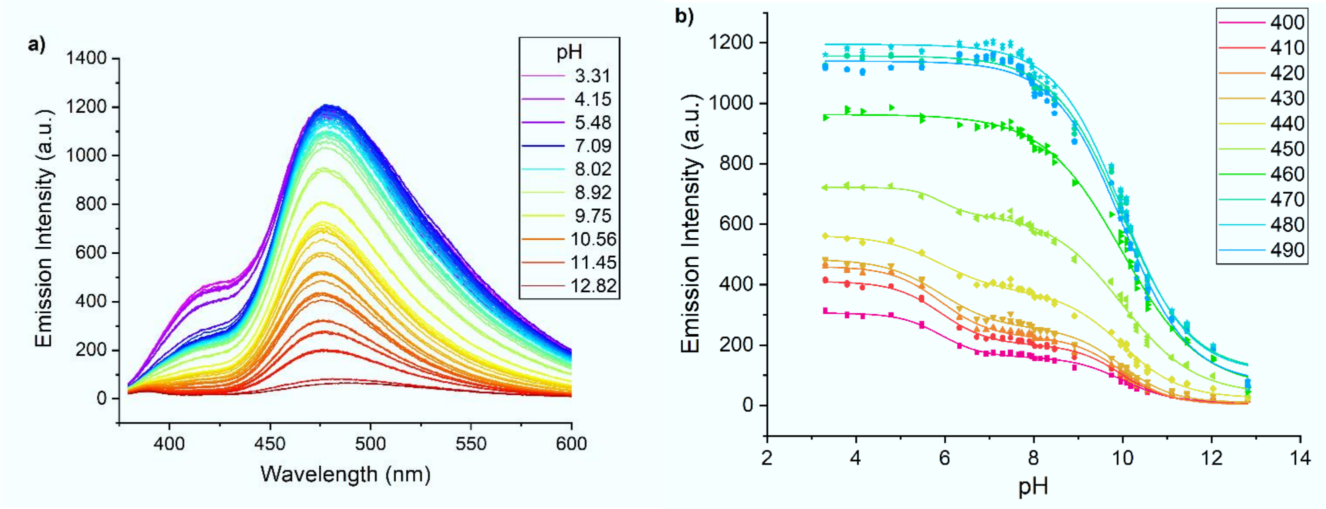

Figure S5-2: a) HB3 emission intensity change with decreasing pH; b) Emission intensity change for select wavelengths with changing pH; pKa_1 = 5.91 (± 0.07), pKa_2 = 9.98 (± 0.01).

S6. MS Experiments

S6.1. MS – H_2O

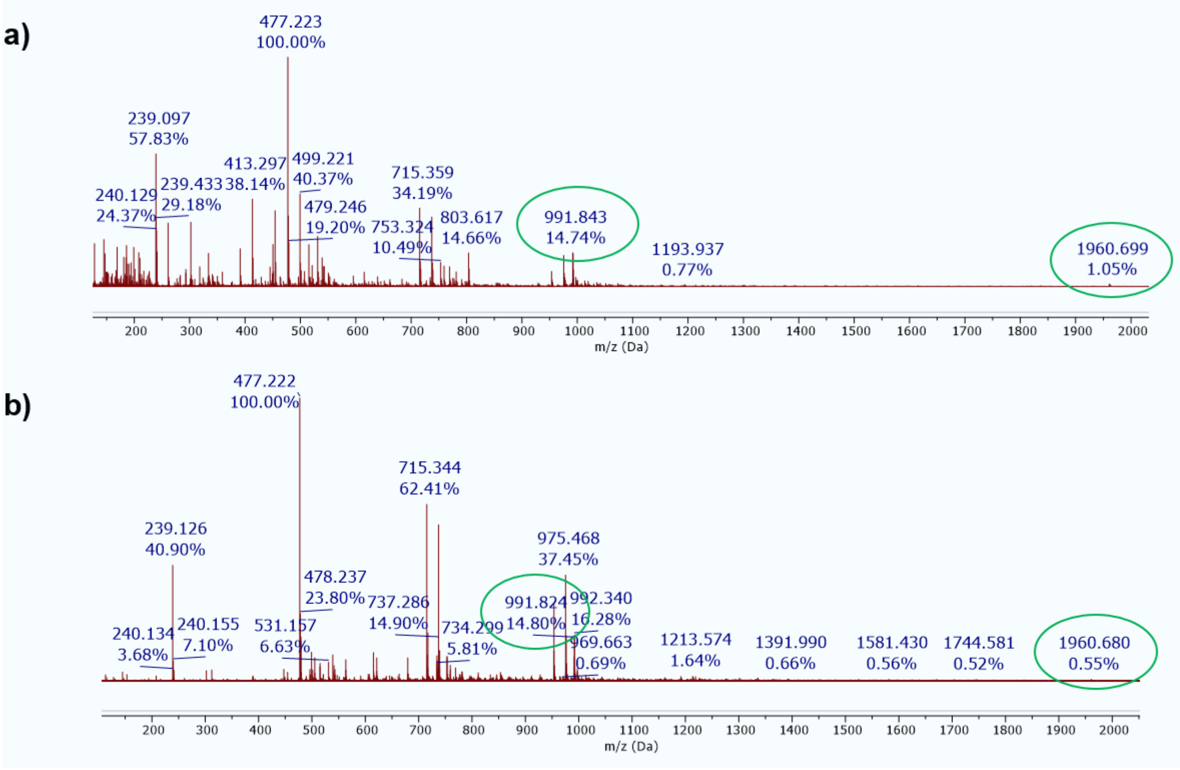

Figure S6-1: Partial MS spectra of 10 µM **XB1** in 10 mM HEPES aqueous buffer solution at pH 7.4. **a)** MS spectrum before addition of 8 equivalents of NaHS. **b)** MS spectrum 1 h after addition of 8 equivalents of NaHS: 1960.7 m/z = **XB1** + Na^+; 991.8 m/z = **XB1** + $2Na^+$; 477.2 m/z = background peak.

S7. Limit of Detection

The limit of detection (LoD) was calculated using the formula:

$$LoD = \frac{3\sigma}{S}$$

σ = Standard deviation of each receptor's emission intensity

S = Initial slope of emission intensity curve on addition of NaHS (M^{-1})

All standard deviations were calculated using five or more emission scans of the receptor (10 µM) before addition of NaHS. Clear outliers were omitted from the calculation.

The slope of the emission intensity curves are graphically demonstrated below, gradient calculations used actual intensities and concentrations of HS^{-}.

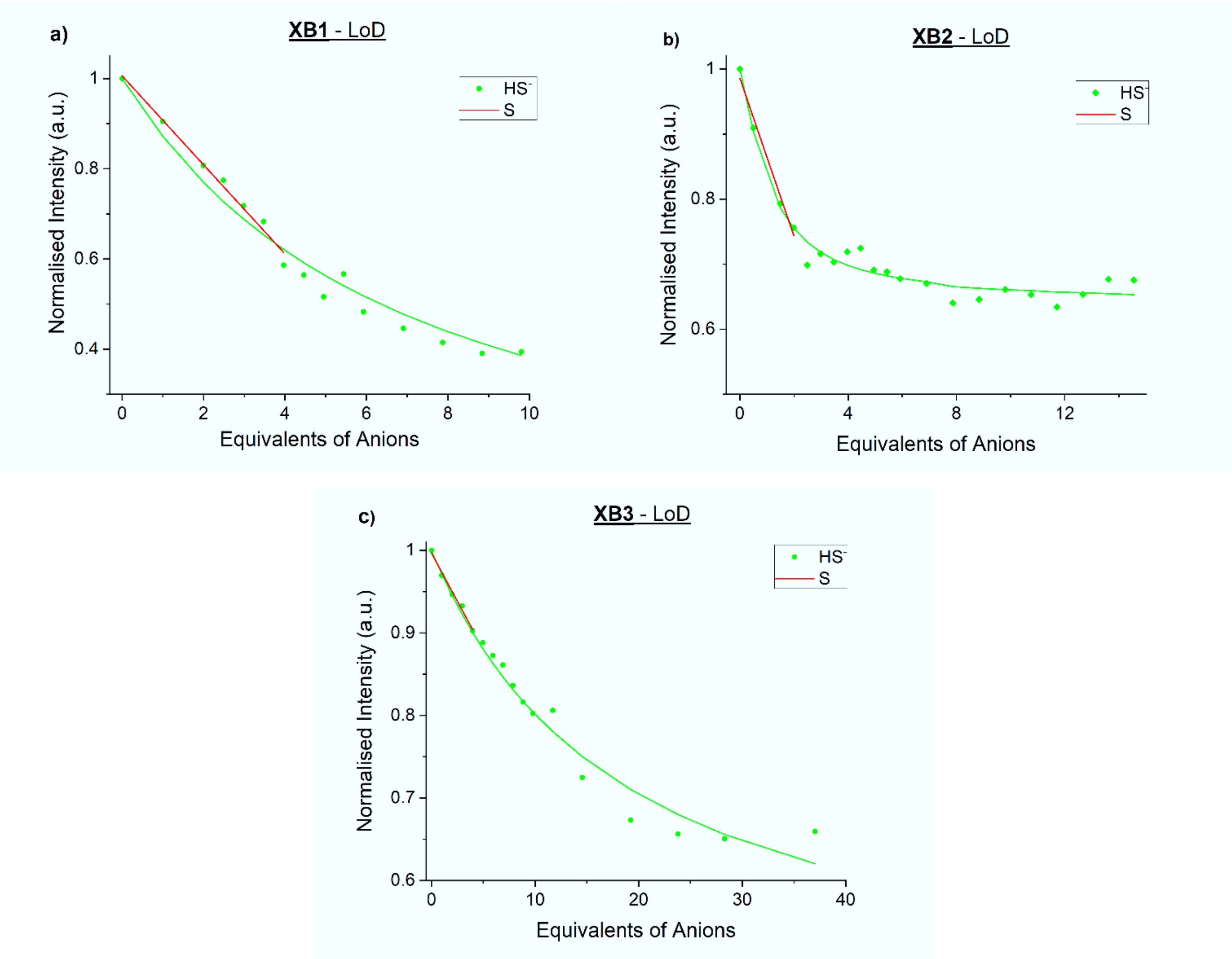

Figure S7-1: BindFit analysis fit of fluorescence titration data monitoring λ_{max} (400 – 420 nm) of receptor **a) XB1 b) XB2 c) XB3** upon addition of NaHS in pH 7.4 10 mM aqueous HEPES buffer solution and the linear line used for the determination of the LoD.

S8. Synthetic and Titration References

[1] C. J. Brassard, X. Zhang, C. R. Brewer, P. Liu, R. J. Clark, L. Zhu; *J Org. Chem*. **2016**, 81, 12091–12105.
[2] K. C. Elbert, D. Jishkariani, Y. Wu, J. D. Lee, B. Donnio, C. B. Murray; *Chem. Mater.* **2017**, 29, 8737–8746
[3] A. A.Kudale, J. Kendall, C. C. Warford, N. D. Wilkins, G. J.Bodwell; *Tetrahedron Lett.*, **2007**, 48, 5077–5080
[4] S. W. Robinson and P. D. Beer, *Org. Biomol. Chem.,* **2017**, 15, 153–159.
[5] T. X. Neenan and G. M. Whitesides, *J. Org. Chem.,* **1988**, 53, 2489–2496.
[6] Xi. Li, J. Y. C. Lim. P. D. Beer; *Chem. Eur. J.*, **2018**, 24,17788–17795
[7] D. Brynn Hibbert and P. Thordarson, *Chem. Commun.*, **2016**, 52, 12792–12805.
[8] http://supramolecular.org/

S9. Molecular Modelling

S9.1 Additional Figures

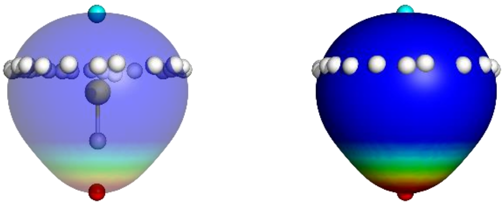

Figure S9-1. Electrostatic potential (V_S) of HS^- (blue, below –135 kcal mol^{-1}; red, above –100 kcal mol^{-1}) mapped on top of the electron density isosurface (0.001 e$^-$ bohr^{-3}) with and without HS^- visible. The less negative V_S value is found in front of H (red sphere) while the second less negative V_S value is located in front of S along the S–H bond (cyan sphere). The white spheres form a rim composed by the most negative V_S values on the surface, which determines the spatial disposition of HS^- relatively to the XB-based model receptors.

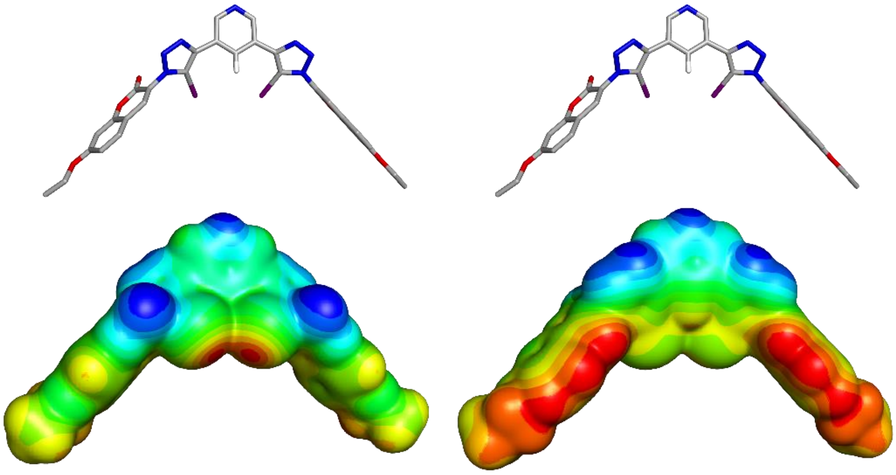

XB1$_{Et}$

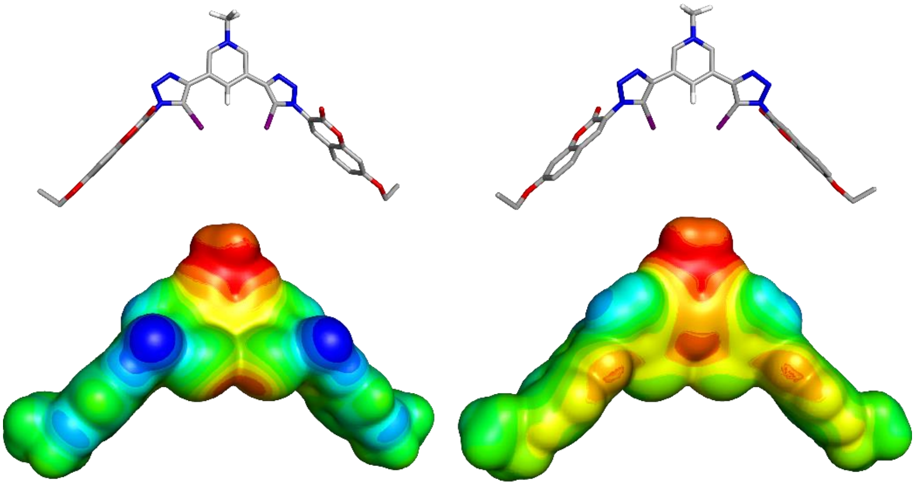

XB2$_{Et}$

Figure S9-2. DFT optimised structures of free model receptors **XB1$_{Et}$** and **XB2$_{Et}$**, in two different views , with the binding units pointing towards or away from the viewer (left and right sides, respectively), together with their electrostatic potentials (V_S) mapped onto the corresponding electron density isosurfaces (0.001 e$^-$ bohr^{-3}). Colour scales range from blue (below –50 kcal mol^{-1}) to red (above 40 kcal mol^{-1}) for neutral **XB1$_{Et}$** and from blue (below –5 kcal mol^{-1}) to red (above 95 kcal mol^{-1}) for positively charged **XB2$_{Et}$**.

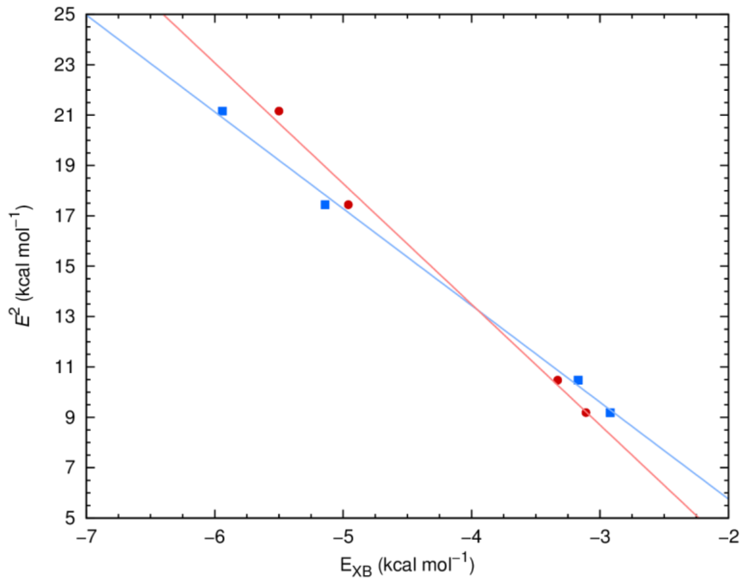

Figure S9-3. XB interactions' E^2 energies *versus* the $E_{XB\text{-}G(r)}$ (■) or $E_{XB\text{-}V(r)}$ (●) interaction energies, together with their corresponding linear fits ($R^2 \geq 0.99$).

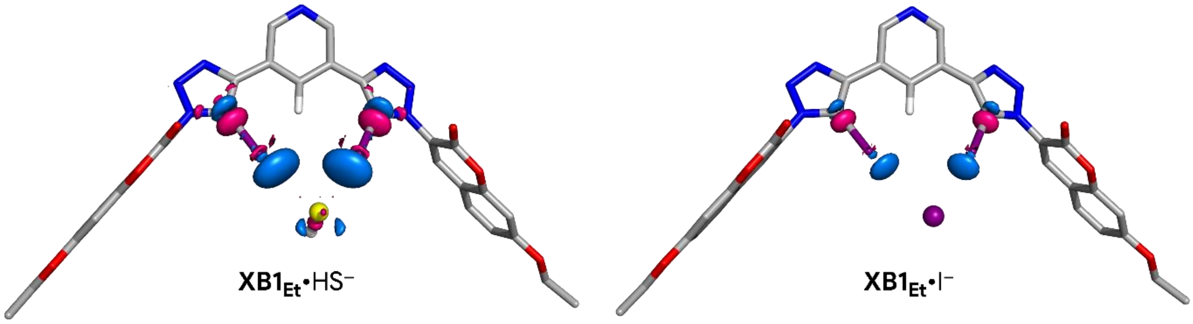

Figure S9-4. Electron density difference maps for the $XB1_{Et}$ associations with HS^- and I^- highlighting the superior electron transfer in the HS^- host-guest association.

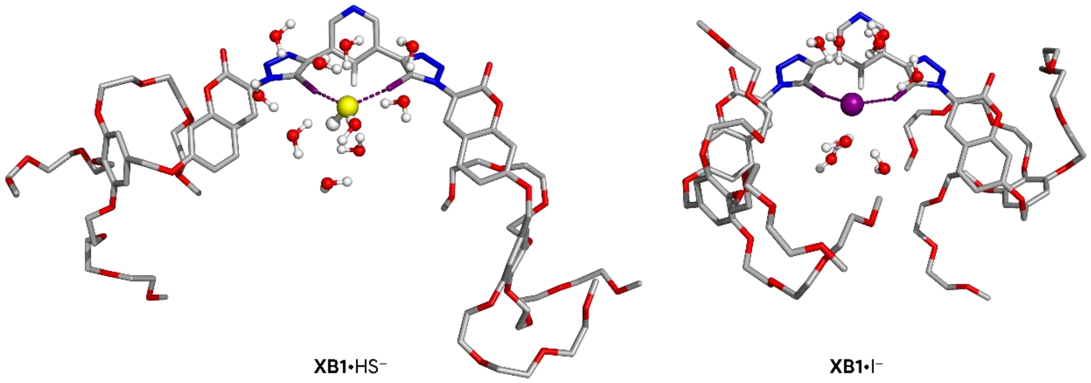

Figure S9-5. Illustrative MD snapshots of **XB1**·HS^- and **XB1**·I^-, showing the anion surrounded by water molecules and the random orientation of the TEG flexible chains. Most hydrogen atoms were hidden for clarity.

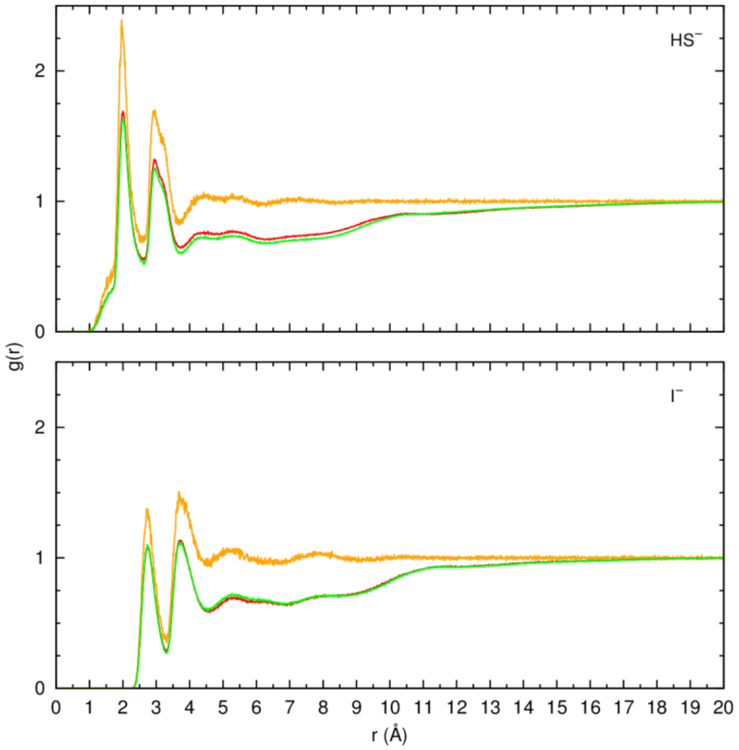

Figure S9-6. Radial distribution function (g(r)) of water around HS^- (top) and I^- (bottom) when associated with **XB1** or **XB2** (red and green lines, respectively), or when freely solvated (orange lines), showing the decrease of the number of water molecules in the 1st and 2nd water hydration spheres upon anion recognition.

S9.2 Additional Tables

Table S9-1. XB dimensions ascertained in the optimised associations of **XB1**$_{Et}$ or **XB2**$_{Et}$ with HS^- or I^-.

Medium	Receptor	Anion	I···A distance (Å)[a]	C–I···A angle (°)[a]	θ angle (°)[b]
C-PCM (water)	**XB1**$_{Et}$	HS^-	3.205; 3.205	171.2; 171.2	90.7
		I^-	3.600; 3.600	173.1; 173.1	–
	**XB2**$_{Et}$	HS^-	3.141; 3.141	171.1; 171.1	93.0
		I^-	3.560; 3.559	173.1; 173.2	–
Gas phase	**XB1**$_{Et}$	HS^-	3.009; 3.009	170.1; 170.1	90.5
		I^-	3.425; 3.425	171.7; 171.7	–
	**XB2**$_{Et}$	HS^-	2.905; 2.905	169.8; 169.8	93.4
		I^-	3.279; 3.279	171.4; 171.4	–

[a] A = SH^- or I^-; [b] the θ angle is the tilt angle between the plane defined by the atoms of the two C–I binding units and the axis of the HS^- anion.

Table S9-2. Uncorrected electronic binding energies ($\Delta\mathcal{E}_0$), Zero Point Corrections (ΔZPE),[a] thermal corrected binding energies (ΔE),[b] binding enthalpies (ΔH),[c] binding entropies term contribution ($T\Delta S$),[d] binding free energies (ΔG_{bind}),[e] and standard state binding free energies (ΔG^{SS})[f] (in kcal mol^{-1}) estimated from the DFT optimised structures of the HS^- and I^- associations with **XB1$_{Et}$** or **XB2$_{Et}$**.

Medium	Receptor	Anion	$\Delta\mathcal{E}_0$	ΔZPE	ΔE	ΔH	$T\Delta S$	ΔG_{bind}	ΔG^{SS}
C-PCM (water)	**XB1$_{Et}$**	HS^-	-14.88	1.15	-13.31	-13.91	-11.60	-2.30	-4.19
		I^-	-8.78	0.22	-7.97	-8.56	-9.68	1.12	-0.77
	**XB2$_{Et}$**	HS^-	-17.24	1.02	-15.70	-16.30	-9.40	-6.90	-8.79
		I^-	-10.22	0.17	-9.40	-10.00	-7.71	-2.29	-4.18
Gas phase	**XB1$_{Et}$**	HS^-	-46.48	1.12	-44.41	-45.00	-7.36	-37.64	-39.53
		I^-	-27.93	0.01	-26.66	-27.25	-5.26	-21.99	-23.88
	**XB2$_{Et}$**	HS^-	-99.33	1.54	-97.58	-98.18	-10.30	-87.87	-89.76
		I^-	-76.33	-0.12	-75.73	-76.32	-7.55	-68.76	-70.65

a) ΔZPE is included in the ΔE, ΔH and ΔG terms; b) $\Delta E = \Delta\mathcal{E}_0 + \Delta E_{Tot}$, where ΔE_{Tot} accounts for the differences in the internal energy due to translational, rotational, vibrational and electronic motions; c) $\Delta H = \Delta E + \Delta nRT$, where n is -1 for a 1:1 host-guest systems, R is the ideal gas constant and T is the temperature (298.15 K); d) $\Delta S = \Delta S_{translational} + \Delta S_{rotational} + \Delta S_{vibrational}$; e) $\Delta G_{bind} = \Delta H - T\Delta S$; f) ΔG^{SS} is the free energy (ΔG_{bind}) including a –1.89 kcal mol^{-1} correction which corresponds to the conversion from the standard state at 1 atm (1 mol per 24.46 L at 298.15 K) to 1 M (1 mol/L at 298.15 K).[2]

Table S9-3. Natural Bond Orbital analysis of the anion associations of **XB1$_{Et}$** or **XB2$_{Et}$**.

Receptor	Anion	E^2 (kcal mol^{-1})[a]	WBI[b]	Change in electron occupancy (e^-)[c]	
				$\sigma^*_{C\text{–}I}$	n_A
**XB1$_{Et}$**	HS^-	17.45; 17.44	0.12; 0.12	0.16	-0.20
	I^-	9.19; 9.19	0.07; 0.07	0.09	-0.11
**XB2$_{Et}$**	HS^-	21.16; 21.16	0.14; 0.14	0.20	-0.24
	I^-	10.47; 10.48	0.08; 0.08	0.10	-0.13

a) XB interactions' Second Order perturbation theory energies between the C–I antibonding orbitals of model receptors and the anions' lone pairs orbitals ($n_A \rightarrow \sigma^*_{C\text{–}I}$); b) Wiberg Bond Indices of the XB interactions; c) Change in the electron occupancy in n_A and $\sigma^*_{C\text{–}I}$ orbitals upon anion binding of HS^- or I^-.

Table S9-4. Quantum Theory of Atoms in Molecules analysis of the XB interactions in the anion associations of **XB1$_{Et}$** or **XB2$_{Et}$**.

Receptor	Anion	ρ(**r**) (×10, ea_0^{-3})^a	$\nabla^2\rho$(**r**) (×10, ea_0^{-3})^b	V(**r**) (×10, Hartree)^c	G(**r**) (×10, Hartree)^d	$E_{XB\text{-}V(r)}$ (kcal mol^{-1})^{e,g}	$E_{XB\text{-}G(r)}$ (kcal mol^{-1})^{f,g}	|V(**r**)|/G(**r**) ratio
**XB1$_{Et}$**	HS$^-$	0.21; 0.21	0.46; 0.46	-0.12; -0.12	0.12; 0.12	-5.14; -5.14	-4.96; -4.96	1.02; 1.02
	I$^-$	0.13; 0.13	0.32; 0.32	-0.07; -0.07	0.07; 0.07	-2.92; -2.92	-3.11; -3.11	0.92; 0.92
**XB2$_{Et}$**	HS$^-$	0.24; 0.24	0.49; 0.49	-0.14; -0.14	0.13; 0.13	-5.94; -5.94	-5.50; -5.50	1.06; 1.06
	I$^-$	0.14; 0.14	0.34; 0.34	-0.07; -0.07	0.08; 0.08	-3.17; -3.17	-3.33; -3.33	0.94; 0.94

$^{a)}$ Electron density; $^{b)}$ Laplacian of the density; $^{c)}$ Potential energy density; $^{d)}$ Lagrangian kinetic energy density; $^{e)}$ Energies of the halogen bonds determined from V(**r**) as $E_{XB\text{-}V(r)}$ = V(**r**) × 0.68; $^{f)}$ Energies of the halogen bonds determined from G(**r**) as $E_{XB\text{-}G(r)}$ = –G(**r**) × 0.67; $^{g)}$ Both correlations were specifically developed to estimate the XB interaction energies involving iodine atoms.[1]

Table S9-5. SAPT decomposition of the interaction energies ($\boldsymbol{E_{SAPT}}$), in the electrostatic ($\boldsymbol{E_{elec}}$), exchange ($\boldsymbol{E_{exch}}$), induction ($\boldsymbol{E_{ind}}$), and dispersion ($\boldsymbol{E_{disp}}$) components, estimated from the gas-phase DFT optimised structures of the HS$^-$ and I$^-$ associations with **XB1$_{Et}$** or **XB2$_{Et}$**, all in kcal mol^{-1}.

Receptor	Anion	E_{elec}	E_{exch}	E_{ind}	E_{disp}	E_{SAPT}
**XB1**	HS$^-$	-59.70	74.00	-45.32	-19.70	-50.72
	I$^-$	-30.70	38.07	-24.57	-14.90	-32.10
**XB2**	HS$^-$	-119.26	94.56	-60.40	-23.18	-108.28
	I$^-$	-83.60	54.96	-36.12	-18.38	-83.13

Table S9-6. Average dimensions, with standard deviations, of the XB interactions assessed in the MD simulations of the associations of **XB1** or **XB2** with HS$^-$ and I$^-$ in water.^{a}

Receptor	Anion	I···A (Å)		C–I···A (°)	
		Avg ± SD	Range	Avg ± SD	Range
**XB1**	HS$^-$	3.262 ± 0.130	[2.402, 4.448]	174.2 ± 2.8	[157.9, 180.0]
	I$^-$	4.245 ± 0.283	[3.193, 10.238]	172.5 ± 4.7	[94.6, 180.0]
**XB2**	HS$^-$	3.181 ± 0.107	[2.401, 7.816]	174.7 ± 2.6	[122.7, 180.0]
	I$^-$	4.166 ± 0.268	[3.495, 11.844]	172.6 ± 5.3	[95.9, 180.0]

$^{a)}$ N = 400000 using the four independent MD runs of 50 ns (50000 frames) with the two XB interactions treated together.

Table S9-7. Average number of water molecules, with standard deviations, surrounding the HS^- or I^- anions at a 3.5 Å cut-off in the MD simulations of their associations with **XB1** or **XB2**[a] or freely solvated.[b]

Receptor	Anion	Avg ± SD	Range
**XB1**	HS^-	8.1 ± 1.3	[3, 14]
	I^-	6.1 ± 1.2	[1, 11]
**XB2**	HS^-	7.8 ± 1.4	[3, 14]
	I^-	6.1 ± 1.1	[1, 11]
Free anions	HS^-	10.6 ± 1.3	[6, 16]
	I^-	7.7 ± 1.0	[4, 13]

[a] N = 200000 using the four independent MD runs of 50 ns (50000 frames); [b] N = 10000, using a single MD run of 10 ns (10000 frames).

S9.3 Molecular Modelling details

General

Quantum calculations were carried out with Gaussian 16 (DFT calculations),[3] while atomic RESP charges derivatisation was carried out with Gaussian 09.[4] The classical force field calculations, including the Molecular Mechanics (MM) energy minimisations and Molecular Dynamics (MD) simulations, were carried out with AMBER 18.[5]

The starting structures of **XB1** and **XB2** and were generated from the single crystal X-ray structure of an analogous of **XB2**, deposited with the Cambridge Crystallographic Data Centre (CCDC)[6] under RefCode WOMZOQ.[7] The methyl pyridinium group was demethylated to afford the pyridine in **XB1**. The tetrafluorophenyl groups were replaced by fluorescent coumarins, functionalised with the TEGylated substituents. The associated I^- was modified to HS^- to afford the corresponding initial binding scenarios. To study the interaction between **XB1** and **XB2** with HS^- and I^- through DFT calculations, the TEG substituted aryl groups were replaced with ethyl moieties, affording the **XB1$_{Et}$** and **XB2$_{Et}$** models.

DFT calculations

The HS^- and I^- associations of the **XB1$_{Et}$** and **XB2$_{Et}$** acyclic model receptors were optimised with the M06-2X functional. The iodine centres were described with the Def2-TZVPD basis sets, while the Def2-TZVP basis set was used for the remaining elements. DFT optimisations were carried out both in gas-phase and with the Conductor-like Polarisable Continuum Model (C-PCM),[8,9] to account for the water solvation effects. The free model receptors and anions were also optimised in the same conditions. The absence of imaginary frequencies on the vibrational frequency analyses confirmed that all stationary point geometries were local minima on the potential energy surface.

The thermochemical data listed in Table S9-2 were computed directly from the structures of the association, receptor, and anion DFT optimised in gas-phase and in C-PCM water model, and the binding free energies (ΔG_{bind}) were calculated using the following equation:

$$\Delta G_{bind} = \Delta E + \Delta nRT - T\Delta S$$

where ΔE is the electronic interaction energy given by $\Delta E = E_{association} - (E_{receptor} + E_{anion})$. The $E_{association}$, $E_{receptor}$ and E_{anion} are the electronic energies of the optimised geometries of XB association, receptor, and anion, including the Zero-Point Energies and Thermal corrections for 298.15 K. The ΔnRT is gas

phase contribution, in which n is –1 for a 1:1 receptor-anion association; R is the ideal gas constant; T is the temperature. Finally, $T\Delta S$ is the energy component that accounts for the differences in entropy due to in the translational, rotational, vibrational, and electronic motions at 298.15 K of the XB association, receptor and anion. ΔG_{bind} was further corrected with the free energy change converting from the standard state at 1 atm (1 mol per 24.46 L at 298.15 K) to 1 M (1 mol/L at 298.15 K), corresponding to –1.89 kcal mol^{-1}, thus affording ΔG^{SS}.[2]

Further analyses of the XB interactions on the C-PCM optimised anion associations were carried out with Natural Bond Orbitals[10] using NBO6[11,12] and with Quantum Theory of Atoms in Molecules[13] using MultiWFN 3.6.[14,15] MultiWFN[14,16] was also used to calculate the distribution of electrostatic potential on the electron density surface (V_S) of the model receptors.

Psi4[17–20] was used to run the Symmetry Adapted Perturbation Theory (SAPT)[21] computations on the gas-phase optimised geometries of the HS^- and I^- associations of **XB1$_{Et}$** and **XB2$_{Et}$**, which were carried out with the same basis-sets (Def2-TZVP and Def2-TZVPD for the iodine centres) and using density fitting. The SAPT approach allows to estimate the interaction energies (E_{SAPT}) with a partitioning into physically meaningful energy components, including the electrostatics (E_{elect}), short-range exchange-repulsion (E_{exch}), induction (E_{ind}), and London dispersion forces (E_{disp}), collected in agreement with the "chemist's grouping" scheme,[22] as follows:

$$E_{SAPT} = E_{elec} + E_{exch} + E_{ind} + E_{disp}$$

Molecular Mechanics and Molecular Dynamics simulations

The Molecular modelling studies in solution were carried out with complete **XB1** and **XB2** associated with HS^- and I^- anions using the General AMBER Force Field (GAFF)[23,24] force field parameters to describe the acyclic receptors and the HS^- anion. The molecular mechanics calculations were preceded by the derivatisation of Restrained Electrostatic Potential (RESP) charges as follows: the **XB1** and **XB2** receptors were optimised at the B3LYP/6-31G(d) level of theory, followed by a single point calculation at HF/6-31G(d) in which the RESP atomic charges were obtained using the Gaussian IOps (6/33 = 2, 6/41 = 4, 6/42 = 6), in agreement with GAFF's development.[23,24] In both the optimisation and single point, the iodine centres were described with the aug-cc-pVDZ-PP basis set. The derivatisation of the HS^- RESP charges was carried out following the same method. The iodide was described with a net charge of −1 and suitable vdW parameters.[25]

Following our previous modelling investigations on anion recognition by XB interactions using a classical MD approach,[26–32] the putative halogen bonds between the receptors and HS^- or I^- were described by the addition of a massless extra point (EP) of charge to GAFF.[33] An EP with a van der Waals parameter and mass set to zero was positioned in front of each iodine binding unit to mimics its σ-hole.

To reproduce the XB interactions previously ascertained in the HS^- or I^- associations of **$XB1_{Et}$** or **$XB2_{Et}$** model receptors by DFT calculations at a higher theory level, several I−EP distances, from 2.00 to 3.50 Å with increments of 0.01 Å, were systematically evaluated in gas-phase by molecular mechanics (MM) through the energy minimisations of the **XB1** and **XB2** anion associations. The I−EP distances were fixed using a high bond stretching force constant of 600 kcal mol^{-1} $Å^{-2}$. An angle bending force constant of 150 kcal mol^{-1} rad^{-2} was used for the C–I–EP ideal angle of 180°. For a given I−EP distance, the RESP atomic charges were re-calculated, as follows: in the previously B3LYP optimised structure of each acyclic host, an EP was linearly positioned in front of each C−I bond at the desired I−EP distance. Subsequently the corresponding atomic charges were calculated *via* a two-stage RESP fitting of the HF obtained electrostatic potential. Out of the tens of I−EP distances tested, it was found that the I−EP distances of 2.24 Å (HS^-) and 3.07 Å (I^-), together with the RESP charges summarised in **Error! Reference source not found.**, allowed to optimise the anion associations of **XB2** in the gas-phase by MM, as well as to undertake the subsequent MD simulations with XB-based complexes immersed in periodic boxes of TIP3P water molecules (see below). For the neutral **XB1**, the I–EP distances of 2.26 and 3.14 Å were found for HS^- and I^-, respectively, and the I and EP charges are also gathered in **Error! Reference source not found.**. The dimensions of the XB interactions assessed in the gas-phase by MM for the **XB1** or **XB2** associations with HS^- or I^- are given in **Error! Reference source not found.**.

Table S9-8. Iodine and EP RESP charges (*e*) in **XB1** and **XB2** for the selected I–EP distances.

Receptor	I−EP distances (Å)	I	EP
**XB1**	no EP	0.107964	−
	2.26	-0.124119	0.073540
	3.14	0.024728	0.019793
**XB2**	no EP	0.210100	−
	2.24	-0.062110	0.081003
	3.07	0.104774	0.023272

Table S9-9. Summary of MM optimised I···A distances (Å) and C–I···A angles (°) (A = SH^- or I^-) in the MM minimised **XB1** and **XB2** associations.

Receptor	Anion	I···A (Å)	C–I···A (°)
**XB1**	HS^-	3.157; 3.152	175.8; 176.1
	I^-	4.041; 3.987	175.4; 175.7
**XB2**	HS^-	3.058; 3.055	176.2; 176.2
	I^-	3.871; 3.867	177.1; 177.5

The four **XB1** or **XB2** associations with HS^- or I^- were solvated in cubic boxes with 12000 TIP3P water molecules.[34] Moreover, the periodic boxes of the **XB1** anion associations were charge neutralised with the addition of a Na^+ counterion, which was described with vdW parameters also taken from ref. [25]. Each solvated system was equilibrated under periodic boundary conditions using the following multistage protocol. The system was relaxed by MM minimisation of solvent molecules and by keeping the solutes fixed with a positional restraint of 500 kcal mol^{-1} $Å^{-2}$. The restraint was then removed, allowing the entire system to relax. These two minimisation stages comprised an initial set of 10000 steepest descent

algorithm steps, followed by 10000 steps of conjugated gradient algorithm. The equilibration proceeded with heating up the system to 300 K for 100 ps using a NVT ensemble and a weak positional restraint (10 kcal mol^{-1} Å^{-2}) on the solutes. Afterwards, each system's density was allowed to equilibrate in an NPT ensemble at 1 atm for 1.0 ns, at the same temperature, followed by a NPT data collection run of 50 ns. The collection run's trajectory frames were saved every 1.0 ps. Four independent runs were performed for each system. The CUDA version of the *PMEMD* executable was used for the simulation of all solvated systems.[35,36] The bond lengths involving all hydrogen atoms were constrained with the SHAKE algorithm, allowing the usage of a 2.0 fs time step.[37] The Particle Mesh Ewald (PME) method was used to treat the long-range electrostatic interactions.[38] The non-bonded van der Waals interactions were truncated with a 10 Å cut-off. The temperature of the system was maintained independently by coupling the system to an external bath temperature of 300 K, using Langevin dynamics,[39] with a collision frequency γ of 1.0 ps^{-1}. The pressure was controlled by the Berendsen barostat[40] at 1 atm and compressibility of 44.6×10^{-6} bar^{-1}, with a relaxation time of 1.0 ps.

For the estimation of the solvation of free HS^- and I^-, the solvated anions neutralised with one Na^+ counterion, were simulated in periodic cubic boxes of 12000 water molecules, for 10 ns (a single MD run) using the same protocol. The post-processing of trajectory files to obtain the structural data and energetic data was performed with *cpptraj*.[41]

S10. Computational References

[1] E. V Bartashevich, V. G. Tsirelson, *Russ. Chem. Rev.* **2014**, *83*, 1181–1203.

[2] C. P. Kelly, C. J. Cramer, D. G. Truhlar, *J. Phys. Chem. B* **2007**, *111*, 408–422.

[3] Gaussian 16, Revision C.01, M. J. Frisch, G. W. Trucks, H. B. Schlegel, G. E. Scuseria, M. A. Robb, J. R. Cheeseman, G. Scalmani, V. Barone, G. A. Petersson, H. Nakatsuji, X. Li, M. Caricato, A. V. Marenich, J. Bloino, B. G. Janesko, R. Gomperts, B. Mennucci, H. P. Hratchian, J. V. Ortiz, A. F. Izmaylov, J. L. Sonnenberg, D. Williams-Young, F. Ding, F. Lipparini, F. Egidi, J. Goings, B. Peng, A. Petrone, T. Henderson, D. Ranasinghe, V. G. Zakrzewski, J. Gao, N. Rega, G. Zheng, W. Liang, M. Hada, M. Ehara, K. Toyota, R. Fukuda, J. Hasegawa, M. Ishida, T. Nakajima, Y. Honda, O. Kitao, H. Nakai, T. Vreven, K. Throssell, J. A. Montgomery, Jr., J. E. Peralta, F. Ogliaro, M. J. Bearpark, J. J. Heyd, E. N. Brothers, K. N. Kudin, V. N. Staroverov, T. A. Keith, R. Kobayashi, J. Normand, K. Raghavachari, A. P. Rendell, J. C. Burant, S. S. Iyengar, J. Tomasi, M. Cossi, J. M. Millam, M. Klene, C. Adamo, R. Cammi, J. W. Ochterski, R. L. Martin, K. Morokuma, O. Farkas, J. B. Foresman, and D. J. Fox, Gaussian, Inc., Wallingford CT, **2016**.

[4] Gaussian 09, Revision D.01, M. J. Frisch, G. W. Trucks, H. B. Schlegel, G. E. Scuseria, M. A. Robb, J. R. Cheeseman, G. Scalmani, V. Barone, G. A. Petersson, H. Nakatsuji, X. Li, M. Caricato, A. Marenich, J. Bloino, B. G. Janesko, R. Gomperts, B. Mennucci, H. P. Hratchian, J. V. Ortiz, A. F. Izmaylov, J. L. Sonnenberg, D. Williams-Young, F. Ding, F. Lipparini, F. Egidi, J. Goings, B. Peng, A. Petrone, T. Henderson, D. Ranasinghe, V. G. Zakrzewski, J. Gao, N. Rega, G. Zheng, W. Liang, M. Hada, M. Ehara, K. Toyota, R. Fukuda, J. Hasegawa, M. Ishida, T. Nakajima, Y. Honda, O. Kitao, H. Nakai, T. Vreven, K. Throssell, J. A. Montgomery, Jr., J. E. Peralta, F. Ogliaro, M. Bearpark, J. J. Heyd, E. Brothers, K. N. Kudin, V. N. Staroverov, T. Keith, R. Kobayashi, J. Normand, K. Raghavachari, A. Rendell, J. C. Burant, S. S. Iyengar, J. Tomasi, M. Cossi, J. M. Millam, M. Klene, C. Adamo, R. Cammi, J. W. Ochterski, R. L. Martin, K. Morokuma, O. Farkas, J. B. Foresman, and D. J. Fox, Gaussian, Inc., Wallingford CT, **2013**.

[5] D.A. Case, I.Y. Ben-Shalom, S.R. Brozell, D.S. Cerutti, T.E. Cheatham, III, V.W.D. Cruzeiro, T.A. Darden, R.E. Duke, D. Ghoreishi, G. Giambasu, T. Giese, M.K. Gilson, H. Gohlke, A.W. Goetz, D. Greene, R Harris, N. Homeyer, Y. Huang, S. Izadi, A. Kovalenko, R. Krasny, T. Kurtzman, T.S. Lee, S. LeGrand, P. Li, C. Lin, J. Liu, T. Luchko, R. Luo, V. Man, D.J. Mermelstein, K.M. Merz, Y. Miao, G. Monard, C. Nguyen, H. Nguyen, A. Onufriev, F. Pan, R. Qi, D.R. Roe, A. Roitberg, C. Sagui, S. Schott-Verdugo, J. Shen, C.L. Simmerling, J. Smith, J. Swails, R.C. Walker, J. Wang, H. Wei, L. Wilson, R.M. Wolf, X. Wu, L. Xiao, Y. Xiong, D.M. York and P.A. Kollman (**2018**), AMBER 2018, University of California, San Francisco..

[6] C. R. Groom, I. J. Bruno, M. P. Lightfoot, S. C. Ward, *Acta Crystallogr. Sect. B Struct. Sci. Cryst. Eng. Mater.* **2016**, *72*, 171–179.

[7] T. Bunchuay, A. Docker, A. J. Martinez‐Martinez, P. D. Beer, *Angew. Chemie Int. Ed.* **2019**, *58*, 13823–13827.

[8] V. Barone, M. Cossi, *J. Phys. Chem. A* **1998**, *102*, 1995–2001.

[9] M. Cossi, N. Rega, G. Scalmani, V. Barone, *J. Comput. Chem.* **2003**, *24*, 669–681.

[10] F. Weinhold, C. R. Landis, *Valency and Bonding: A Natural Bond Orbital Donor-Acceptor Perspective*, Cambridge University Press, **2005**.

[11] E. D. Glendening, C. R. Landis, F. Weinhold, *J. Comput. Chem.* **2013**, *34*, 1429–1437.

[12] E. D. Glendening, C. R. Landis, F. Weinhold, *J. Comput. Chem.* **2013**, *34*, 2134–2134.

[13] R. F. W. Bader, *Atoms in Molecules: A Quantum Theory*, Oxford University Press, **1991**.

[14] T. Lu, F. Chen, *J. Comput. Chem.* **2012**, *33*, 580–592.

[15] T. Lu, F. Chen, *Acta Chim. Sin. - Chinese Ed.* **2011**, *69*, 2393.

[16] T. Lu, F. Chen, *J. Mol. Graph. Model.* **2012**, *38*, 314–323.

[17] D. G. A. Smith, L. A. Burns, A. C. Simmonett, R. M. Parrish, M. C. Schieber, R. Galvelis, P. Kraus, H. Kruse, R. Di Remigio, A. Alenaizan, et al., *J. Chem. Phys.* **2020**, *152*, 184108.

[18] J. Almlöf, K. Faegri, K. Korsell, *J. Comput. Chem.* **1982**, *3*, 385–399.

[19] J. H. Van Lenthe, R. Zwaans, H. J. J. Van Dam, M. F. Guest, *J. Comput. Chem.* **2006**, *27*, 926–932.

[20] S. Lehtola, *J. Chem. Theory Comput.* **2019**, *15*, 1593–1604.

[21] B. Jeziorski, R. Moszynski, K. Szalewicz, *Chem. Rev.* **1994**, *94*, 1887–1930.

[22] E. G. Hohenstein, C. D. Sherrill, *Wiley Interdiscip. Rev. Comput. Mol. Sci.* **2012**, *2*, 304–326.

[23] J. Wang, R. M. Wolf, J. W. Caldwell, P. A. Kollman, D. A. Case, *J. Comput. Chem.* **2004**, *25*, 1157–1174.

[24] J. Wang, R. M. Wolf, J. W. Caldwell, P. A. Kollman, D. A. Case, *J. Comput. Chem.* **2005**, *26*, 114.

[25] P. Li, L. F. Song, K. M. Merz, *J. Chem. Theory Comput.* **2015**, *11*, 1645–1657.

[26] M. J. Langton, S. W. Robinson, I. Marques, V. Félix, P. D. Beer, *Nat. Chem.* **2014**, *6*, 1039–1043.

[27] T. A. Barendt, A. Docker, I. Marques, V. Félix, P. D. Beer, *Angew. Chemie - Int. Ed.* **2016**, *55*, 11069–11076.

[28] J. Y. C. Lim, I. Marques, L. Ferreira, V. Félix, P. D. Beer, *Chem. Commun.* **2016**, *52*, 5527–5530.

[29] J. Y. C. Lim, I. Marques, V. Félix, P. D. Beer, *J. Am. Chem. Soc.* **2017**, *139*, 12228–12239.

[30] J. Y. C. Lim, I. Marques, V. Félix, P. D. Beer, *Angew. Chemie - Int. Ed.* **2018**, *57*, 584–588.

[31] J. Y. C. Lim, I. Marques, V. Félix, P. D. Beer, *Chem. Commun.* **2018**, *54*, 10851–10854.

[32] A. Borissov, I. Marques, J. Y. C. Lim, V. Félix, M. D. Smith, P. D. Beer, *J. Am. Chem. Soc.* **2019**, *141*, 4119–4129.

[33] S. Andersson, M. Sundberg, N. Pristovsek, A. Ibrahim, P. Jonsson, B. Katona, C. M. Clausson, A. Zieba, M. Ramström, O. Söderberg, et al., *Nat. Commun.* **2017**, *8*, 15840.

[34] W. L. Jorgensen, J. Chandrasekhar, J. D. Madura, R. W. Impey, M. L. Klein, *J. Chem. Phys.* **1983**, *79*, 926–935.

[35] R. Salomon-Ferrer, A. W. Götz, D. Poole, S. Le Grand, R. C. Walker, *J. Chem. Theory Comput.* **2013**, *9*, 3878–3888.

[36] S. Le Grand, A. W. Götz, R. C. Walker, *Comput. Phys. Commun.* **2013**, *184*, 374–380.

[37] J. P. Ryckaert, G. Ciccotti, H. J. C. Berendsen, *J. Comput. Phys.* **1977**, *23*, 327–341.

[38] T. Darden, D. York, L. Pedersen, *J. Chem. Phys.* **1993**, *98*, 10089–10092.

[39] R. J. Loncharich, B. R. Brooks, R. W. Pastor, *Biopolymers* **1992**, *32*, 523–535.

[40] H. J. C. Berendsen, J. P. M. Postma, W. F. Van Gunsteren, A. Dinola, J. R. Haak, *J. Chem. Phys.* **1984**, *81*, 3684–3690.

[41] D. R. Roe, T. E. Cheatham, *J. Chem. Theory Comput.* **2013**, *9*, 3084–3095.

Author Contributions

The lead researcher was Edward J. Mitchell who undertook the majority of the synthetic work and all of the titrations, Adam J Beecroft supported the synthetic effort. Both were under the supervision of Paul D Beer, who assisted with the project direction and the writing. Igor Marques and Vítor Félix contributed the computational elements in this report and to the draft according. Finally, Jonathan Martin and Sally Thompson, supported with project administration, external support, and funding acquisition.
